# Data for Tandem Mass Tag (TMT) proteomic analysis of the pancreas during the early phase of experimental pancreatitis

**DOI:** 10.1016/j.dib.2018.08.142

**Published:** 2018-08-30

**Authors:** Violeta García-Hernández, Carmen Sánchez-Bernal, Domitille Schvartz, José J. Calvo, Jean-Charles Sanchez, Jesús Sánchez-Yagüe

**Affiliations:** a Department of Biochemistry and Molecular Biology, University of Salamanca, Salamanca 37007, Spain; b Instituto de Investigación Biomédica de Salamanca (IBSAL), Salamanca 37007, Spain; c Translational Biomarker Group, Department of Human Protein Sciences, University Medical Center, 1211 Geneva, Switzerland; d Department of Physiology and Pharmacology, University of Salamanca, Salamanca 37007, Spain

**Keywords:** Acute pancreatitis, Cerulein, Proteomics, Tandem Mass Tags (TMT), Shotgun proteomics

## Abstract

The quantitative proteomics data reported here pertain to the research article entitled “A Tandem Mass Tag (TMT) proteomic analysis during the early phase of experimental pancreatitis reveals new insights in the disease pathogenesis” (García-Hernández et al., 2018) [1].

The development of acute pancreatitis (AP, an important pathological inflammatory state of the exocrine pancreas) would be based on early changes in protein expression and signaling pathways whose unmasking would be crucial for deciphering AP at the molecular level. We reported here a Tandem Mass Tag (TMT)-based proteomics analysis of rat subcellular fractions of the pancreas during the early phase of experimental AP, using a sixplex isobaric chemical labeling technique. We identified 997 unique proteins, of which 353 were significantly different (22, 276 or 55 in both, the soluble or the membrane fractions, respectively).

Accordingly, using TMT proteomics and bioinformatic tools, in García-Hernández et al., 2018- [1] we were able to detect significant changes in protein expression related to many pathobiological pathways of AP as from the early phase of the disease, including some changes never described before in this disease. Proteomics data are publicly available in ProteomeXchange via PRIDE through the identifier PXD007096.

**Specifications Table**

**Table:** 

Subject area	*Biology*
More specific subject area	*Quantitative proteomics during the early phase of experimental pancreatitis.*
Type of data	*Table of significantly differentially expressed proteins (Isobar R); list of protein identifications and quantifications (Excel files, Supplementary raw and processed MS data).*
How data was acquired	*TMT*^*6*^*plex, ESI-LTQ Orbitrap Velos (Thermo).*
Data format	*MS/MS data analyzed for identification and quantification.*
Experimental factors	*Subcellular fractions (soluble and whole membrane fractions) of the rat pancreas during the early phase of cerulein (Cer)-induced AP were profiled by a shotgun proteomics approach in independent experiments. 50 μg of proteins from each of the six samples was used for the proteomics sample preparation.*
Experimental features	*Trypsin-digested proteins were labelled with the TMT*^*6*^*plex reagents. The pooled labelled sample was fractionated by off-gel electrophoresis (OGE) (12 fractions) prior to LC–MS/MS analysis.*
Data source location	*Salamanca, Spain and Geneva, Switzerland.*
Data accessibility	*MS data have been deposited to the ProteomeXchange Consortium (*http://proteomecentral.proteomexchange.org*) via PRIDE with the identifier* PXD007096.

**Value of the data**

• A shotgun approach is used for the first time to profile the early proteomic alterations in experimental acute pancreatitis (AP), which are considered crucial for its development and for the founding of clinical procedures.
• Our subcellular fractionation protocol allowed us to detect changes in membrane proteins so far overlooked in the proteomic study of AP, as well as the regulation in the expression of soluble/secreted and plasmatic proteins that could be good candidates as early biomarkers of the disease.
• This data describes protein changes related to many pathobiological pathways of AP as from the early phase of the disease, including vesicle-mediated and protein transport, lysosomal and mitochondrial impairment or proteolysis.

## 1 Data

The data reported here includes a shotgun proteomic experiment involving sixplex Tandem Mass Tag (TMT^6^) labeling that allowed the simultaneous identification and quantification of the early phase of AP proteome from the soluble and the whole membrane pancreatic fractions from three AP and three control animals [1].

A total of 997 different proteins were identified with at least two distinct peptides (555 and 686 in the soluble or the whole membrane fractions, respectively). Of them, 353 were significantly different (22, 276 or 55 in both, the soluble or the membrane fractions, respectively).

## 2 Experimental design, materials and methods

### 2.1 Induction of AP and preparation of samples

The early phase of AP was induced in Male Wistar rats as described previously [2]. Briefly, animals received 2 s.c. injections of 20 μg Cer (Sigma-Aldrich, St. Louis, MO, USA)/kg body weight or its vehicle (0.9% NaCl), respectively, at hourly intervals. At 2 h after the first injection, the animals were anesthetized with sodium pentobarbital (100 mg/kg), blood samples were taken by cardiac puncture and animals were then sacrificed. The pancreata, which were used immediately for experiments, were rapidly dissected out from the surrounding fat tissue and lymph nodes, and their wet weights rapidly measured. The remaining parts of the pancreata were homogenized individually with a Potter Elvehjem device in 4 ml of homogenization buffer (5 mM imidazole buffer, pH 7.4 containing 1 mM EDTA, 1 mM PMSF, 100 μg/ml trypsin inhibitor, 100 μM TPCK and TLCK, 2 μg/ml Protease Inhibitor Cocktail, 1 mM NaF 1 and 1 mM Na_3_VO_4_). Homogenization buffer was then added to reach a ratio of 10 volumes (w/v), mixed well and the total homogenate was centrifuged at 150 g for 15 min. The pellet was discarded and the postnuclear homogenate of each pancreas was finally centrifuged at 150,000*g* for 60 min to obtain the soluble and the whole membrane fractions. The surface of the pellet corresponding to this latter fraction was gently washed once with homogenization buffer with a view to minimize contamination with the soluble fraction. Both fractions were independently used for the proteomic analysis. Protein concentrations were assayed by the method of Bradford [3] using BSA as standard. Quality control of the assays was ensured by repeating them at least three times with five different volumes of three-to-five different sample dilutions.

### 2.2 Proteomics/quantitative TMT analysis

Tandem Mass Tag-based proteomics were performed as described by Dayon et al. [4] with slight modifications as indicated below.

#### 2.2.1 Sample preparation and TMT-^6^plex labeling

Three control and three pancreatitis samples from both the soluble and the whole membrane fractions were randomly selected to perform independent TMT^6^plex experiments. To optimize protein concentration and to assess sample quality, we performed SDS-PAGE and silver staining as described previously [5], [6]. Fifty μg of proteins from each of the six samples were dried under vacuum, dissolved in 0.1 M TEAB, 0.1% RapiGest SF surfactant (Waters, Milford, USA) and spiked with 0.5 μg of bovine β-lactoglobulin (LACB) as an internal standard for later correction of experimental bias. The protein samples were reduced in TCEP, alkylated in IAA and then digested using trypsin (trypsin/protein ratio 1:20) as previously described [4]. The resulting peptide mixtures were labeled with one of the TMT reagents from the sixplex version (Thermo Scientific, Rockford, IL) according to the manufacturer´s protocol. Control samples C1, C2, C3 were tagged with TMTs 126.1, 127.1 and 128.1 and AP samples AP1, AP2, AP3 with TMTs 129.1, 130.1 and 131.1, respectively. After labeling, the six samples from each.

TMT^6^ experiment were combined and acidified with trifluoroacetic acid to hydrolize the RapiGest surfactant. RapiGest was then removed according to the manufacturer׳s instructions and the supernatants dried on a SpeedVac.

The labeled peptide mixtures were desalted using C18 Macro Spin Columns (Harvard Apparatus). After peptide elution, samples were dried under vacuum. Peptides were then fractionated by off-gel electrophoresis (OGE) according to their pI on an Agilent 3100 OFFGEL fractionator (Agilent Technologies, Santa Clara, CA) following the manufacturer׳s manual. Isoelectricfocusing was performed on a commercial 13 cm IPG pH 3–10 linear dry strip (GE Healthcare Biosciences AB, Uppsala, Sweden) with a 12 wells frame set. After focusing, the 12 fractions were recovered individually and cleaned using C18 microspin columns (Harvard Apparatus, Holliston, MA). Eluted peptides were finally evaporated and stored at − 20 °C prior MS analysis.

#### 2.2.2 LC–MS/MS analysis

The LC–MS/MS analysis was performed as described elsewhere [5]. Each peptidic fraction was reconstituted in 5% ACN, 0.1% TFA. ESI LTQ-OT MS was performed on a LTQ Orbitrap velos from Thermo Electron (San Jose, CA, USA) equipped with a NanoAcquity system from Waters. Peptides were trapped on a home-made 5 μm 200 Å Magic C18 AQ (Michrom) 0.1 × 20 mm pre-column and separated on a home-made 5 μm 100 Å Magic C18 AQ (Michrom) 0.75 × 150 mm column. The analytical separation was run for 65 min using a gradient of H_2_O/FA 99.9%/0.1% (solvent A) and CH_3_CN/FA 99.9%/0.1% (solvent B). For MS survey scans, the OT resolution was set to 60,000 and the ion population was set to 5 × 10^5^ with an *m*/*z* window from 400 to 2000. Maximum of 3 precursors were selected for both collision induced dissociation (CID) in the LTQ and high-energy C-trap dissociation (HCD) with analysis in the OT. For MS/MS in the LTQ, the ion population was set to 7000 for velos while for MS/MS detection in the OT, it was set to 2 × 10^5^, with resolution of 7500, first mass at *m*/*z* = 100, and maximum injection time of 750 ms. The normalized collision energies were set to 35% for CID and 60% for HCD.

#### 2.2.3 Proteomics data analysis

MS/MS data were analysed for protein identifications using EasyProt v.2.3 [7]. A total of 12 raw files were obtained. Peak lists were generated into.mgf format with EasyProtConv, and CID/HCD merging was used to improve peptide identification and quantification [7]. The resulting 12.mgf files were merged into a single.mgf file that was searched against UniProt/Swiss-Prot database (UniProtKB release 2014_10 of Oct, 29, 2014), selecting the following parameters: a) Rattus norvegicus taxonomy; b) instrument type was set to ESI-LTQ Orbitrap with CID_LTQ_scan_LTQ as the scoring model; c) trypsin as the proteolytic enzyme, with one missed cleavage allowed and the normal cleavage mode; d)TMT^6^ amino-terminus, TMT^6^ lysine and cysteine carbamidomethylations as fixed modifications, and oxidation of methionine as variable; e) precursor ion tolerance was set to 10 ppm; f) minimum peptide length was set to 6 amino acids; g) minimum peptide z-score was set to 4. The technical efficiency of the TMT^6^ experiments was assessed by the peptide labeling rate and the peptide relative intensity distribution of LACB among the 6 tags. Only proteins with at least two unique peptide sequences and a false discovery rate (FDR) ≤ 1% [7] were selected for further quantification. Proteins were clustered based on shared peptides indistinguishable by MS. Quantification was conducted using Isobar R package v.1.9.3.2. [8]. Isotopic purity correction (according to the developer׳s algorithm; the authors declare no conflict of interest) and Isobar default normalization were applied for each TMT^6^ reporter ion intensity. For both TMT^6^ experiments, the protein ratio AP/C was computed as 129+130+131 over 126+127+128. Differential protein expression was considered whenever both ratio *p*-value (an estimator of ratio accuracy) and sample *p*-value (an estimator of biological variability and significance) were lower than 0.05 [9].

The relevant MS data have been deposited to the ProteomeXchange Consortium (http://proteomecentral.proteomexchange.org) via PRIDE with the identifier PXD007096 [10].

All protein identification and quantification details including statistics are available in Supporting information files S1 and S2.
