# Supplementary material for: Data for Tandem Mass Tag (TMT) proteomic analysis of the pancreas during the early phase of experimental pancreatitis
Source: Data Brief. 2018 Aug 30;20:779–83. doi: 10.1016/j.dib.2018.08.142 (PMC6129721; doi:10.1016/j.dib.2018.08.142)
Supplement: Supplementary file 1 — Supplementary material [file mmc1.pdf]

Isobar QC Report - SOLUBLE FRACTION*

December 17, 2014

Reporter Mass Precision

Histogram representing the distribution of the delta mass (in m/z) for each reporter tag between theoretical and observed mass. Reporter tag ions were extracted from MS2 fragment spectra at ± 0.05 m/z around the theoretical masses.

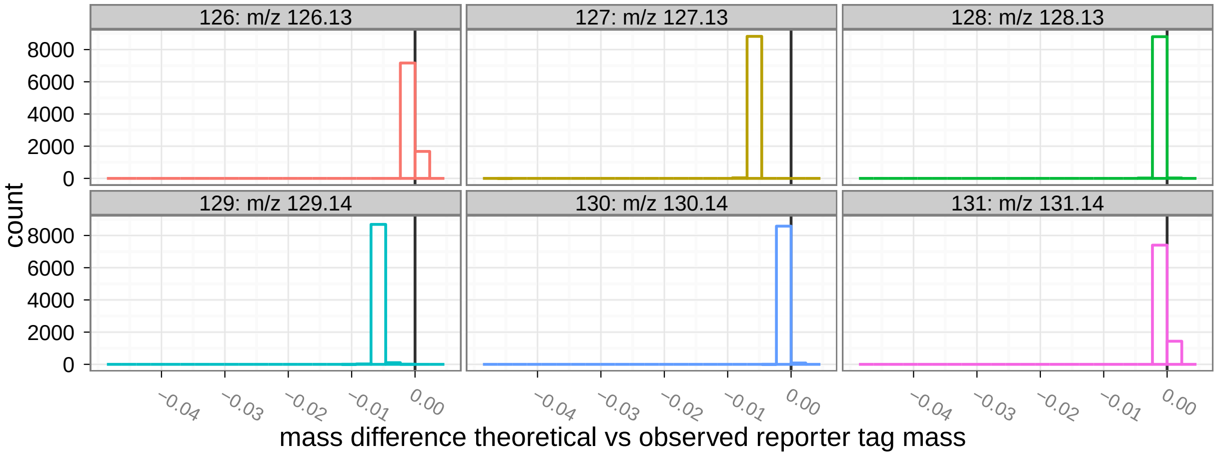

Reporter Intensities Normalization

Box-plot of the reporter intensities before and after the normalization process. Isobar applies a normalization factor to impose equal median or summed intensity in each channel. Some channels may be excluded from normalization, as defined in the `properties.R`.

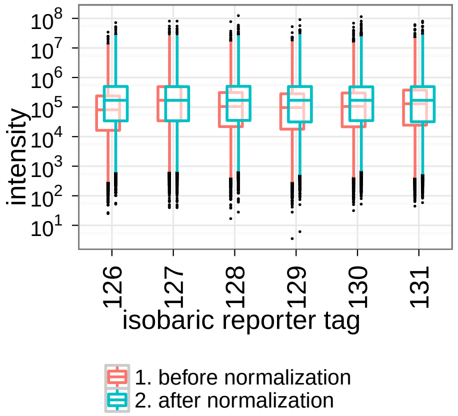

		before norm.		after norm.		
tag	NA[a]	mean	median	mean	median	factor[b]
126	0.35	281879.5	80770.5	590775.4	169282.3	0.4771
127	0.18	568178.9	169282.3	568178.9	169282.3	1
128	0.28	389229.6	104146.5	632663.3	169282.3	0.6152
129	0.57	339107.1	96309.4	596046.3	169282.3	0.5689
130	2.31	412774.1	104623.6	667873.9	169282.3	0.618
131	0.39	479893.2	129700.7	626345.1	169282.3	0.7662

[a] Percentage of spectra with no quantitative information
[b] n.n. ... excluded from normalization

*This report was generated using the `isobar` R package version 1.9.3.2 [built using R 2.14.1; ; 2014-02-07 09:57:05 UTC; unix]. If you use it in published work, please cite 'Breitwieser FP *et al.*: General statistical modeling of data from protein relative expression isobaric tags, *Journal of Proteome Research* 2011' and 'Breitwieser FP and Colinge J: isobarPTM: A software tool for the quantitative analysis of post-translationally modified proteins,*Journal of Proteomics* 2013'

Ratio Distribution

Distribution of the computed protein ratios (bars) and the probability density function of a Cauchy distribution (solid line) fitted as described below.

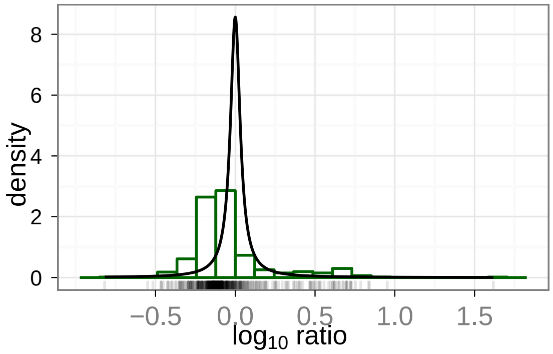


```
Distribution Object of Class: Cauchy
 location: 0
 scale: 0.03715
```

probabilities:	0.5%	1%	2.5%	5%	95%	97.5%	99%	99.5%
distribution quantiles (log_{10}):	-2.36	-1.18	-0.47	-0.23	0.23	0.47	1.18	2.36
(normal scale):	0.004	0.066	0.337	0.583	1.716	2.965	15.205	231.509

Individual Ratio Distributions

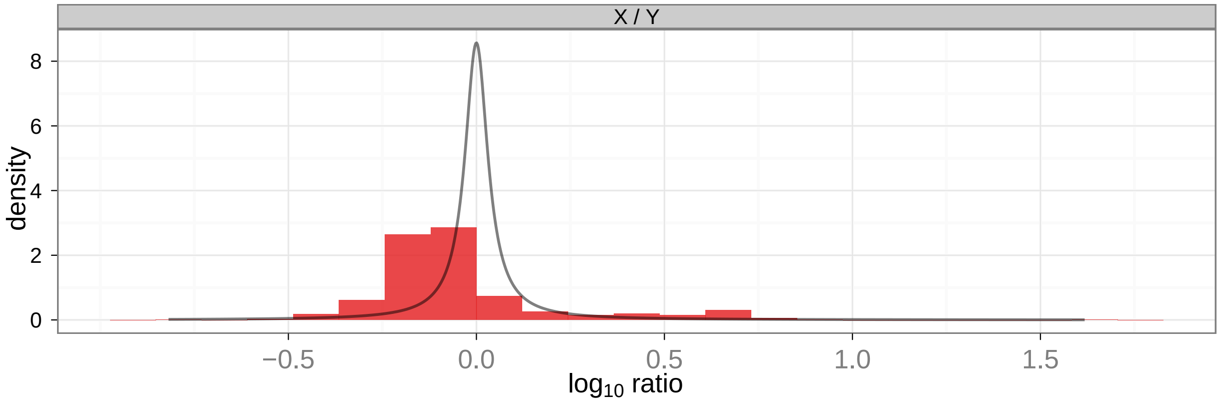

	class2	class1	res[[1]]
1	X	Y	549
2	X	Y	234
3	X	Y	64

Ratio-intensity Plot

Display of the ratio (*y-axis*) versus the log10 average signal intensity (*x-axis*) for all the reporter ratio combinations. The noise model used by Isobar is indicated as a solid red line.

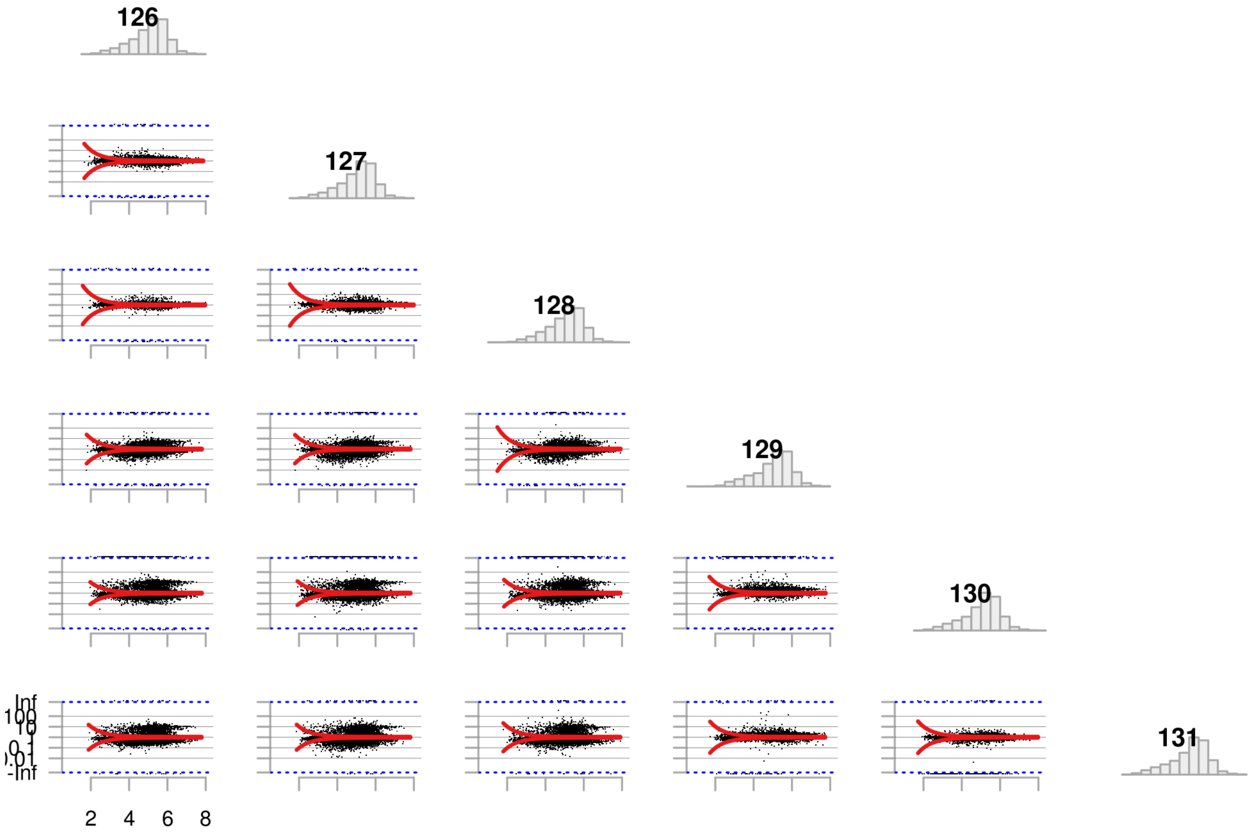
126
127
128
129
130
131
Inf
100
10
1
0.1
0.01
-Inf
2
4
6
8

Isobaric Tag Quantification Report*
SOLUBLE FRACTION

December 17, 2014

Channel	Class
126	*Y*
127	*Y*
128	*Y*
129	*X*
130	*X*
131	*X*

Column Descriptions	
protein	Protein name, accession code, and description.
group	Protein group size, only displayed if it is not '1/1'. First number: Number of group reporters; second number: group size. See Protein Group Details for details.
peptides	Number of peptides for protein.
spectra	Number of idenfitied spectra for protein.
quant	Number of spectra with quantitative information for *ch2* versus *ch1*.
ratio	Ratio of protein in channel *ch2* relative to *ch1*.
*	Significance of protein ratio in sample.

1 Significantly Regulated Proteins X / Y

Number of significant proteins:

#	protein	group	peptides	spectra	quant	ratio
1	Pecr Q9WVK3: Peroxisomal trans-2-enoyl-CoA reductase		2	3	2	**0.15**
2	Mdh2 P04636: Malate dehydrogenase, mitochondrial		13	42	35	**0.28**
3	Etfb Q68FU3: Electron transfer flavoprotein subunit beta		9	13	8	**0.30**
4	Hspe1 P26772: 10 kDa heat shock protein, mitochondrial		6	12	8	**0.34**
5	Sod2 P07895: Superoxide dismutase [Mn], mitochondrial		2	10	3	**0.34**
6	Idh2 P56574: Isocitrate dehydrogenase [NADP], mitochondrial		21	41	36	**0.34**
7	Aldh6a1 Q02253: Methylmalonate-semialdehyde dehydrogenase [acylating], mitochondrial		16	31	27	**0.35**
8	Hadh Q9WVK7: Hydroxyacyl-coenzyme A dehydrogenase, mitochondrial		7	12	12	**0.38**
9	Aldh2 P11884: Aldehyde dehydrogenase, mitochondrial		13	23	17	**0.38**
10	Gatm P50442: Glycine amidinotransferase, mitochondrial		21	43	38	**0.39**
11	P56571: ES1 protein homolog, mitochondrial		2	2	2	**0.40**
12	Psmd13 B0BN93: 26S proteasome non-ATPase regulatory subunit 13		2	2	2	**0.40**

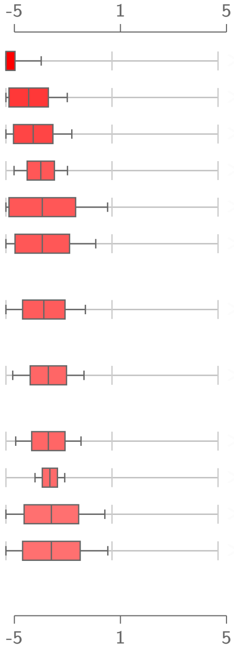

*This report was generated using the **isobar** R package version 1.9.3.2 [built using R 2.14.1; ; 2014-02-07 09:57:05 UTC; unix]. If you use it in published work, please cite 'Breitwieser FP *et al.*: General statistical modeling of data from protein relative expression isobaric tags, *Journal of Proteome Research* 2011' and 'Breitwieser FP and Colinge J: isobarPTM: A software tool for the quantitative analysis of post-translationally modified proteins,*Journal of Proteomics* 2013'

#	protein	group	peptides	spectra	quant	ratio
13	Pla2g1b P04055: Phospholipase A2		6	25	19	**0.42**
14	Acat1 P17764: Acetyl-CoA acetyltransferase, mitochondrial		12	37	32	**0.43**
15	Acadm P08503: Medium-chain specific acyl-CoA dehydrogenase, mitochondrial		2	2	2	**0.44**
16	Echdc3 Q3MIE0: Enoyl-CoA hydratase domain-containing protein 3, mitochondrial		3	3	3	**0.44**
17	Sardh Q64380: Sarcosine dehydrogenase, mitochondrial		3	3	3	**0.45**
18	Ngfg P00758: Kallikrein-1		3	4	3	**0.45**
19	Anxa3 P14669: Annexin A3		2	3	3	**0.45**
20	Got2 P00507: Aspartate aminotransferase, mitochondrial		6	8	7	**0.45**
21	Oxct1 B2GV06: Succinyl-CoA:3-ketoacid coenzyme A transferase 1, mitochondrial		2	2	2	**0.46**
22	Apoa1bp B0BNM1: NAD(P)H-hydrate epimerase		2	4	3	**0.46**
23	Hspa9 P48721: Stress-70 protein, mitochondrial		8	10	8	**0.46**
24	Serpinb1a Q4G075: Leukocyte elastase inhibitor A		16	61	51	**0.47**
25	Capn2 Q07009: Calpain-2 catalytic subunit		5	6	5	**0.47**
26	Hmgcl P97519: Hydroxymethylglutaryl-CoA lyase, mitochondrial		4	4	3	**0.48**
27	Acaa2 P13437: 3-ketoacyl-CoA thiolase, mitochondrial		3	3	3	**0.48**
28	Tst P24329: Thiosulfate sulfurtransferase		5	12	8	**0.49**
29	Bcat2 O35854: Branched-chain-amino-acid aminotransferase, mitochondrial		11	30	26	**0.50**
30	Spink3 P09656: Serine protease inhibitor Kazal-type 3		2	4	3	**0.50**
31	Anxa6 P48037: Annexin A6		27	46	38	**0.50**
32	Hibadh P29266: 3-hydroxyisobutyrate dehydrogenase, mitochondrial		3	5	4	**0.51**
33	Ran P62828: GTP-binding nuclear protein Ran	1/2	7	17	2	**0.51**
34	Ctbp1 Q9Z2F5: C-terminal-binding protein 1		2	2	2	**0.51**
35	Fh P14408: Fumarate hydratase, mitochondrial		5	10	9	**0.52**
36	Dhtkd1 Q4KLP0: Probable 2-oxoglutarate dehydrogenase E1 component DHKTD1, mitochondrial		3	4	3	**0.52**
37	Aco2 Q9ER34: Aconitate hydratase, mitochondrial		19	33	29	**0.52**
38	Actr1a P85515: Alpha-centractin		3	3	3	**0.52**
39	Etfa P13803: Electron transfer flavoprotein subunit alpha, mitochondrial		10	19	14	**0.52**
40	Ivd P12007: Isovaleryl-CoA dehydrogenase, mitochondrial		7	16	14	**0.52**
41	Hnrnpf Q794E4: Heterogeneous nuclear ribonucleoprotein F		7	10	7	**0.53**
42	Dld Q6P6R2: Dihydrolipoyl dehydrogenase, mitochondrial		9	16	12	**0.54**
43	Gsta5 P46418: Glutathione S-transferase alpha-5		2	2	1	**0.54**
44	Try3 P08426: Cationic trypsin-3		6	55	39	**0.54**
45	Ptma P06302: Prothymosin alpha [Cleaved into: Prothymosin alpha, N-terminally processed; . . .		3	5	3	**0.54**
46	Acadl P15650: Long-chain specific acyl-CoA dehydrogenase, mitochondrial		2	3	3	**0.54**
47	Hspd1 P63039: 60 kDa heat shock protein, mitochondrial		17	29	21	**0.54**
48	Gcsh Q5I0P2: Glycine cleavage system H protein, mitochondrial		2	2	2	**0.55**

#	protein	group	peptides	spectra	quant	ratio
49	Atg7 Q641Y5: Ubiquitin-like modifier-activating enzyme ATG7		2	3	3	**0.56**
50	Mpst P97532: 3-mercaptopyruvate sulfurtransferase		5	12	9	**0.57**
51	Cpq Q6IRK9: Carboxypeptidase Q		2	2	2	**0.57**
52	Ybx3 Q62764: Y-box-binding protein 3		4	5	3	**0.58**
53	C1qbp O35796: Complement component 1 Q subcomponent-binding protein, mitochondrial		3	6	5	**0.58**
54	Lum P51886: Lumican		7	11	8	**0.58**
55	Ppp2r1b Q4QQT4: Serine/threonine-protein phosphatase 2A 65 kDa regulatory subunit A beta isoform		2	3	3	**0.58**
56	Calm1 P62161: Calmodulin	1/2	4	6	2	**0.58**
57	Hnrnpa1 P04256: Heterogeneous nuclear ribonucleoprotein A1		4	5	4	**0.58**
58	Bdh2 D4A1J4: 3-hydroxybutyrate dehydrogenase type 2		5	7	5	**0.59**
59	Rplp0 P19945: 60S acidic ribosomal protein P0		2	5	5	**0.59**
60	Prdx5 Q9R063: Peroxiredoxin-5, mitochondrial		10	18	13	**0.59**
61	Ca3 P14141: Carbonic anhydrase 3		7	11	10	**0.59**
62	Mccc2 Q5XIT9: Methylcrotonoyl-CoA carboxylase beta chain, mitochondrial		2	2	2	**0.59**
63	Ufm1 Q5BJP3: Ubiquitin-fold modifier 1		2	8	6	**0.59**
64	Amy2 P00689: Pancreatic alpha-amylase		27	416	308	**0.59**
65	Eif4e P63074: Eukaryotic translation initiation factor 4E		3	3	3	**0.59**
66	Rbbp9 O88350: Putative hydrolase RBBP9		3	4	4	**0.59**
67	Q5U2Q3: Ester hydrolase C11orf54 homolog		5	9	8	**0.60**
68	Hmbs P19356: Porphobilinogen deaminase		5	5	4	**0.60**
69	Pura P86252: Transcriptional activator protein Pur-alpha		5	10	8	**0.60**
70	Ppp6c Q64620: Serine/threonine-protein phosphatase 6 catalytic subunit		2	3	3	**0.61**
71	Anxa4 P55260: Annexin A4		6	7	5	**0.61**
72	Ostf1 Q6P686: Osteoclast-stimulating factor 1		3	6	5	**0.61**
73	Nit2 Q497B0: Omega-amidase NIT2		9	13	8	**0.61**
74	Hnrnpd Q9JJ54: Heterogeneous nuclear ribonucleoprotein D0		5	9	6	**0.61**
75	Suclg1 P13086: Succinyl-CoA ligase [ADP/GDP-forming] subunit alpha, mitochondrial		4	4	4	**0.61**
76	Cpa2 P19222: Carboxypeptidase A2		15	128	123	**0.62**
77	Tubb3 Q4QRB4: Tubulin beta-3 chain		7	25	1	**0.62**
78	Aldh16a1 Q3T1L0: Aldehyde dehydrogenase family 16 member A1		2	3	3	**0.62**
79	Lap3 Q68FS4: Cytosol aminopeptidase		12	16	14	**0.62**
80	Akr1b1 P07943: Aldose reductase		14	24	20	**0.63**
81	Glrx3 Q9JLZ1: Glutaredoxin-3		4	8	6	**0.63**
82	Psme2 Q63798: Proteasome activator complex subunit 2		2	2	2	**0.64**
83	Cap1 Q08163: Adenylyl cyclase-associated protein 1		4	7	5	**0.64**
84	Isoc1 Q6I7R3: Isochorismatase domain-containing protein 1		2	3	3	**0.64**
85	Nap1l4 Q5U2Z3: Nucleosome assembly protein 1-like 4		4	6	3	**0.64**
86	Esd B0BNE5: S-formylglutathione hydrolase		6	22	22	**0.64**
87	Ctrb1 P07338: Chymotrypsinogen B		10	99	77	**0.65**

#	protein	group	peptides	spectra	quant	ratio
88	Cdc42 Q8CFN2: Cell division control protein 42 homolog		6	6	5	**0.65**
89	Dcps Q8K4F7: m7GpppX diphosphatase		3	3	3	**0.65**
90	Cela2a P00774: Chymotrypsin-like elastase family member 2A		9	234	164	**0.65**
91	Cpa1 P00731: Carboxypeptidase A1		19	172	147	**0.66**
92	Rexo2 Q5U1X1: Oligoribonuclease, mitochondrial		3	6	5	**0.66**
93	Hagh O35952: Hydroxyacylglutathione hydrolase, mitochondrial		3	5	3	**0.66**
94	Hrsp12 P52759: Ribonuclease UK114		4	10	10	**0.66**
95	Myh10 Q9JLT0: Myosin-10		4	5	1	**0.66**
96	Pc P52873: Pyruvate carboxylase, mitochondrial		2	2	2	**0.66**
97	Eci1 P23965: Enoyl-CoA delta isomerase 1, mitochondrial		3	3	3	**0.66**
98	Gsr P70619: Glutathione reductase		2	3	3	**0.66**
99	Cpb1 P19223: Carboxypeptidase B		15	171	121	**0.67**
100	Dcxr Q920P0: L-xylulose reductase		2	2	2	**0.67**
101	Sod1 P07632: Superoxide dismutase [Cu-Zn]		9	17	14	**0.67**
102	Nutf2 P61972: Nuclear transport factor 2		2	2	2	**0.67**
103	Hnrnph2 Q6AY09: Heterogeneous nuclear ribonucleoprotein H2		6	9	2	**0.67**
104	S100a11 Q6B345: Protein S100-A11		2	5	3	**0.67**
105	Dbi P11030: Acyl-CoA-binding protein		5	20	16	**0.67**
106	Hmgcs2 P22791: Hydroxymethylglutaryl-CoA synthase, mitochondrial		3	3	3	**0.67**
107	Prss1 P00762: Anionic trypsin-1		3	77	57	**0.68**
108	Akr1a1 P51635: Alcohol dehydrogenase [NADP(+)]		8	16	15	**0.68**
109	Pnp P85973: Purine nucleoside phosphorylase		9	10	8	**0.68**
110	Tkt P50137: Transketolase		19	39	28	**0.68**
111	Ctsb P00787: Cathepsin B		2	2	2	**0.68**
112	Gdi1 P50398: Rab GDP dissociation inhibitor alpha		9	15	6	**0.68**
113	Aldh9a1 Q9JLJ3: 4-trimethylaminobutyraldehyde dehydrogenase		12	22	19	**0.68**
114	Osgep Q9WVS2: Probable tRNA N6-adenosine threonylcarbamoyltransferase		2	3	3	**0.68**
115	Pafah1b1 P63004: Platelet-activating factor acetylhydrolase IB subunit alpha		5	7	6	**0.68**
116	Pygb P53534: Glycogen phosphorylase, brain form	1/2	5	6	3	**0.69**
117	Aars P50475: Alanine--tRNA ligase, cytoplasmic		15	24	16	**0.69**
118	Xpnpep1 O54975: Xaa-Pro aminopeptidase 1		4	4	3	**0.69**
119	Pdia3 P11598: Protein disulfide-isomerase A3		18	34	29	**0.69**
120	Bpnt1 Q9Z1N4: 3'(2'),5'-bisphosphate nucleotidase 1		2	4	3	**0.69**
121	Akr1c9 P23457: 3-alpha-hydroxysteroid dehydrogenase		4	4	3	**0.69**
122	Selenbp1 Q8VIF7: Selenium-binding protein 1		16	34	26	**0.69**
123	Eif4h Q5XI72: Eukaryotic translation initiation factor 4H		6	10	9	**0.69**
124	Atic O35567: Bifunctional purine biosynthesis protein PURH [Includes: Phosphoribosylamino ...		13	17	11	**0.69**
125	Hnrnph1 Q8VHV7: Heterogeneous nuclear ribonucleoprotein H		5	9	1	**0.70**
126	Tubb4b Q6P9T8: Tubulin beta-4B chain		12	57	3	**0.70**

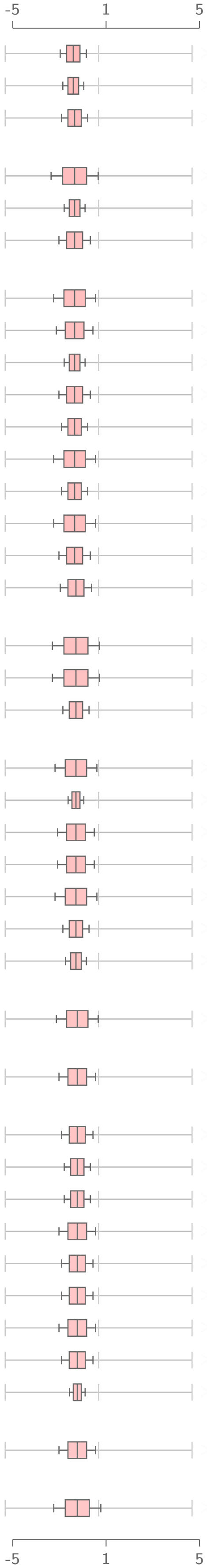

#	protein	group	peptides	spectra	quant	ratio
127	Aco1 Q63270: Cytoplasmic aconitate hydratase	8	14	12	**0.70**
128	Pgk1 P16617: Phosphoglycerate kinase 1	13	25	23	**0.70**
129	Tpi1 P48500: Triosephosphate isomerase	14	39	33	**0.70**
130	Dak Q4KLZ6: Bifunctional ATP-dependent dihydroxyacetone kinase/FAD-AMP lyase	2	2	2	**0.70**
131	Asns P49088: Asparagine synthetase [glutamine-hydrolyzing]	12	26	19	**0.70**
132	Efhd2 Q4FZY0: EF-hand domain-containing protein D2	3	7	5	**0.70**
133	Cmpk1 Q4KM73: UMP-CMP kinase	8	20	15	**0.70**
134	Clic4 Q9Z0W7: Chloride intracellular channel protein 4	3	3	3	**0.71**
135	Pfn1 P62963: Profilin-1	9	23	18	**0.71**
136	Anxa5 P14668: Annexin A5	12	26	21	**0.71**
137	Pkm P11980: Pyruvate kinase PKM	14	20	17	**0.71**
138	Ak2 P29410: Adenylate kinase 2, mitochondrial	8	9	9	**0.71**
139	Hsp90aa1 P82995: Heat shock protein HSP 90-alpha	23	49	16	**0.71**
140	Adh5 P12711: Alcohol dehydrogenase class-3	11	14	11	**0.71**
141	Ppia P10111: Peptidyl-prolyl cis-trans isomerase A	9	24	17	**0.71**
142	Gpi Q6P6V0: Glucose-6-phosphate isomerase	10	21	15	**0.71**
143	Khsrp Q99PF5: Far upstream element-binding protein 2	3	3	3	**0.71**
144	Ube2d3 P61078: Ubiquitin-conjugating enzyme E2 D3	2	2	2	**0.72**
145	Akr7a2 Q8CG45: Aflatoxin B1 aldehyde reductase member 2	5	9	7	**0.72**
146	Atp5b P10719: ATP synthase subunit beta, mitochondrial	9	17	13	**0.72**
147	Park7 O88767: Protein DJ-1	12	29	23	**0.72**
148	Fdps P05369: Farnesyl pyrophosphate synthase	3	5	5	**0.72**
149	Cpped1 Q66H71: Serine/threonine-protein phosphatase CPPED1	3	3	3	**0.72**
150	Yars Q4KM49: Tyrosine--tRNA ligase, cytoplasmic	16	22	15	**0.72**
151	Anxa1 P07150: Annexin A1	15	22	17	**0.72**
152	Q00715: Histone H2B type 1	2	2	2	**0.72**
153	Skp1 Q6PEC4: S-phase kinase-associated protein 1	2	2	2	**0.72**
154	Ahcy P10760: Adenosylhomocysteinase	21	71	63	**0.72**
155	Vcp P46462: Transitional endoplasmic reticulum ATPase	25	45	41	**0.72**
156	Wars Q6P7B0: Tryptophan--tRNA ligase, cytoplasmic	15	19	16	**0.72**
157	Uba1 Q5U300: Ubiquitin-like modifier-activating enzyme 1	26	51	45	**0.72**
158	Nmral1 P86172: NmrA-like family domain-containing protein 1	2	2	2	**0.72**
159	Myh9 Q62812: Myosin-9	21	28	15	**0.73**
160	Gamt P10868: Guanidinoacetate N-methyltransferase	8	15	12	**0.73**
161	Nedd4 Q62940: E3 ubiquitin-protein ligase NEDD4	3	4	3	**0.73**
162	Mat2b Q5U2R0: Methionine adenosyltransferase 2 subunit beta	3	3	3	**0.73**
163	Taldo1 Q9EQS0: Transaldolase	13	28	18	**0.73**
164	Uba5 Q5M7A4: Ubiquitin-like modifier-activating enzyme 5	9	12	9	**0.73**
165	Calr P18418: Calreticulin	12	23	19	**0.73**
166	Mdh1 O88989: Malate dehydrogenase, cytoplasmic	9	29	26	**0.73**
167	Pls3 Q63598: Plastin-3	2	3	3	**0.73**

#	protein	group	peptides	spectra	quant	ratio
168	Pdxk O35331: Pyridoxal kinase		3	5	5	**0.73**
169	Actn4 Q9QXQ0: Alpha-actinin-4		21	33	13	**0.73**
170	Alad P06214: Delta-aminolevulinic acid dehydratase		4	6	5	**0.73**
171	G6pdx P05370: Glucose-6-phosphate 1-dehydrogenase		2	2	2	**0.74**
172	Blvra P46844: Biliverdin reductase A		3	3	3	**0.74**
173	Ak1 P39069: Adenylate kinase isoenzyme 1		6	11	8	**0.74**
174	Tars Q5XHY5: Threonine--tRNA ligase, cytoplasmic		14	29	21	**0.74**
175	Fbp1 P19112: Fructose-1,6-bisphosphatase 1		7	11	5	**0.74**
176	Gnmt P13255: Glycine N-methyltransferase		6	20	16	**0.75**
177	Rhoa P61589: Transforming protein RhoA	1/2	7	9	4	**0.75**
178	Isyna1 Q6AYK3: Inositol-3-phosphate synthase 1		6	12	9	**0.75**
179	Cth P18757: Cystathionine gamma-lyase		12	54	42	**0.75**
180	Tbca Q6PEC1: Tubulin-specific chaperone A		4	4	3	**0.75**
181	Dstn Q7M0E3: Destrin		11	22	15	**0.75**
182	Fah P25093: Fumarylacetoacetase		5	7	5	**0.75**
183	Prkaca P27791: cAMP-dependent protein kinase catalytic subunit alpha		3	3	1	**0.75**
184	Nit1 Q7TQ94: Nitrilase homolog 1		5	9	7	**0.76**
185	Atp6v1b2 P62815: V-type proton ATPase subunit B, brain isoform		2	2	2	**0.76**
186	Rnh1 P29315: Ribonuclease inhibitor		20	79	63	**0.76**
187	Ywhaz P63102: 14-3-3 protein zeta/delta		13	37	20	**0.76**
188	Krt75 Q6IG05: Keratin, type II cytoskeletal 75		2	2	1	**0.76**
189	Gstp1 P04906: Glutathione S-transferase P		6	18	15	**0.76**
190	Hnrnpdl Q3SWU3: Heterogeneous nuclear ribonucleoprotein D-like		2	3	2	**0.76**
191	Prdx6 O35244: Peroxiredoxin-6		5	6	4	**0.76**
192	Mri1 Q5HZE4: Methylthioribose-1-phosphate isomerase		4	4	3	**0.76**
193	Phgdh O08651: D-3-phosphoglycerate dehydrogenase		11	24	19	**0.76**
194	Aldh1l1 P28037: Cytosolic 10-formyltetrahydrofolate dehydrogenase		29	49	38	**0.76**
195	Mylk2 P20689: Myosin light chain kinase 2, skeletal/cardiac muscle		3	3	2	**0.77**
196	Csde1 P18395: Cold shock domain-containing protein E1		2	2	2	**0.77**
197	Vcl P85972: Vinculin		7	10	9	**0.77**
198	Pnlip P27657: Pancreatic triacylglycerol lipase		22	203	156	**0.77**
199	Tagln2 Q5XFX0: Transgelin-2		8	19	16	**0.78**
200	Fbp2 Q9Z1N1: Fructose-1,6-bisphosphatase isozyme 2		10	18	12	**0.78**
201	Xdh P22985: Xanthine dehydrogenase/oxidase [Includes: Xanthine dehydrogenase		6	7	5	**0.78**
202	Gars Q5I0G4: Glycine--tRNA ligase		15	24	21	**0.78**
203	Hspa4 O88600: Heat shock 70 kDa protein 4		16	23	18	**0.78**
204	Got1 P13221: Aspartate aminotransferase, cytoplasmic		8	11	7	**0.78**
205	Strap Q5XIG8: Serine-threonine kinase receptor-associated protein		2	2	2	**0.78**
206	Ufc1 Q6BBI8: Ubiquitin-fold modifier-conjugating enzyme 1		3	6	4	**0.78**
207	Rnpep O09175: Aminopeptidase B		10	16	11	**0.79**

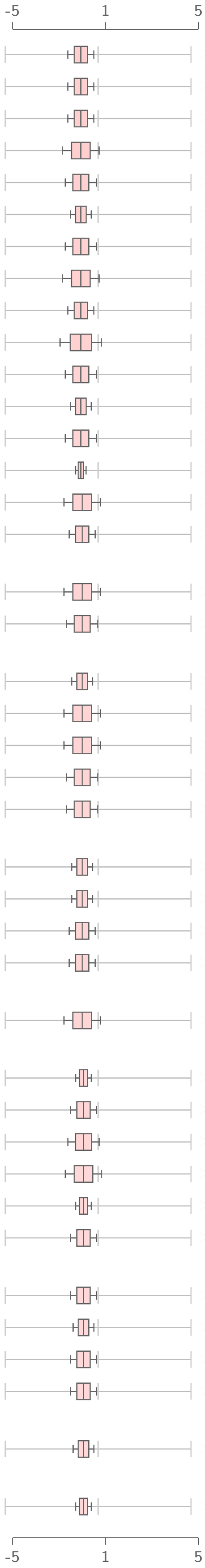

#	protein	group	peptides	spectra	quant	ratio
208	Hprt1 P27605: Hypoxanthine-guanine phosphoribosyltransferase		7	12	9	**0.79**
209	Pbld Q68G31: Phenazine biosynthesis-like domain-containing protein		5	10	7	**0.79**
210	Cndp2 Q6Q0N1: Cytosolic non-specific dipeptidase		5	5	3	**0.79**
211	Pebp1 P31044: Phosphatidylethanolamine-binding protein 1		7	13	9	**0.80**
212	Hsp90ab1 P34058: Heat shock protein HSP 90-beta		25	57	20	**0.80**
213	Scly Q68FT9: Selenocysteine lyase		2	2	2	**0.80**
214	Lypla2 Q9QYL8: Acyl-protein thioesterase 2		2	2	2	**0.80**
215	Eno1 P04764: Alpha-enolase	1/3	18	48	29	**0.80**
216	Sars Q6P799: Serine--tRNA ligase, cytoplasmic		10	21	18	**0.80**
217	Capzb Q5XI32: F-actin-capping protein subunit beta		6	8	7	**0.80**
218	Prkar2a P12368: cAMP-dependent protein kinase type II-alpha regulatory subunit		2	2	2	**0.80**
219	Syncrip Q7TP47: Heterogeneous nuclear ribonucleoprotein Q		3	4	3	**0.80**
220	Gmppa Q5XIC1: Mannose-1-phosphate guanyltransferase alpha		4	6	5	**0.81**
221	Actb P60711, Actg1 P63259: Actin, cytoplasmic 1, Actin, cytoplasmic 2	2/2	17	86	47	**0.81**
222	Ube2n Q9EQX9: Ubiquitin-conjugating enzyme E2 N		5	8	6	**0.81**
223	Atp5a1 P15999: ATP synthase subunit alpha, mitochondrial		3	3	3	**0.82**
224	Pgls P85971: 6-phosphogluconolactonase		6	13	10	**0.82**
225	Ide P35559: Insulin-degrading enzyme		6	6	5	**0.82**
226	Qdpr P11348: Dihydropteridine reductase		4	5	4	**0.82**
227	Txnl1 Q920J4: Thioredoxin-like protein 1		5	9	7	**0.82**
228	Oplah P97608: 5-oxoprolinase		6	6	5	**0.83**
229	Gda Q9WTT6: Guanine deaminase		13	17	13	**0.83**
230	Uso1 P41542: General vesicular transport factor p115		13	18	13	**0.83**
231	Thop1 P24155: Thimet oligopeptidase		6	8	7	**0.83**
232	Glo1 Q6P7Q4: Lactoylglutathione lyase		6	11	8	**0.83**
233	Pgm1 P38652: Phosphoglucomutase-1		8	11	10	**0.83**
234	Ciapin1 Q5XID1: Anamorsin		2	2	2	**0.86**
235	Eef2 P05197: Elongation factor 2		32	93	85	**1.19**
236	Actr3 Q4V7C7: Actin-related protein 3		5	11	9	**1.20**
237	P11517: Hemoglobin subunit beta-2		10	36	16	**1.29**
238	Arpc1b O88656: Actin-related protein 2/3 complex subunit 1B		2	2	2	**1.32**
239	Rab1A Q6NYB7: Ras-related protein Rab-1A	1/7	4	7	2	**1.35**
240	Rab11a P62494, Rab11b O35509: Ras-related protein Rab-11A, Ras-related protein Rab-11B	2/2	2	2	2	**1.35**
241	Hnrnpk P61980: Heterogeneous nuclear ribonucleoprotein K		14	28	24	**1.36**
242	Rab2a P05712: Ras-related protein Rab-2A		3	4	3	**1.48**
243	Prdx1 Q63716: Peroxiredoxin-1		9	30	15	**1.55**
244	Ptbp1 Q00438: Polypyrimidine tract-binding protein 1	1/2	3	7	3	**1.71**
245	Rab14 P61107: Ras-related protein Rab-14		2	2	1	**1.78**

#	protein	group	peptides	spectra	quant	ratio
246	Rab3a P63012, Rab3b Q63941, Rab3c P62824, ...: Ras-related protein Rab-3A, Ras-related protein Rab-3B, Ras-related protein Rab-3C, GTP-binding protein Rab-3D	4/4	2	2	1	**1.80**
247	Echdc1 Q6AYG5: Ethylmalonyl-CoA decarboxylase		2	2	2	**1.80**
248	Tpt1 P63029: Translationally-controlled tumor protein		5	9	7	**1.87**
249	Serping1 Q6P734: Plasma protease C1 inhibitor		5	6	6	**1.97**
250	Copb1 P23514: Coatomer subunit beta		5	6	5	**2.13**
251	Ctrc P55091: Chymotrypsin-C		3	5	4	**2.17**
252	Krt8 Q10758: Keratin, type II cytoskeletal 8		11	13	9	**2.32**
253	Ctsd P24268: Cathepsin D		2	2	2	**2.33**
254	Copg1 Q4AEF8: Coatomer subunit gamma-1	1/2	6	8	3	**2.38**
255	Serpina6 P31211: Corticosteroid-binding globulin		3	3	3	**2.45**
256	Ttr P02767: Transthyretin		5	11	9	**2.52**
257	Serpina3m Q63556: Serine protease inhibitor A3M		5	5	3	**2.57**
258	F2 P18292: Prothrombin		8	10	9	**2.65**
259	Mug1 Q03626: Murinoglobulin-1		30	101	21	**2.92**
260	Hpx P20059: Hemopexin		21	61	57	**2.95**
261	P02761: Major urinary protein		5	5	3	**2.95**
262	Map1 P01048: T-kininogen 1		8	12	5	**2.97**
263	Glud1 P10860: Glutamate dehydrogenase 1, mitochondrial		2	2	2	**3.03**
264	Fetub Q9QX79: Fetuin-B		9	17	14	**3.08**
265	Fgg P02680: Fibrinogen gamma chain		4	9	9	**3.12**
266	Itih3 Q63416: Inter-alpha-trypsin inhibitor heavy chain H3		8	9	9	**3.19**
267	Cp P13635: Ceruloplasmin		18	34	30	**3.30**
268	Afm P36953: Afamin		14	29	28	**3.35**
269	C9 Q62930: Complement component C9		2	2	2	**3.39**
270	Serpina1 P17475: Alpha-1-antiproteinase		13	31	28	**3.43**
271	Serpina3l P05544: Serine protease inhibitor A3L	1/2	18	38	12	**3.60**
272	Tf P12346: Serotransferrin		31	115	106	**3.85**
273	Rbp4 P04916: Retinol-binding protein 4		2	2	2	**3.95**
274	Ahsg P24090: Alpha-2-HS-glycoprotein		8	23	19	**4.07**
275	Igg-2a P20760: Ig gamma-2A chain C region		7	10	5	**4.07**
276	Serpina3k P05545: Serine protease inhibitor A3K		19	42	22	**4.16**
277	Agt P01015: Angiotensinogen		3	4	3	**4.16**
278	Igh-1a P20761: Ig gamma-2B chain C region		3	11	10	**4.18**
279	Clu P05371: Clusterin		4	4	3	**4.25**
280	P01835: Ig kappa chain C region, B allele		2	5	3	**4.39**
281	A1m Q63041: Alpha-1-macroglobulin		29	74	69	**4.47**
282	Fgb P14480: Fibrinogen beta chain		7	11	9	**4.49**
283	Gc P04276: Vitamin D-binding protein		13	25	24	**4.72**
284	Alb P02770: Serum albumin		44	418	397	**4.75**
285	C3 P01026: Complement C3 [Cleaved into: Complement C3 beta chain; C3-beta-c		46	89	86	**4.94**
286	A1i3 P14046: Alpha-1-inhibitor 3		32	115	41	**4.95**
287	P08932: T-kininogen 2		7	10	2	**4.97**
288	Hp P06866: Haptoglobin		14	37	35	**5.02**
289	Kng1 P08934: Kininogen-1 [Cleaved into: Kininogen-1 heavy chain; Bradykinin; Kininogen-1 ...		6	10	3	**5.02**

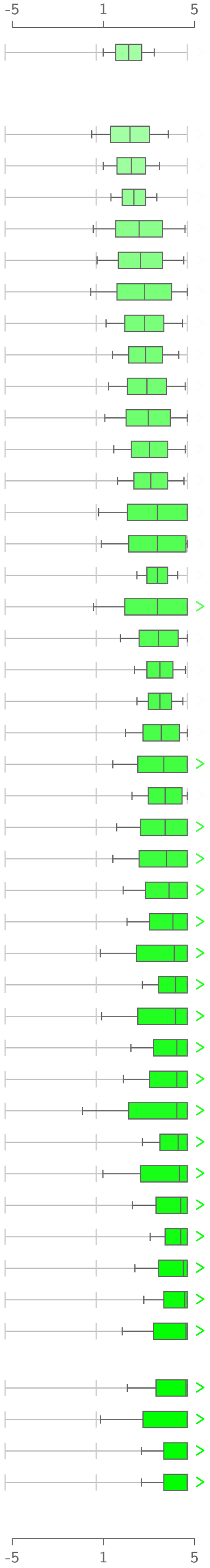

#	protein	group	peptides	spectra	quant	ratio
290	Fga P06399: Fibrinogen alpha chain [Cleaved into: Fibrinopeptide A; Fibrinogen alpha chain]		12	16	16	**5.26**
291	Ces1c P10959: Carboxylesterase 1C	1/2	12	25	18	**5.28**
292	Cfi Q9WUW3: Complement factor I		2	2	2	**5.28**
293	Ambp Q64240: Protein AMBP [Cleaved into: Alpha-1-microglobulin; Inter-alpha-trypsin inhib ...		2	3	3	**5.37**
294	Apoe P02650: Apolipoprotein E		8	12	10	**5.66**
295	Crp P48199: C-reactive protein		4	18	16	**6.12**
296	Orm1 P02764: Alpha-1-acid glycoprotein		3	4	3	**6.85**
297	Apoa1 P04639: Apolipoprotein A-I		13	39	38	**6.92**
298	P20759: Ig gamma-1 chain C region		3	4	1	**8.96**

2 Protein Relative Quantitation X / Y

Number of quantified proteins:

2.1 Quantified Proteins

#	protein	group	peptides	spectra	quant	ratio
**1**	A1i3 P14046: Alpha-1-inhibitor 3		32	115	41	**4.95***
**2**	A1m Q63041: Alpha-1-macroglobulin		29	74	69	**4.47***
**3**	Aars P50475: Alanine--tRNA ligase, cytoplasmic		15	24	16	**0.69***
**4**	Abcf1 Q6MG08: ATP-binding cassette sub-family F member 1		2	2	2	**0.81**
**5**	Abhd14b Q6DGG1: Alpha/beta hydrolase domain-containing protein 14B		4	9	7	**0.83**
**6**	Acaa2 P13437: 3-ketoacyl-CoA thiolase, mitochondrial		3	3	3	**0.48***
**7**	Acadl P15650: Long-chain specific acyl-CoA dehydrogenase, mitochondrial		2	3	3	**0.54***
**8**	Acadm P08503: Medium-chain specific acyl-CoA dehydrogenase, mitochondrial		2	2	2	**0.44***
**9**	Acat1 P17764: Acetyl-CoA acetyltransferase, mitochondrial		12	37	32	**0.43***
**10**	Acat2 Q5XI22: Acetyl-CoA acetyltransferase, cytosolic		2	2	2	**0.74**
**11**	Acly P16638: ATP-citrate synthase		16	21	17	**0.78**
**12**	Aco1 Q63270: Cytoplasmic aconitate hydratase		8	14	12	**0.70***
**13**	Aco2 Q9ER34: Aconitate hydratase, mitochondrial		19	33	29	**0.52***
**14**	Acta1 P68136, Acta2 P62738, Actc1 P68035, ...: Actin, alpha skeletal muscle, Actin, aortic smooth muscle, Actin, alpha cardiac muscle 1, Actin, gamma-enteric smooth muscle	4/4	10	35	2	**2.10**
**15**	Actb P60711, Actg1 P63259: Actin, cytoplasmic 1, Actin, cytoplasmic 2	2/2	17	86	47	**0.81***
**16**	Actn1 Q9Z1P2: Alpha-actinin-1		15	26	7	**0.84**

#	protein	group	peptides	spectra	quant	ratio
17	Actn4 Q9QXQ0: Alpha-actinin-4		21	33	13	**0.73***
18	Actr1a P85515: Alpha-centractin		3	3	3	**0.52***
19	Actr2 Q5M7U6: Actin-related protein 2		2	2	2	**1.06**
20	Actr3 Q4V7C7: Actin-related protein 3		5	11	9	**1.20***
21	Acy1a Q6AYS7: Aminoacylase-1A	1/2	11	23	18	**0.98**
22	Acyp2 P35745: Acylphosphatase-2		2	2	2	**0.84**
23	Adh5 P12711: Alcohol dehydrogenase class-3		11	14	11	**0.71***
24	Adi1 Q562C9: 1,2-dihydroxy-3-keto-5-methylthiopentene dioxygenase		3	3	3	**0.99**
25	Adk Q64640: Adenosine kinase		10	23	19	**0.77**
26	Afm P36953: Afamin		14	29	28	**3.35***
27	Agt P01015: Angiotensinogen		3	4	3	**4.16***
28	Ahcy P10760: Adenosylhomocysteinase		21	71	63	**0.72***
29	Ahsg P24090: Alpha-2-HS-glycoprotein		8	23	19	**4.07***
30	Ak1 P39069: Adenylate kinase isoenzyme 1		6	11	8	**0.74***
31	Ak2 P29410: Adenylate kinase 2, mitochondrial		8	9	9	**0.71***
32	Ak3 P29411: GTP:AMP phosphotransferase AK3, mitochondrial		4	7	4	**0.63**
33	Akr1a1 P51635: Alcohol dehydrogenase [NADP(+)]		8	16	15	**0.68***
34	Akr1b1 P07943: Aldose reductase		14	24	20	**0.63***
35	Akr1c9 P23457: 3-alpha-hydroxysteroid dehydrogenase		4	4	3	**0.69***
36	Akr7a2 Q8CG45: Aflatoxin B1 aldehyde reductase member 2		5	9	7	**0.72***
37	Akt1 P47196: RAC-alpha serine/threonine-protein kinase		2	2	2	**0.74**
38	Alad P06214: Delta-aminolevulinic acid dehydratase		4	6	5	**0.73***
39	Alb P02770: Serum albumin		44	418	397	**4.75***
40	Aldh16a1 Q3T1L0: Aldehyde dehydrogenase family 16 member A1		2	3	3	**0.62***
41	Aldh1a1 P51647: Retinal dehydrogenase 1	1/2	15	43	20	**0.84**
42	Aldh1l1 P28037: Cytosolic 10-formyltetrahydrofolate dehydrogenase		29	49	38	**0.76***
43	Aldh2 P11884: Aldehyde dehydrogenase, mitochondrial		13	23	17	**0.38***
44	Aldh6a1 Q02253: Methylmalonate-semialdehyde dehydrogenase [acylating], mitochondrial		16	31	27	**0.35***
45	Aldh9a1 Q9JLJ3: 4-trimethylaminobutyraldehyde dehydrogenase		12	22	19	**0.68***
46	Aldoa P05065: Fructose-bisphosphate aldolase A		9	10	7	**1.10**
47	Aldob P00884: Fructose-bisphosphate aldolase B		9	14	12	**0.82**
48	Ambp Q64240: Protein AMBP [Cleaved into: Alpha-1-microglobulin; Inter-alpha-trypsin inhib ...		2	3	3	**5.37***
49	Amy2 P00689: Pancreatic alpha-amylase		27	416	308	**0.59***
50	Anp32a P49911: Acidic leucine-rich nuclear phosphoprotein 32 family member A		3	5	3	**0.92**

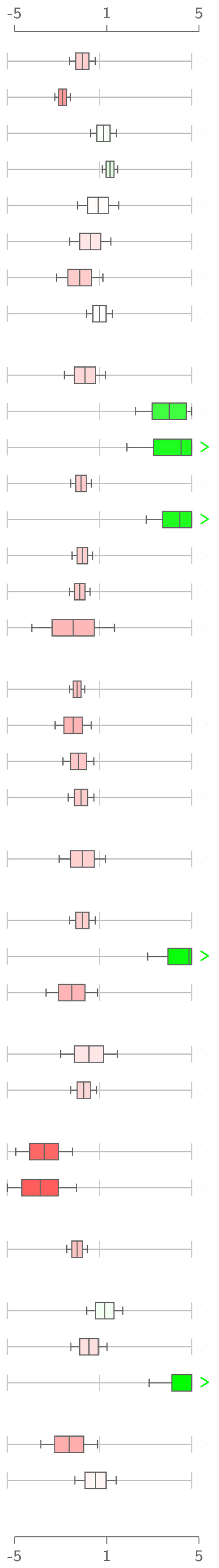

#	protein	group	peptides	spectra	quant	ratio
**51**	Anp32e Q5XIE0: Acidic leucine-rich nuclear phosphoprotein 32 family member E		2	2	2	**0.88**
**52**	Anxa1 P07150: Annexin A1		15	22	17	**0.72***
**53**	Anxa3 P14669: Annexin A3		2	3	3	**0.45***
**54**	Anxa4 P55260: Annexin A4		6	7	5	**0.61***
**55**	Anxa5 P14668: Annexin A5		12	26	21	**0.71***
**56**	Anxa6 P48037: Annexin A6		27	46	38	**0.50***
**57**	Apeh P13676: Acylamino-acid-releasing enzyme		2	2	2	**1.02**
**58**	Apoa1 P04639: Apolipoprotein A-I		13	39	38	**6.92***
**59**	Apoa1bp B0BNM1: NAD(P)H-hydrate epimerase		2	4	3	**0.46***
**60**	Apoa4 P02651: Apolipoprotein A-IV		4	4	3	**1.52**
**61**	Apoe P02650: Apolipoprotein E		8	12	10	**5.66***
**62**	Aprt P36972: Adenine phosphoribosyltransferase		9	20	15	**0.70**
**63**	Arcn1 Q66H80: Coatomer subunit delta		5	8	7	**1.77**
**64**	Arf1 P84079, Arf3 P61206: ADP-ribosylation factor 1, ADP-ribosylation factor 3	2/3	6	18	1	**0.91**
**65**	Arf4 P61751: ADP-ribosylation factor 4		5	14	3	**0.83**
**67**	Arhgdia Q5XI73: Rho GDP-dissociation inhibitor 1		6	24	18	**0.82**
**68**	Arl1 P61212: ADP-ribosylation factor-like protein 1		2	5	5	**1.25**
**69**	Arpc1b O88656: Actin-related protein 2/3 complex subunit 1B		2	2	2	**1.32***
**70**	Arpc2 P85970: Actin-related protein 2/3 complex subunit 2		2	2	2	**0.83**
**71**	As3mt Q8VHT6: Arsenite methyltransferase		2	2	2	**0.84**
**72**	Asns P49088: Asparagine synthetase [glutamine-hydrolyzing]		12	26	19	**0.70***
**73**	Atg3 Q6AZ50: Ubiquitin-like-conjugating enzyme ATG3		2	2	2	**0.81**
**74**	Atg7 Q641Y5: Ubiquitin-like modifier-activating enzyme ATG7		2	3	3	**0.56***
**75**	Atic O35567: Bifunctional purine biosynthesis protein PURH [Includes: Phosphoribosylamino . . .		13	17	11	**0.69***
**76**	Atp5a1 P15999: ATP synthase subunit alpha, mitochondrial		3	3	3	**0.82***
**77**	Atp5b P10719: ATP synthase subunit beta, mitochondrial		9	17	13	**0.72***
**78**	Atp6v1b2 P62815: V-type proton ATPase subunit B, brain isoform		2	2	2	**0.76***
**79**	Bcat2 O35854: Branched-chain-amino-acid aminotransferase, mitochondrial		11	30	26	**0.50***
**80**	Bdh2 D4A1J4: 3-hydroxybutyrate dehydrogenase type 2		5	7	5	**0.59***
**81**	Blvra P46844: Biliverdin reductase A		3	3	3	**0.74***
**82**	Bpnt1 Q9Z1N4: 3'(2'),5'-bisphosphate nucleotidase 1		2	4	3	**0.69***
**83**	C1qbp O35796: Complement component 1 Q subcomponent-binding protein, mitochondrial		3	6	5	**0.58***

#	protein	group	peptides	spectra	quant	ratio
84	C3 P01026: Complement C3 [Cleaved into: Complement C3 beta chain; C3-beta-c		46	89	86	**4.94***
85	C4 P08649: Complement C4 [Cleaved into: Complement C4 beta chain; Complement C4 alpha c . . .		3	3	3	**1.42**
86	C9 Q62930: Complement component C9		2	2	2	**3.39***
87	Ca1 B0BNN3: Carbonic anhydrase 1		5	10	7	**1.08**
88	Ca2 P27139: Carbonic anhydrase 2		3	4	3	**0.89**
89	Ca3 P14141: Carbonic anhydrase 3		7	11	10	**0.59***
90	Calm1 P62161: Calmodulin	1/2	4	6	2	**0.58***
91	Calr P18418: Calreticulin		12	23	19	**0.73***
92	Cand1 P97536: Cullin-associated NEDD8-dissociated protein 1		8	9	7	**0.95**
93	Cap1 Q08163: Adenylyl cyclase-associated protein 1		4	7	5	**0.64***
94	Capn2 Q07009: Calpain-2 catalytic subunit		5	6	5	**0.47***
95	Capza1 B2GUZ5: F-actin-capping protein subunit alpha-1		2	3	1	**0.95**
96	Capza2 Q3T1K5: F-actin-capping protein subunit alpha-2		4	6	3	**0.93**
97	Capzb Q5XI32: F-actin-capping protein subunit beta		6	8	7	**0.80***
98	Casp6 O35397: Caspase-6		3	3	3	**0.77**
99	Cast P27321: Calpastatin		8	11	8	**0.77**
100	Cat P04762: Catalase		2	2	2	**1.03**
101	Cbr1 P47727: Carbonyl reductase [NADPH] 1		6	8	6	**0.81**
102	Cbs P32232: Cystathionine beta-synthase		10	13	9	**0.81**
103	Ccbl1 Q08415: Kynurenine--oxoglutarate transaminase 1, mitochondrial		3	3	3	**0.73**
104	Ccbl2 Q58FK9: Kynurenine--oxoglutarate transaminase 3		2	2	2	**0.63**
105	Cct5 Q68FQ0: T-complex protein 1 subunit epsilon		4	4	3	**0.85**
106	Cd2ap F1LRS8: CD2-associated protein		2	2	2	**0.89**
107	Cdc42 Q8CFN2: Cell division control protein 42 homolog		6	6	5	**0.65***
108	Cdv3 Q5XIM5: Protein CDV3 homolog		4	8	6	**0.79**
109	Cel P07882: Bile salt-activated lipase		17	60	44	**0.80**
110	Cela1 P00773: Chymotrypsin-like elastase family member 1		2	11	9	**0.77**
111	Cela2a P00774: Chymotrypsin-like elastase family member 2A		9	234	164	**0.65***
112	Ces1c P10959: Carboxylesterase 1C	1/2	12	25	18	**5.28***
113	Cfi Q9WUW3: Complement factor I		2	2	2	**5.28***
114	Cfl1 P45592: Cofilin-1		9	18	13	**0.73**
115	Ciapin1 Q5XID1: Anamorsin		2	2	2	**0.86***
116	Ckb P07335: Creatine kinase B-type		6	8	6	**1.08**
117	Clic4 Q9Z0W7: Chloride intracellular channel protein 4		3	3	3	**0.71***
118	Cltc P11442: Clathrin heavy chain 1		11	14	12	**1.15**

-5 1 5

#	protein	group	peptides	spectra	quant	ratio
**119**	Clu P05371: Clusterin		4	4	3	**4.25***
**120**	Cmbl Q7TP52: Carboxymethylenebutenolidase homolog		2	2	2	**0.82**
**121**	Cmpk1 Q4KM73: UMP-CMP kinase		8	20	15	**0.70***
**122**	Cnbp P62634: Cellular nucleic acid-binding protein		8	17	14	**0.79**
**123**	Cndp2 Q6Q0N1: Cytosolic non-specific dipeptidase		5	5	3	**0.79***
**124**	Copb1 P23514: Coatomer subunit beta		5	6	5	**2.13***
**125**	Copb2 O35142: Coatomer subunit beta’		6	6	5	**1.55**
**126**	Copg1 Q4AEF8: Coatomer subunit gamma-1	1/2	6	8	3	**2.38***
**127**	Cops2 P61203: COP9 signalosome complex subunit 2		3	3	3	**0.82**
**128**	Cops4 Q68FS2: COP9 signalosome complex subunit 4		2	2	2	**0.84**
**129**	Cotl1 B0BNA5: Coactosin-like protein		2	2	2	**0.62**
**130**	Cp P13635: Ceruloplasmin		18	34	30	**3.30***
**131**	Cpa1 P00731: Carboxypeptidase A1		19	172	147	**0.66***
**132**	Cpa2 P19222: Carboxypeptidase A2		15	128	123	**0.62***
**133**	Cpb1 P19223: Carboxypeptidase B		15	171	121	**0.67***
**134**	Cpped1 Q66H71: Serine/threonine-protein phosphatase CPPED1		3	3	3	**0.72***
**135**	Cpq Q6IRK9: Carboxypeptidase Q		2	2	2	**0.57***
**136**	Crp P48199: C-reactive protein		4	18	16	**6.12***
**137**	Cs Q8VHF5: Citrate synthase, mitochondrial		4	4	3	**0.62**
**138**	Csde1 P18395: Cold shock domain-containing protein E1		2	2	2	**0.77***
**139**	Ctbp1 Q9Z2F5: C-terminal-binding protein 1		2	2	2	**0.51***
**140**	Cth P18757: Cystathionine gamma-lyase		12	54	42	**0.75***
**141**	Ctrb1 P07338: Chymotrypsinogen B		10	99	77	**0.65***
**142**	Ctrc P55091: Chymotrypsin-C		3	5	4	**2.17***
**143**	Ctsb P00787: Cathepsin B		2	2	2	**0.68***
**144**	Ctsd P24268: Cathepsin D		2	2	2	**2.33***
**145**	Cul5 Q9JJ31: Cullin-5		3	4	3	**0.79**
**146**	Cycs P62898: Cytochrome c, somatic		3	6	4	**0.99**
**147**	Dak Q4KLZ6: Bifunctional ATP-dependent dihydroxyacetone kinase/FAD-AMP lyase		2	2	2	**0.70***
**148**	Dars P15178: Aspartate--tRNA ligase, cytoplasmic		7	8	6	**0.91**
**149**	Dbi P11030: Acyl-CoA-binding protein		5	20	16	**0.67***
**150**	Dcps Q8K4F7: m7GpppX diphosphatase		3	3	3	**0.65***
**151**	Dcxr Q920P0: L-xylulose reductase		2	2	2	**0.67***
**152**	Ddah1 O08557: N(G),N(G)-dimethylarginine dimethylaminohydrolase 1		14	33	23	**0.83**
**153**	Ddb1 Q9ESW0: DNA damage-binding protein 1		6	7	5	**0.90**
**154**	Ddt P80254: D-dopachrome decarboxylase		3	6	5	**0.77**
**155**	Dhtkd1 Q4KLP0: Probable 2-oxoglutarate dehydrogenase E1 component DHKTD1, mitochondrial		3	4	3	**0.52***
**156**	Dld Q6P6R2: Dihydrolipoyl dehydrogenase, mitochondrial		9	16	12	**0.54***

#	protein	group	peptides	spectra	quant	ratio
**157**	Dpp3 O55096: Dipeptidyl peptidase 3		8	11	9	**0.88**
**158**	Dpysl2 P47942: Dihydropyrimidinase-related protein 2		3	5	3	**0.91**
**159**	Dstn Q7M0E3: Destrin		11	22	15	**0.75***
**160**	Echdc1 Q6AYG5: Ethylmalonyl-CoA decarboxylase		2	2	2	**1.80***
**161**	Echdc3 Q3MIE0: Enoyl-CoA hydratase domain-containing protein 3, mitochondrial		3	3	3	**0.44***
**162**	Echs1 P14604: Enoyl-CoA hydratase, mitochondrial		6	8	7	**0.77**
**163**	Eci1 P23965: Enoyl-CoA delta isomerase 1, mitochondrial		3	3	3	**0.66***
**164**	Eef1a1 P62630: Elongation factor 1-alpha 1	1/2	15	39	22	**1.44**
**165**	Eef1d Q68FR9: Elongation factor 1-delta		4	16	12	**0.82**
**166**	Eef1g Q68FR6: Elongation factor 1-gamma		10	12	9	**1.10**
**167**	Eef2 P05197: Elongation factor 2		32	93	85	**1.19***
**168**	Efhd2 Q4FZY0: EF-hand domain-containing protein D2		3	7	5	**0.70***
**169**	Eif2s3 P81795: Eukaryotic translation initiation factor 2 subunit 3		2	2	2	**1.17**
**170**	Eif3g Q5RK09: Eukaryotic translation initiation factor 3 subunit G		3	3	2	**0.88**
**171**	Eif4a2 Q5RKI1: Eukaryotic initiation factor 4A-II	1/2	12	25	16	**1.00**
**172**	Eif4e P63074: Eukaryotic translation initiation factor 4E		3	3	3	**0.59***
**173**	Eif4h Q5XI72: Eukaryotic translation initiation factor 4H		6	10	9	**0.69***
**174**	Eif5 Q07205: Eukaryotic translation initiation factor 5		3	4	4	**0.97**
**175**	Eif5a Q3T1J1: Eukaryotic translation initiation factor 5A-1		4	9	6	**0.78**
**176**	Eif5b B2GUV7: Eukaryotic translation initiation factor 5B		2	2	2	**1.51**
**177**	Eno1 P04764: Alpha-enolase	1/3	18	48	29	**0.80***
**178**	Erap1 Q9JJ22: Endoplasmic reticulum aminopeptidase 1		2	2	2	**0.89**
**179**	Esd B0BNE5: S-formylglutathione hydrolase		6	22	22	**0.64***
**180**	Esrp1 B2RYD2: Epithelial splicing regulatory protein 1		2	3	3	**0.82**
**181**	Etfa P13803: Electron transfer flavoprotein subunit alpha, mitochondrial		10	19	14	**0.52***
**182**	Etfb Q68FU3: Electron transfer flavoprotein subunit beta		9	13	8	**0.30***
**183**	F2 P18292: Prothrombin		8	10	9	**2.65***
**184**	Fabp4 P70623: Fatty acid-binding protein, adipocyte		7	15	11	**0.69**
**185**	Fabp5 P55053: Fatty acid-binding protein, epidermal		4	4	3	**0.80**
**186**	Fah P25093: Fumarylacetoacetase		5	7	5	**0.75***
**187**	Fahd1 Q6AYQ8: Acylpyruvase FAHD1, mitochondrial		2	2	2	**0.73**
**188**	Farsa Q505J8: Phenylalanine--tRNA ligase alpha subunit		7	9	6	**0.72**
**189**	Fasn P12785: Fatty acid synthase		19	25	22	**0.83**
**190**	Fbp1 P19112: Fructose-1,6-bisphosphatase 1		7	11	5	**0.74***
**191**	Fbp2 Q9Z1N1: Fructose-1,6-bisphosphatase isozyme 2		10	18	12	**0.78***
**192**	Fdps P05369: Farnesyl pyrophosphate synthase		3	5	5	**0.72***

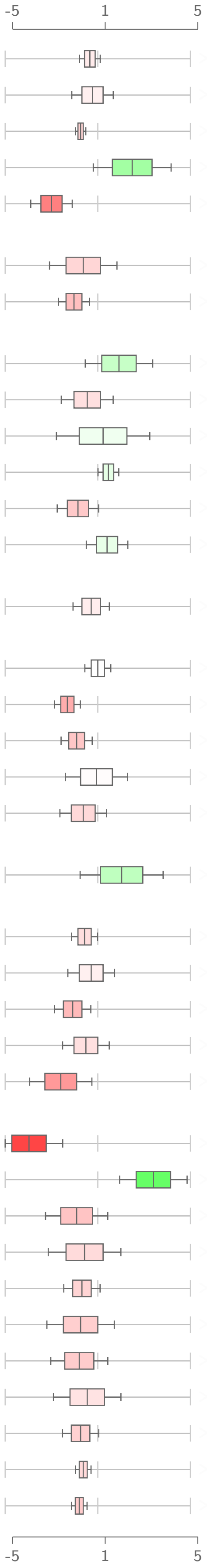

#	protein	group	peptides	spectra	quant	ratio
**193**	Fetub Q9QX79: Fetuin-B	9	17	14	**3.08***
**194**	Fga P06399: Fibrinogen alpha chain [Cleaved into: Fibrinopeptide A; Fibrinogen alpha chain]	12	16	16	**5.26***
**195**	Fgb P14480: Fibrinogen beta chain	7	11	9	**4.49***
**196**	Fgg P02680: Fibrinogen gamma chain	4	9	9	**3.12***
**197**	Fh P14408: Fumarate hydratase, mitochondrial	5	10	9	**0.52***
**198**	Fis1 P84817: Mitochondrial fission 1 protein	2	4	3	**0.77**
**199**	Fkbp1a Q62658: Peptidyl-prolyl cis-trans isomerase FKBP1A	2	3	3	**1.01**
**200**	Fkbp4 Q9QVC8: Peptidyl-prolyl cis-trans isomerase FKBP4	2	2	2	**0.89**
**201**	Ftl1 P02793: Ferritin light chain 1	3	3	3	**0.93**
**202**	Fubp1 Q32PX7: Far upstream element-binding protein 1	4	6	5	**0.81**
**203**	G6pdx P05370: Glucose-6-phosphate 1-dehydrogenase	2	2	2	**0.74***
**204**	Gamt P10868: Guanidinoacetate N-methyltransferase	8	15	12	**0.73***
**205**	Gapdh P04797: Glyceraldehyde-3-phosphate dehydrogenase	15	39	33	**0.81**
**206**	Gars Q5I0G4: Glycine--tRNA ligase	15	24	21	**0.78***
**207**	Gatm P50442: Glycine amidinotransferase, mitochondrial	21	43	38	**0.39***
**208**	Gc P04276: Vitamin D-binding protein	13	25	24	**4.72***
**209**	Gcg P06883: Glucagon [Cleaved into: Glicentin; Glicentin-related polypeptide	2	10	7	**0.88**
**210**	Gcsh Q5I0P2: Glycine cleavage system H protein, mitochondrial	2	2	2	**0.55***
**211**	Gda Q9WTT6: Guanine deaminase	13	17	13	**0.83***
**212**	Gdi1 P50398: Rab GDP dissociation inhibitor alpha	9	15	6	**0.68***
**213**	Gdi2 P50399: Rab GDP dissociation inhibitor beta	6	16	7	**1.17**
**214**	Ggh Q62867: Gamma-glutamyl hydrolase	2	2	2	**0.83**
**215**	Glo1 Q6P7Q4: Lactoylglutathione lyase	6	11	8	**0.83***
**216**	Glrx3 Q9JLZ1: Glutaredoxin-3	4	8	6	**0.63***
**217**	Glud1 P10860: Glutamate dehydrogenase 1, mitochondrial	2	2	2	**3.03***
**218**	Gmfb Q63228: Glia maturation factor beta	2	2	2	**0.90**
**219**	Gmppa Q5XIC1: Mannose-1-phosphate guanyltransferase alpha	4	6	5	**0.81***
**220**	Gnb2l1 P63245: Guanine nucleotide-binding protein subunit beta-2-like 1	2	5	3	**1.09**
**221**	Gnmt P13255: Glycine N-methyltransferase	6	20	16	**0.75***
**222**	Got1 P13221: Aspartate aminotransferase, cytoplasmic	8	11	7	**0.78***
**223**	Got2 P00507: Aspartate aminotransferase, mitochondrial	6	8	7	**0.45***
**224**	Gp2 P19218: Pancreatic secretory granule membrane major glycoprotein GP2	8	17	13	**1.13**
**225**	Gpd1 O35077: Glycerol-3-phosphate dehydrogenase [NAD(+)], cytoplasmic	5	13	12	**0.87**

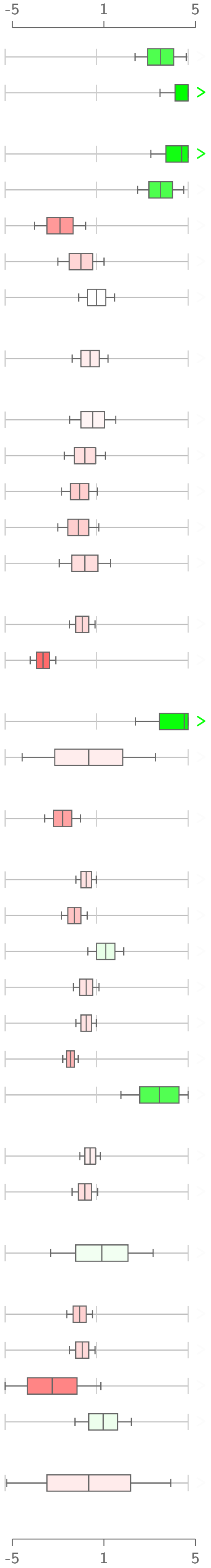

#	protein	group	peptides	spectra	quant	ratio
**226**	Gpi Q6P6V0: Glucose-6-phosphate isomerase		10	21	15	**0.71***
**227**	Gpt P25409: Alanine aminotransferase 1		4	6	4	**0.93**
**228**	Gpx3 P23764: Glutathione peroxidase 3		2	4	3	**1.59**
**229**	Gsn Q68FP1: Gelsolin		6	8	7	**1.45**
**230**	Gsr P70619: Glutathione reductase		2	3	3	**0.66***
**231**	Gsta3 P04904: Glutathione S-transferase alpha-3	1/3	4	4	2	**0.58**
**232**	Gsta4 P14942: Glutathione S-transferase alpha-4		5	7	7	**1.01**
**233**	Gsta5 P46418: Glutathione S-transferase alpha-5		2	2	1	**0.54***
**234**	Gstm1 P04905: Glutathione S-transferase Mu 1		10	15	6	**0.82**
**235**	Gstm2 P08010: Glutathione S-transferase Mu 2		19	47	23	**0.74**
**236**	Gstm3 P08009: Glutathione S-transferase Yb-3		9	21	2	**0.87**
**237**	Gstm5 Q9Z1B2: Glutathione S-transferase Mu 5		2	2	2	**0.73**
**238**	Gsto1 Q9Z339: Glutathione S-transferase omega-1		6	11	7	**0.83**
**239**	Gstp1 P04906: Glutathione S-transferase P		6	18	15	**0.76***
**240**	Gstt2 P30713: Glutathione S-transferase theta-2		6	10	7	**0.73**
**241**	Gstz1 P57113: Maleylacetoacetate isomerase		4	5	3	**0.69**
**242**	Hadh Q9WVK7: Hydroxyacyl-coenzyme A dehydrogenase, mitochondrial		7	12	12	**0.38***
**243**	Hagh O35952: Hydroxyacylglutathione hydrolase, mitochondrial		3	5	3	**0.66***
**244**	Hba1 P01946: Hemoglobin subunit alpha-1/2		8	48	37	**1.12**
**245**	Hbb P02091: Hemoglobin subunit beta-1		10	40	21	**1.05**
**246**	Hdgf Q8VHK7: Hepatoma-derived growth factor		2	2	2	**0.67**
**247**	Herc4 Q5PQN1: Probable E3 ubiquitin-protein ligase HERC4		2	2	2	**0.77**
**248**	Hibadh P29266: 3-hydroxyisobutyrate dehydrogenase, mitochondrial		3	5	4	**0.51***
**249**	Hint1 P62959: Histidine triad nucleotide-binding protein 1		2	8	6	**0.82**
**250**	Hmbs P19356: Porphobilinogen deaminase		5	5	4	**0.60***
**251**	Hmgcl P97519: Hydroxymethylglutaryl-CoA lyase, mitochondrial		4	4	3	**0.48***
**252**	Hmgcs2 P22791: Hydroxymethylglutaryl-CoA synthase, mitochondrial		3	3	3	**0.67***
**253**	Hnrnpa1 P04256: Heterogeneous nuclear ribonucleoprotein A1		4	5	4	**0.58***
**254**	Hnrnpa2b1 A7VJC2: Heterogeneous nuclear ribonucleoproteins A2/B1		14	27	18	**0.90**
**255**	Hnrnpa3 Q6URK4: Heterogeneous nuclear ribonucleoprotein A3		6	9	7	**0.87**
**256**	Hnrnpd Q9JJ54: Heterogeneous nuclear ribonucleoprotein D0		5	9	6	**0.61***
**257**	Hnrnpdl Q3SWU3: Heterogeneous nuclear ribonucleoprotein D-like		2	3	2	**0.76***

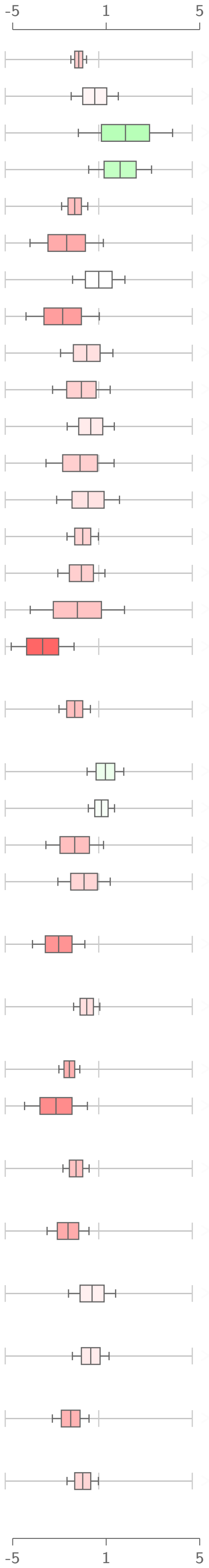

#	protein	group	peptides	spectra	quant	ratio
258	Hnrnpf Q794E4: Heterogeneous nuclear ribonucleoprotein F		7	10	7	**0.53***
259	Hnrnph1 Q8VHV7: Heterogeneous nuclear ribonucleoprotein H		5	9	1	**0.70***
260	Hnrnph2 Q6AY09: Heterogeneous nuclear ribonucleoprotein H2		6	9	2	**0.67***
261	Hnrnpk P61980: Heterogeneous nuclear ribonucleoprotein K		14	28	24	**1.36***
262	Hp P06866: Haptoglobin		14	37	35	**5.02***
263	Hprt1 P27605: Hypoxanthine-guanine phosphoribosyltransferase		7	12	9	**0.79***
264	Hpx P20059: Hemopexin		21	61	57	**2.95***
265	Hrsp12 P52759: Ribonuclease UK114		4	10	10	**0.66***
266	Hsd17b13 Q5M875: 17-beta-hydroxysteroid dehydrogenase 13		3	3	3	**1.40**
267	Hsp90aa1 P82995: Heat shock protein HSP 90-alpha		23	49	16	**0.71***
268	Hsp90ab1 P34058: Heat shock protein HSP 90-beta		25	57	20	**0.80***
269	Hsp90b1 Q66HD0: Endoplasmin		23	37	31	**0.87**
270	Hspa4 O88600: Heat shock 70 kDa protein 4		16	23	18	**0.78***
271	Hspa5 P06761: 78 kDa glucose-regulated protein		27	80	59	**0.80**
272	Hspa8 P63018: Heat shock cognate 71 kDa protein	1/4	27	60	30	**0.96**
273	Hspa9 P48721: Stress-70 protein, mitochondrial		8	10	8	**0.46***
274	Hspd1 P63039: 60 kDa heat shock protein, mitochondrial		17	29	21	**0.54***
275	Hspe1 P26772: 10 kDa heat shock protein, mitochondrial		6	12	8	**0.34***
276	Hsph1 Q66HA8: Heat shock protein 105 kDa		8	8	5	**0.78**
277	Hyou1 Q63617: Hypoxia up-regulated protein 1		10	14	11	**0.74**
278	Ide P35559: Insulin-degrading enzyme		6	6	5	**0.82***
279	Idh1 P41562: Isocitrate dehydrogenase [NADP] cytoplasmic		7	13	10	**0.67**
280	Idh2 P56574: Isocitrate dehydrogenase [NADP], mitochondrial		21	41	36	**0.34***
281	Igg-2a P20760: Ig gamma-2A chain C region		7	10	5	**4.07***
282	Igh-1a P20761: Ig gamma-2B chain C region		3	11	10	**4.18***
283	Impa1 P97697: Inositol monophosphatase 1		4	6	6	**0.73**
284	Isoc1 Q6I7R3: Isochorismatase domain-containing protein 1		2	3	3	**0.64***
285	Isyna1 Q6AYK3: Inositol-3-phosphate synthase 1		6	12	9	**0.75***
286	Itih3 Q63416: Inter-alpha-trypsin inhibitor heavy chain H3		8	9	9	**3.19***
287	Itpa D3ZW55: Inosine triphosphate pyrophosphatase		2	3	3	**0.96**
288	Ivd P12007: Isovaleryl-CoA dehydrogenase, mitochondrial		7	16	14	**0.52***
289	Khsrp Q99PF5: Far upstream element-binding protein 2		3	3	3	**0.71***
290	Kng1 P08934: Kininogen-1 [Cleaved into: Kininogen-1 heavy chain; Bradykinin; Kininogen-1 . . .		6	10	3	**5.02***

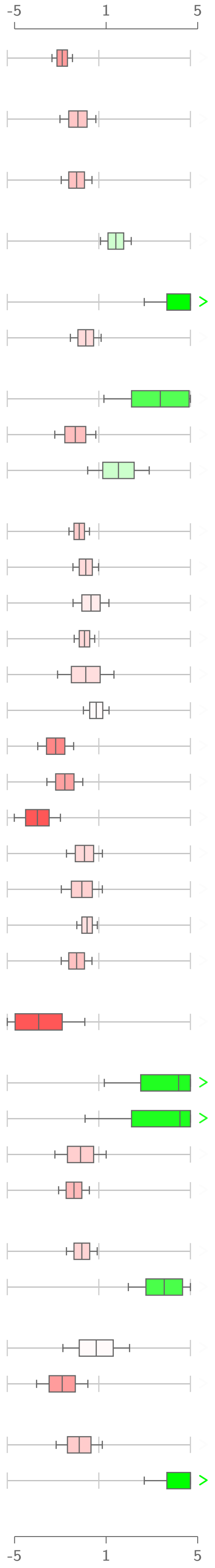

#	protein	group	peptides	spectra	quant	ratio
**291**	Kpnb1 P52296: Importin subunit beta-1		5	8	6	**1.08**
**292**	Krt18 Q5BJY9: Keratin, type I cytoskeletal 18		13	17	12	**2.06**
**293**	Krt75 Q6IG05: Keratin, type II cytoskeletal 75		2	2	1	**0.76***
**294**	Krt8 Q10758: Keratin, type II cytoskeletal 8		11	13	9	**2.32***
**295**	Lap3 Q68FS4: Cytosol aminopeptidase		12	16	14	**0.62***
**296**	Lasp1 Q99MZ8: LIM and SH3 domain protein 1		7	7	6	**1.07**
**297**	Ldha P04642: L-lactate dehydrogenase A chain		16	46	31	**0.70**
**298**	Ldhb P42123: L-lactate dehydrogenase B chain		5	12	3	**1.08**
**299**	Lgals1 P11762: Galectin-1		4	5	4	**0.96**
**300**	Lta4h P30349: Leukotriene A-4 hydrolase		9	15	12	**0.83**
**301**	Lum P51886: Lumican		7	11	8	**0.58***
**302**	Lypla2 Q9QYL8: Acyl-protein thioesterase 2		2	2	2	**0.80***
**303**	Manf P0C5H9: Mesencephalic astrocyte-derived neurotrophic factor		2	4	3	**0.83**
**304**	Map1 P01048: T-kininogen 1		8	12	5	**2.97***
**305**	Mapk10 P49187, Mapk9 P49186: Mitogen-activated protein kinase 10, Mitogen-activated protein kinase 9	2/2	2	3	2	**1.10**
**306**	Mat1a P13444: S-adenosylmethionine synthase isoform type-1		4	11	10	**0.86**
**307**	Mat2a P18298: S-adenosylmethionine synthase isoform type-2		2	2	2	**0.90**
**308**	Mat2b Q5U2R0: Methionine adenosyltransferase 2 subunit beta		3	3	3	**0.73***
**309**	Mccc2 Q5XIT9: Methylcrotonoyl-CoA carboxylase beta chain, mitochondrial		2	2	2	**0.59***
**310**	Mdh1 O88989: Malate dehydrogenase, cytoplasmic		9	29	26	**0.73***
**311**	Mdh2 P04636: Malate dehydrogenase, mitochondrial		13	42	35	**0.28***
**312**	Metap2 P38062: Methionine aminopeptidase 2		3	3	3	**0.74**
**313**	Mif P30904: Macrophage migration inhibitory factor		2	7	7	**0.59**
**314**	Mpi Q68FX1: Mannose-6-phosphate isomerase		2	2	2	**0.52**
**315**	Mpst P97532: 3-mercaptopyruvate sulfurtransferase		5	12	9	**0.57***
**316**	Mri1 Q5HZE4: Methylthioribose-1-phosphate isomerase		4	4	3	**0.76***
**317**	Msra Q923M1: Mitochondrial peptide methionine sulfoxide reductase		2	2	2	**0.82**
**318**	Mt2 P04355: Metallothionein-2		2	3	3	**2.50**
**319**	Mug1 Q03626: Murinoglobulin-1		30	101	21	**2.92***
**321**	Myh10 Q9JLT0: Myosin-10		4	5	1	**0.66***
**322**	Myh11 Q63862: Myosin-11		8	11	3	**0.78**
**323**	Myh9 Q62812: Myosin-9		21	28	15	**0.73***
**324**	Myl12b P18666, Rlc-a P13832: Myosin regulatory light chain 12B, Myosin regulatory light chain RLC-A	2/2	3	4	3	**0.69**
**325**	Myl6 Q64119: Myosin light polypeptide 6		5	6	5	**0.73**
**326**	Mylk2 P20689: Myosin light chain kinase 2, skeletal/cardiac muscle		3	3	2	**0.77***

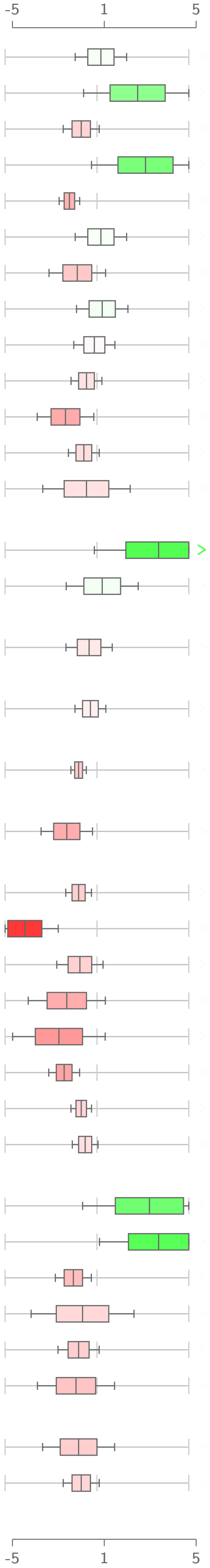

#	protein	group	peptides	spectra	quant	ratio
327	Nap1l1 Q9Z2G8: Nucleosome assembly protein 1-like 1		2	5	3	**0.75**
328	Nap1l4 Q5U2Z3: Nucleosome assembly protein 1-like 4		4	6	3	**0.64***
329	Ncl P13383: Nucleolin		15	27	20	**0.72**
330	Nedd4 Q62940: E3 ubiquitin-protein ligase NEDD4		3	4	3	**0.73***
331	Ngfg P00758: Kallikrein-1		3	4	3	**0.45***
332	Nit1 Q7TQ94: Nitrilase homolog 1		5	9	7	**0.76***
333	Nit2 Q497B0: Omega-amidase NIT2		9	13	8	**0.61***
334	Nme1 Q05982: Nucleoside diphosphate kinase A		9	36	7	**1.05**
335	Nme2 P19804: Nucleoside diphosphate kinase B		8	58	23	**0.79**
336	Nmral1 P86172: NmrA-like family domain-containing protein 1		2	2	2	**0.72***
337	Npm1 P13084: Nucleophosmin		3	3	3	**1.18**
338	Nsfl1c O35987: NSFL1 cofactor p47		7	7	6	**0.88**
339	Nucb2 Q9JI85: Nucleobindin-2		6	7	5	**0.68**
340	Nudc Q63525: Nuclear migration protein nudC		3	4	3	**0.93**
341	Nudt5 Q6AY63: ADP-sugar pyrophosphatase		4	8	6	**0.78**
342	Nutf2 P61972: Nuclear transport factor 2		2	2	2	**0.67***
343	Ola1 A0JPJ7: Obg-like ATPase 1		9	14	10	**1.14**
344	Oplah P97608: 5-oxoprolinase		6	6	5	**0.83***
345	Orm1 P02764: Alpha-1-acid glycoprotein		3	4	3	**6.85***
346	Osgep Q9WVS2: Probable tRNA N6-adenosine threonylcarbamoyltransferase		2	3	3	**0.68***
347	Ostf1 Q6P686: Osteoclast-stimulating factor 1		3	6	5	**0.61***
348	Otub1 B2RYG6: Ubiquitin thioesterase OTUB1		6	8	7	**0.93**
349	Oxct1 B2GV06: Succinyl-CoA:3-ketoacid coenzyme A transferase 1, mitochondrial		2	2	2	**0.46***
350	P01835: Ig kappa chain C region, B allele		2	5	3	**4.39***
351	P01836: Ig kappa chain C region, A allele		2	3	1	**41.46**
352	P02761: Major urinary protein		5	5	3	**2.95***
353	P08932: T-kininogen 2		7	10	2	**4.97***
354	P11517: Hemoglobin subunit beta-2		10	36	16	**1.29***
355	P20759: Ig gamma-1 chain C region		3	4	1	**8.96***
356	P32821, P32822: Trypsin V-A, Trypsin V-B	2/2	6	26	22	**0.75**
357	P4hb P04785: Protein disulfide-isomerase		30	108	89	**0.76**
358	P56571: ES1 protein homolog, mitochondrial		2	2	2	**0.40***
359	Pabpc1 Q9EPH8: Polyadenylate-binding protein 1		12	14	12	**1.37**
360	Pafah1b1 P63004: Platelet-activating factor acetylhydrolase IB subunit alpha		5	7	6	**0.68***
361	Paics P51583: Multifunctional protein ADE2 [Includes: Phosphoribosylaminoimidazole-succino . . .		2	2	2	**1.28**
362	Park7 O88767: Protein DJ-1		12	29	23	**0.72***
363	Pbld Q68G31: Phenazine biosynthesis-like domain-containing protein		5	10	7	**0.79***

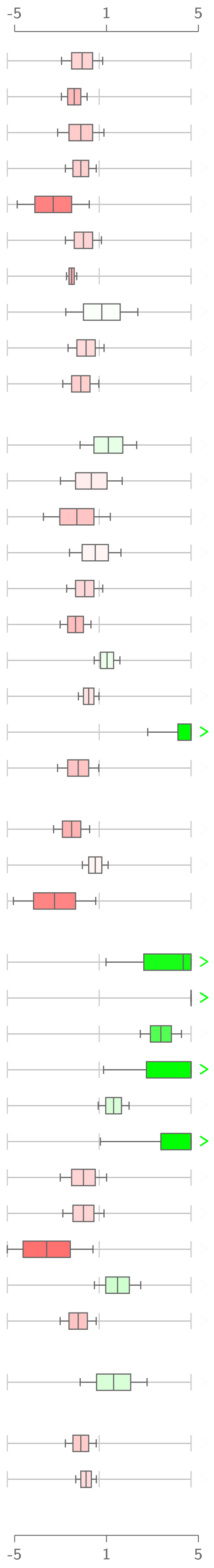

#	protein	group	peptides	spectra	quant	ratio
**364**	Pc P52873: Pyruvate carboxylase, mitochondrial		2	2	2	**0.66***
**365**	Pdap1 Q62785: 28 kDa heat- and acid-stable phosphoprotein		2	4	3	**1.22**
**366**	Pdcd6ip Q9QZA2: Programmed cell death 6-interacting protein		9	10	8	**0.78**
**367**	Pdia3 P11598: Protein disulfide-isomerase A3		18	34	29	**0.69***
**368**	Pdia4 P38659: Protein disulfide-isomerase A4		4	4	3	**0.75**
**369**	Pdia6 Q63081: Protein disulfide-isomerase A6		12	21	20	**1.23**
**370**	Pdlim1 P52944: PDZ and LIM domain protein 1		2	3	3	**0.90**
**371**	Pdxk O35331: Pyridoxal kinase		3	5	5	**0.73***
**372**	Pebp1 P31044: Phosphatidylethanolamine-binding protein 1		7	13	9	**0.80***
**373**	Pecr Q9WVK3: Peroxisomal trans-2-enoyl-CoA reductase		2	3	2	**0.15*** <
**374**	Pepd Q5I0D7: Xaa-Pro dipeptidase		6	6	5	**0.73**
**375**	Pfdn2 B0BN18: Prefoldin subunit 2		2	2	2	**0.78**
**376**	Pfkl P30835: ATP-dependent 6-phosphofructokinase, liver type		2	2	2	**0.88**
**377**	Pfn1 P62963: Profilin-1		9	23	18	**0.71***
**378**	Pgam1 P25113: Phosphoglycerate mutase 1	1/2	9	15	9	**0.84**
**379**	Pgd P85968: 6-phosphogluconate dehydrogenase, decarboxylating		4	4	3	**0.73**
**380**	Pgk1 P16617: Phosphoglycerate kinase 1		13	25	23	**0.70***
**381**	Pgls P85971: 6-phosphogluconolactonase		6	13	10	**0.82***
**382**	Pgm1 P38652: Phosphoglucomutase-1		8	11	10	**0.83***
**383**	Phgdh O08651: D-3-phosphoglycerate dehydrogenase		11	24	19	**0.76***
**384**	Pkm P11980: Pyruvate kinase PKM		14	20	17	**0.71***
**385**	Pla2g1b P04055: Phospholipase A2		6	25	19	**0.42***
**386**	Plaa P54319: Phospholipase A-2-activating protein		2	2	1	**0.84**
**387**	Pls3 Q63598: Plastin-3		2	3	3	**0.73***
**388**	Pnlip P27657: Pancreatic triacylglycerol lipase		22	203	156	**0.77***
**389**	Pnliprp1 P54316: Inactive pancreatic lipase-related protein 1		25	73	63	**0.79**
**390**	Pnliprp2 P54318: Pancreatic lipase-related protein 2		2	3	3	**0.74**
**391**	Pnp P85973: Purine nucleoside phosphorylase		9	10	8	**0.68***
**392**	Ppat P35433: Amidophosphoribosyltransferase		3	3	3	**0.75**
**393**	Ppia P10111: Peptidyl-prolyl cis-trans isomerase A		9	24	17	**0.71***
**394**	Ppib P24368: Peptidyl-prolyl cis-trans isomerase B		3	4	3	**0.96**
**395**	Ppid Q6DGG0: Peptidyl-prolyl cis-trans isomerase D		4	6	4	**0.89**
**396**	Ppp1ca P62138, Ppp1cb P62142, Ppp1cc P63088: Serine/threonine-protein phosphatase PP1-alpha catalytic subunit, Serine/threonine-protein phosphatase PP1-beta catalytic subunit, Serine/threonine-protein phosphatase PP1-gamma catalytic subunit	3/3	5	7	7	**0.93**

#	protein	group	peptides	spectra	quant	ratio
**397**	Ppp1r7 Q5HZV9: Protein phosphatase 1 regulatory subunit 7		2	3	3	**0.79**
**398**	Ppp2ca P63331: Serine/threonine-protein phosphatase 2A catalytic subunit alpha isoform	1/2	3	3	1	**1.01**
**399**	Ppp2r1b Q4QQT4: Serine/threonine-protein phosphatase 2A 65 kDa regulatory subunit A beta isoform		2	3	3	**0.58***
**400**	Ppp3ca P63329: Serine/threonine-protein phosphatase 2B catalytic subunit alpha isoform	1/2	6	6	3	**0.97**
**401**	Ppp6c Q64620: Serine/threonine-protein phosphatase 6 catalytic subunit		2	3	3	**0.61***
**402**	Prdx1 Q63716: Peroxiredoxin-1		9	30	15	**1.55***
**403**	Prdx2 P35704: Peroxiredoxin-2		4	18	14	**0.82**
**404**	Prdx3 Q9Z0V6: Thioredoxin-dependent peroxide reductase, mitochondrial		3	4	3	**0.57**
**405**	Prdx4 Q9Z0V5: Peroxiredoxin-4		9	27	12	**0.91**
**406**	Prdx5 Q9R063: Peroxiredoxin-5, mitochondrial		10	18	13	**0.59***
**407**	Prdx6 O35244: Peroxiredoxin-6		5	6	4	**0.76***
**408**	Prep O70196: Prolyl endopeptidase		2	3	3	**0.89**
**409**	Prkaca P27791: cAMP-dependent protein kinase catalytic subunit alpha		3	3	1	**0.75***
**410**	Prkacb P68182: cAMP-dependent protein kinase catalytic subunit beta		4	6	3	**1.20**
**411**	Prkar1a P09456: cAMP-dependent protein kinase type I-alpha regulatory subunit [Cleaved into: ...		2	3	3	**0.96**
**412**	Prkar2a P12368: cAMP-dependent protein kinase type II-alpha regulatory subunit		2	2	2	**0.80***
**413**	Prrc1 Q3T1I4: Protein PRRC1		4	4	3	**1.14**
**414**	Prss1 P00762: Anionic trypsin-1		3	77	57	**0.68***
**415**	Prss2 P00763: Anionic trypsin-2		6	11	8	**0.78**
**416**	Psap P10960: Sulfated glycoprotein 1		2	2	2	**0.99**
**417**	Psma1 P18420: Proteasome subunit alpha type-1		4	4	3	**0.82**
**418**	Psma2 P17220: Proteasome subunit alpha type-2		2	7	6	**0.90**
**419**	Psma5 P34064: Proteasome subunit alpha type-5		2	2	2	**0.91**
**420**	Psma6 P60901: Proteasome subunit alpha type-6		2	2	1	**0.97**
**421**	Psmb1 P18421: Proteasome subunit beta type-1		2	2	2	**1.20**
**422**	Psmd13 B0BN93: 26S proteasome non-ATPase regulatory subunit 13		2	2	2	**0.40***
**423**	Psme2 Q63798: Proteasome activator complex subunit 2		2	2	2	**0.64***
**424**	Ptbp1 Q00438: Polypyrimidine tract-binding protein 1	1/2	3	7	3	**1.71***
**425**	Pter Q63530: Phosphotriesterase-related protein		4	6	5	**1.08**
**426**	Ptges3 P83868: Prostaglandin E synthase 3		3	6	5	**0.94**
**427**	Ptma P06302: Prothymosin alpha [Cleaved into: Prothymosin alpha, N-terminally processed; ...		3	5	3	**0.54***
**428**	Ptms P04550: Parathymosin		3	11	9	**0.73**

#	protein	group	peptides	spectra	quant	ratio
**429**	Ptpn6 P81718: Tyrosine-protein phosphatase non-receptor type 6		3	3	3	**1.30**
**430**	Pura P86252: Transcriptional activator protein Pur-alpha		5	10	8	**0.60***
**431**	Purb Q68A21: Transcriptional activator protein Pur-beta		4	10	10	**0.88**
**432**	Pygb P53534: Glycogen phosphorylase, brain form	1/2	5	6	3	**0.69***
**433**	Q00715: Histone H2B type 1		2	2	2	**0.72***
**434**	Q5U2Q3: Ester hydrolase C11orf54 homolog		5	9	8	**0.60***
**435**	Qdpr P11348: Dihydropteridine reductase		4	5	4	**0.82***
**436**	Rab11a P62494, Rab11b O35509: Ras-related protein Rab-11A, Ras-related protein Rab-11B	2/2	2	2	2	**1.35***
**437**	Rab14 P61107: Ras-related protein Rab-14		2	2	1	**1.78***
**438**	Rab1A Q6NYB7: Ras-related protein Rab-1A	1/7	4	7	2	**1.35***
**439**	Rab2a P05712: Ras-related protein Rab-2A		3	4	3	**1.48***
**440**	Rab3a P63012, Rab3b Q63941, Rab3c P62824, . . . : Ras-related protein Rab-3A, Ras-related protein Rab-3B, Ras-related protein Rab-3C, GTP-binding protein Rab-3D	4/4	2	2	1	**1.80***
**441**	Rad23b Q4KMA2: UV excision repair protein RAD23 homolog B		4	4	4	**0.91**
**442**	Ran P62828: GTP-binding nuclear protein Ran	1/2	7	17	2	**0.51***
**443**	Rars P40329: Arginine--tRNA ligase, cytoplasmic		6	6	6	**0.97**
**444**	Rbbp9 O88350: Putative hydrolase RBBP9		3	4	4	**0.59***
**445**	Rbm8a Q27W01: RNA-binding protein 8A		2	2	2	**0.88**
**446**	Rbp4 P04916: Retinol-binding protein 4		2	2	2	**3.95***
**447**	Reg1 P10758: Lithostathine		5	21	11	**0.51**
**448**	Rexo2 Q5U1X1: Oligoribonuclease, mitochondrial		3	6	5	**0.66***
**449**	Rhoa P61589: Transforming protein RhoA	1/2	7	9	4	**0.75***
**450**	Rnase1 P00684: Ribonuclease pancreatic beta-type		6	37	35	**0.54**
**451**	Rnh1 P29315: Ribonuclease inhibitor		20	79	63	**0.76***
**452**	Rnpep O09175: Aminopeptidase B		10	16	11	**0.79***
**453**	Rpl10a P62907: 60S ribosomal protein L10a		2	3	3	**1.03**
**454**	Rplp0 P19945: 60S acidic ribosomal protein P0		2	5	5	**0.59***
**455**	Rplp2 P02401: 60S acidic ribosomal protein P2		4	8	7	**0.79**
**456**	Rps10 P63326: 40S ribosomal protein S10		2	4	3	**0.60**
**457**	Rps19 P17074: 40S ribosomal protein S19		4	4	3	**1.41**
**458**	Rps27a P62982, Uba52 P62986, Ubb P0CG51, . . . : Ubiquitin-40S ribosomal protein S27a, Ubiquitin-60S ribosomal protein L40, Polyubiquitin-B [Cleaved into: Ubiquitin], Polyubiquitin-C [Cleaved into: Ubiquitin; Ubiquitin-related]	4/4	4	12	10	**1.07**
**459**	Rps3 P62909: 40S ribosomal protein S3		3	3	3	**0.69**
**460**	S100a11 Q6B345: Protein S100-A11		2	5	3	**0.67***
**461**	Sae1 Q6AXQ0: SUMO-activating enzyme subunit 1		3	3	3	**0.68**

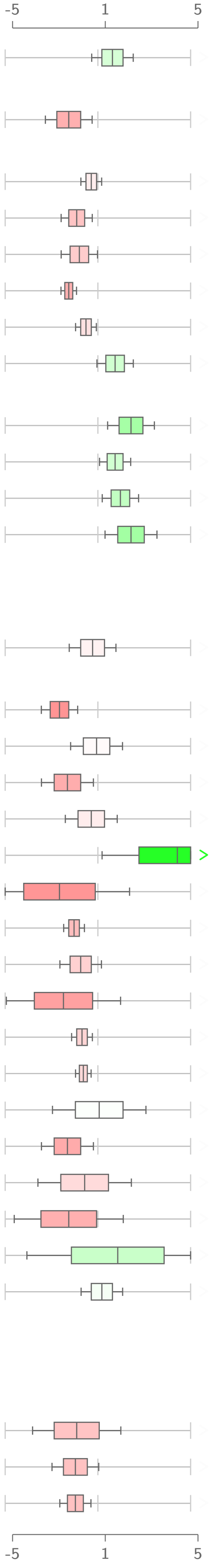

#	protein	group	peptides	spectra	quant	ratio
462	Sar1b Q5HZY2: GTP-binding protein SAR1b		2	3	3	**0.87**
463	Sardh Q64380: Sarcosine dehydrogenase, mitochondrial		3	3	3	**0.45***
464	Sars Q6P799: Serine--tRNA ligase, cytoplasmic		10	21	18	**0.80***
465	Scly Q68FT9: Selenocysteine lyase		2	2	2	**0.80***
466	Scpep1 Q920A6: Retinoid-inducible serine carboxypeptidase		3	4	3	**0.72**
467	Scrn2 Q6AYR8: Secernin-2		3	3	3	**1.06**
468	Sec31a Q9Z2Q1: Protein transport protein Sec31A		7	8	7	**1.56**
469	Selenbp1 Q8VIF7: Selenium-binding protein 1		16	34	26	**0.69***
470	Serpina1 P17475: Alpha-1-antiproteinase		13	31	28	**3.43***
471	Serpina3k P05545: Serine protease inhibitor A3K		19	42	22	**4.16***
472	Serpina3l P05544: Serine protease inhibitor A3L	1/2	18	38	12	**3.60***
473	Serpina3m Q63556: Serine protease inhibitor A3M		5	5	3	**2.57***
474	Serpina3n P09006: Serine protease inhibitor A3N		5	8	6	**1.25**
475	Serpina6 P31211: Corticosteroid-binding globulin		3	3	3	**2.45***
476	Serpinb1a Q4G075: Leukocyte elastase inhibitor A		16	61	51	**0.47***
477	Serping1 Q6P734: Plasma protease C1 inhibitor		5	6	6	**1.97***
478	Set Q63945: Protein SET		2	5	4	**0.98**
479	Skp1 Q6PEC4: S-phase kinase-associated protein 1		2	2	2	**0.72***
480	Sod1 P07632: Superoxide dismutase [Cu-Zn]		9	17	14	**0.67***
481	Sod2 P07895: Superoxide dismutase [Mn], mitochondrial		2	10	3	**0.34***
482	Spink3 P09656: Serine protease inhibitor Kazal-type 3		2	4	3	**0.50***
483	Srsf6 G3V6S8: Serine/arginine-rich splicing factor 6		3	4	4	**0.84**
484	St13 P50503: Hsc70-interacting protein		4	7	5	**0.75**
485	Stip1 O35814: Stress-induced-phosphoprotein 1		12	13	10	**0.82**
486	Stk24 B0LT89: Serine/threonine-protein kinase 24		2	2	2	**0.96**
487	Strap Q5XIG8: Serine-threonine kinase receptor-associated protein		2	2	2	**0.78***
488	Suclg1 P13086: Succinyl-CoA ligase [ADP/GDP-forming] subunit alpha, mitochondrial		4	4	4	**0.61***
489	Sumo2 P61959: Small ubiquitin-related modifier 2		2	5	4	**0.73**
490	Syncrip Q7TP47: Heterogeneous nuclear ribonucleoprotein Q		3	4	3	**0.80***
491	Tagln P31232: Transgelin		9	13	12	**0.83**
492	Tagln2 Q5XFX0: Transgelin-2		8	19	16	**0.78***
493	Taldo1 Q9EQS0: Transaldolase		13	28	18	**0.73***
494	Tars Q5XHY5: Threonine--tRNA ligase, cytoplasmic		14	29	21	**0.74***
495	Tbca Q6PEC1: Tubulin-specific chaperone A		4	4	3	**0.75***
496	Tceb2 P62870: Transcription elongation factor B polypeptide 2		4	4	3	**0.88**
497	Tes Q2LAP6: Testin		6	8	7	**1.05**
498	Tf P12346: Serotransferrin		31	115	106	**3.85***

#	protein	group	peptides	spectra	quant	ratio
**499**	Thop1 P24155: Thimet oligopeptidase	6	8	7	**0.83***
**500**	Timm8a Q9WVA1: Mitochondrial import inner membrane translocase subunit Tim8 A	2	2	2	**0.89**
**501**	Tkt P50137: Transketolase	19	39	28	**0.68***
**502**	Tmsb4x P62329: Thymosin beta-4	3	9	7	**0.71**
**503**	Tpi1 P48500: Triosephosphate isomerase	14	39	33	**0.70***
**504**	Tpm1 P04692: Tropomyosin alpha-1 chain	10	12	4	**1.01**
**506**	Tpm3 Q63610: Tropomyosin alpha-3 chain	14	17	5	**0.95**
**507**	Tpm4 P09495: Tropomyosin alpha-4 chain	12	15	6	**0.86**
**508**	Tpr F1MA98: Nucleoprotein TPR	2	3	3	**1.11**
**509**	Tpt1 P63029: Translationally-controlled tumor protein	5	9	7	**1.87***
**510**	Try3 P08426: Cationic trypsin-3	6	55	39	**0.54***
**511**	Try4 P12788: Trypsin-4	4	7	5	**0.86**
**512**	Tst P24329: Thiosulfate sulfurtransferase	5	12	8	**0.49***
**513**	Ttr P02767: Transthyretin	5	11	9	**2.52***
**516**	Tuba1c Q6AYZ1: Tubulin alpha-1C chain	15	43	1	**1.07**
**517**	Tuba4a Q5XIF6: Tubulin alpha-4A chain	17	56	6	**0.81**
**519**	Tubb3 Q4QRB4: Tubulin beta-3 chain	7	25	1	**0.62***
**520**	Tubb4b Q6P9T8: Tubulin beta-4B chain	12	57	3	**0.70***
**521**	Tubb5 P69897: Tubulin beta-5 chain	12	60	10	**0.97**
**522**	Tufm P85834: Elongation factor Tu, mitochondrial	2	2	2	**0.95**
**523**	Twf1 Q5RJR2: Twinfilin-1	2	2	2	**0.93**
**524**	Txn P11232: Thioredoxin	4	10	7	**0.78**
**525**	Txnl1 Q920J4: Thioredoxin-like protein 1	5	9	7	**0.82***
**526**	Uba1 Q5U300: Ubiquitin-like modifier-activating enzyme 1	26	51	45	**0.72***
**527**	Uba5 Q5M7A4: Ubiquitin-like modifier-activating enzyme 5	9	12	9	**0.73***
**528**	Ube2d3 P61078: Ubiquitin-conjugating enzyme E2 D3	2	2	2	**0.72***
**529**	Ube2n Q9EQX9: Ubiquitin-conjugating enzyme E2 N	5	8	6	**0.81***
**530**	Ube2v2 Q7M767: Ubiquitin-conjugating enzyme E2 variant 2	3	5	3	**0.79**
**531**	Uchl3 Q91Y78: Ubiquitin carboxyl-terminal hydrolase isozyme L3	2	2	2	**0.85**
**532**	Ufc1 Q6BBI8: Ubiquitin-fold modifier-conjugating enzyme 1	3	6	4	**0.78***
**533**	Ufm1 Q5BJP3: Ubiquitin-fold modifier 1	2	8	6	**0.59***
**534**	Uggt1 Q9JLA3: UDP-glucose:glycoprotein glucosyltransferase 1	2	2	2	**0.96**
**535**	Urod P32362: Uroporphyrinogen decarboxylase	2	4	3	**0.38**
**536**	Uso1 P41542: General vesicular transport factor p115	13	18	13	**0.83***
**537**	Vars Q04462: Valine--tRNA ligase	11	14	9	**0.70**
**538**	Vcl P85972: Vinculin	7	10	9	**0.77***

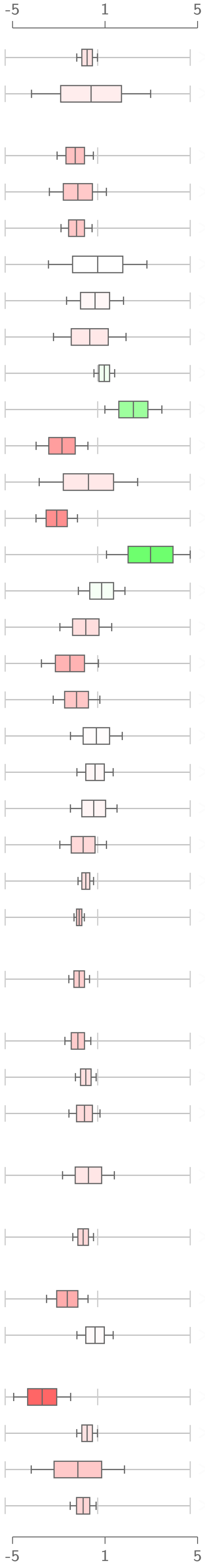

#	protein	group	peptides	spectra	quant	ratio
**539**	Vcp P46462: Transitional endoplasmic reticulum ATPase	25	45	41		**0.72***
**540**	Vim P31000: Vimentin	2	3	1		**1.14**
**541**	Wars Q6P7B0: Tryptophan--tRNA ligase, cytoplasmic	15	19	16		**0.72***
**542**	Wdr1 Q5RKI0: WD repeat-containing protein 1	7	9	7		**0.81**
**543**	Xdh P22985: Xanthine dehydrogenase/oxidase [Includes: Xanthine dehydrogenase	6	7	5		**0.78***
**544**	Xpnpep1 O54975: Xaa-Pro aminopeptidase 1	4	4	3		**0.69***
**545**	Xpo1 Q80U96: Exportin-1	2	3	3		**1.01**
**546**	Yars Q4KM49: Tyrosine--tRNA ligase, cytoplasmic	16	22	15		**0.72***
**547**	Ybx1 P62961: Nuclease-sensitive element-binding protein 1	3	9	6		**1.02**
**548**	Ybx3 Q62764: Y-box-binding protein 3	4	5	3		**0.58***
**549**	Ywhab P35213: 14-3-3 protein beta/alpha	10	19	6		**0.79**
**550**	Ywhae P62260: 14-3-3 protein epsilon	15	35	24		**0.86**
**551**	Ywhag P61983: 14-3-3 protein gamma [Cleaved into: 14-3-3 protein gamma, N-terminally proce ...	13	27	11		**0.87**
**552**	Ywhah P68511: 14-3-3 protein eta	8	19	5		**0.76**
**553**	Ywhaq P68255: 14-3-3 protein theta	13	23	10		**0.75**
**554**	Ywhaz P63102: 14-3-3 protein zeta/delta	13	37	20		**0.76***
**555**	Zg16 Q8CJD3: Zymogen granule membrane protein 16	3	8	7		**0.98**

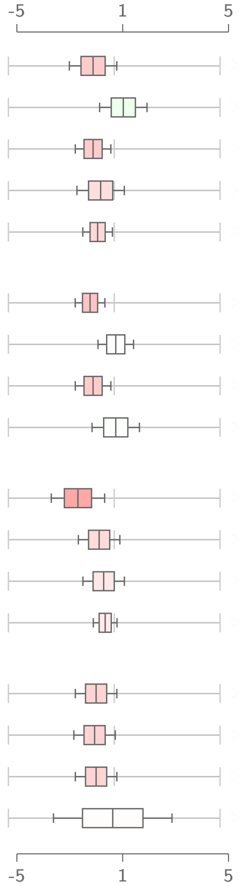

2.2 Not Quantified Proteins

Number of identified but not quantified proteins:

#	protein	group	peptides	spectra
505	Tpm2 P58775: Tropomyosin beta chain		8	9
514	Tuba1a P68370: Tubulin alpha-1A chain	1/3	16	56
66	Arf5 P84083: ADP-ribosylation factor 5		5	14
518	Tubb2a P85108, Tubb2b Q3KRE8: Tubulin beta-2A chain, Tubulin beta-2B chain	2/2	10	47
320	Mug2 Q6IE52: Murinoglobulin-2		21	74
515	Tuba1b Q6P9V9: Tubulin alpha-1B chain		16	58
