# Supplementary material for: Data for Tandem Mass Tag (TMT) proteomic analysis of the pancreas during the early phase of experimental pancreatitis
Source: Data Brief. 2018 Aug 30;20:779–83. doi: 10.1016/j.dib.2018.08.142 (PMC6129721; doi:10.1016/j.dib.2018.08.142)
Supplement: Supplementary file 3 — Supplementary material [file mmc3.pdf]

Isobar QC Report - WHOLE MEMBRANE FRACTION

December 17, 2014

Reporter Mass Precision

Histogram representing the distribution of the delta mass (in m/z) for each reporter tag between theoretical and observed mass. Reporter tag ions were extracted from MS2 fragment spectra at ± 0.05 m/z around the theoretical masses.

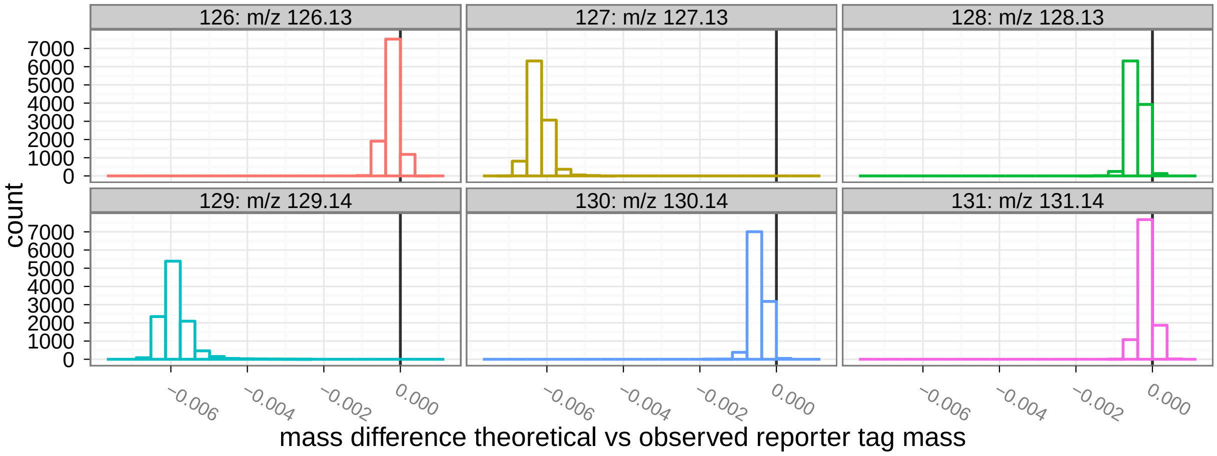

Reporter Intensities Normalization

Box-plot of the reporter intensities before and after the normalization process. Isobar applies a normalization factor to impose equal median or summed intensity in each channel. Some channels may be excluded from normalization, as defined in the `properties.R`.

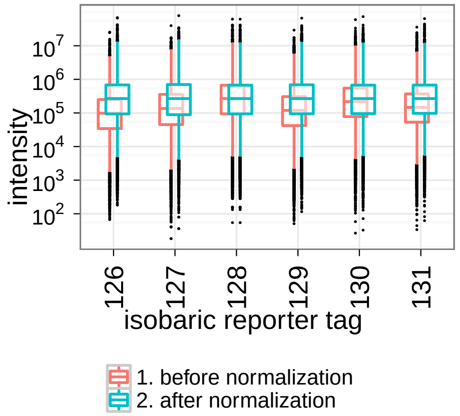

		before norm.		after norm.		
tag	NA[a]	mean	median	mean	median	factor[b]
126	0.26	270447.9	98053.1	737571.8	267412.6	0.3667
127	0.23	380349.3	135777.9	749092.3	267412.6	0.5077
128	0.19	703373.4	267412.6	703373.4	267412.6	1
129	0.26	337568.0	118983.5	758676.5	267412.6	0.4449
130	0.30	578940.1	217704.6	711128.2	267412.6	0.8141
131	0.18	393830.6	146440.5	719167.4	267412.6	0.5476

[a] Percentage of spectra with no quantitative information
[b] n.n. . . . excluded from normalization

* This report was generated using the `isobar` R package version 1.9.3.2 [built using R 2.14.1; ; 2014-02-07 09:57:05 UTC; unix]. If you use it in published work, please cite 'Breitwieser FP *et al.*: General statistical modeling of data from protein relative expression isobaric tags, *Journal of Proteome Research* 2011' and 'Breitwieser FP and Colinge J: isobarPTM: A software tool for the quantitative analysis of post-translationally modified proteins, *Journal of Proteomics* 2013'

Ratio Distribution

Distribution of the computed protein ratios (bars) and the probability density function of a Cauchy distribution (solid line) fitted as described below.

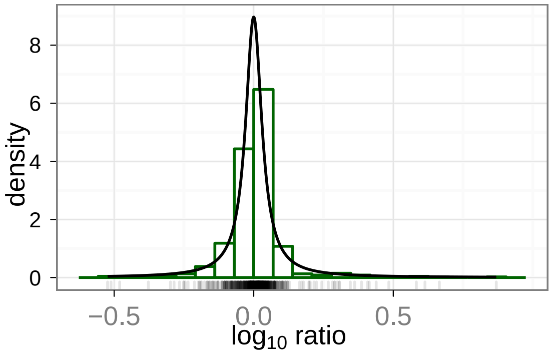


```
Distribution Object of Class: Cauchy
 location: 0
 scale: 0.03549
```

probabilities:	0.5%	1%	2.5%	5%	95%	97.5%	99%	99.5%
distribution quantiles (log_{10}):	-2.26	-1.13	-0.45	-0.22	0.22	0.45	1.13	2.26
(normal scale):	0.006	0.074	0.354	0.597	1.675	2.824	13.468	181.617

Individual Ratio Distributions

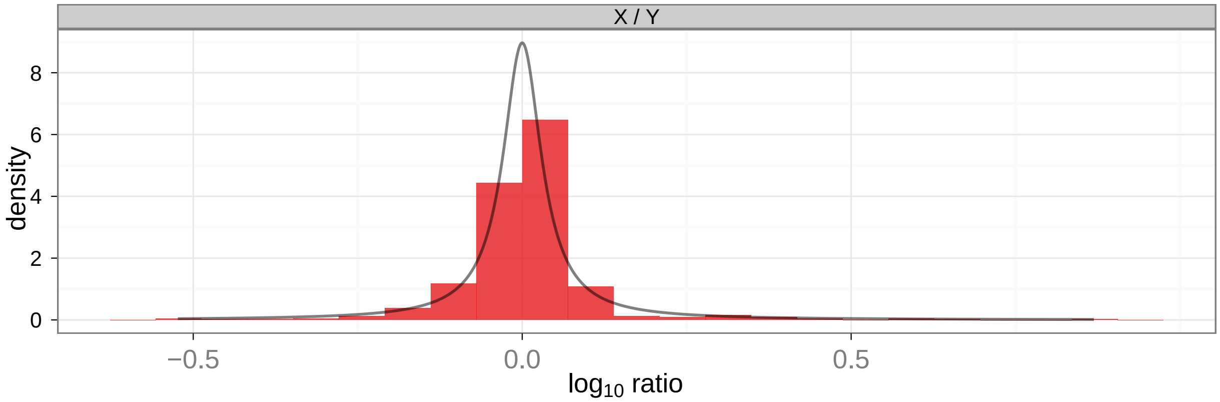

	class2	class1	res[[1]]
1	X	Y	681
2	X	Y	39
3	X	Y	38

Ratio-intensity Plot

Display of the ratio (*y-axis*) versus the log10 average signal intensity (*x-axis*) for all the reporter ratio combinations. The noise model used by Isobar is indicated as a solid red line.

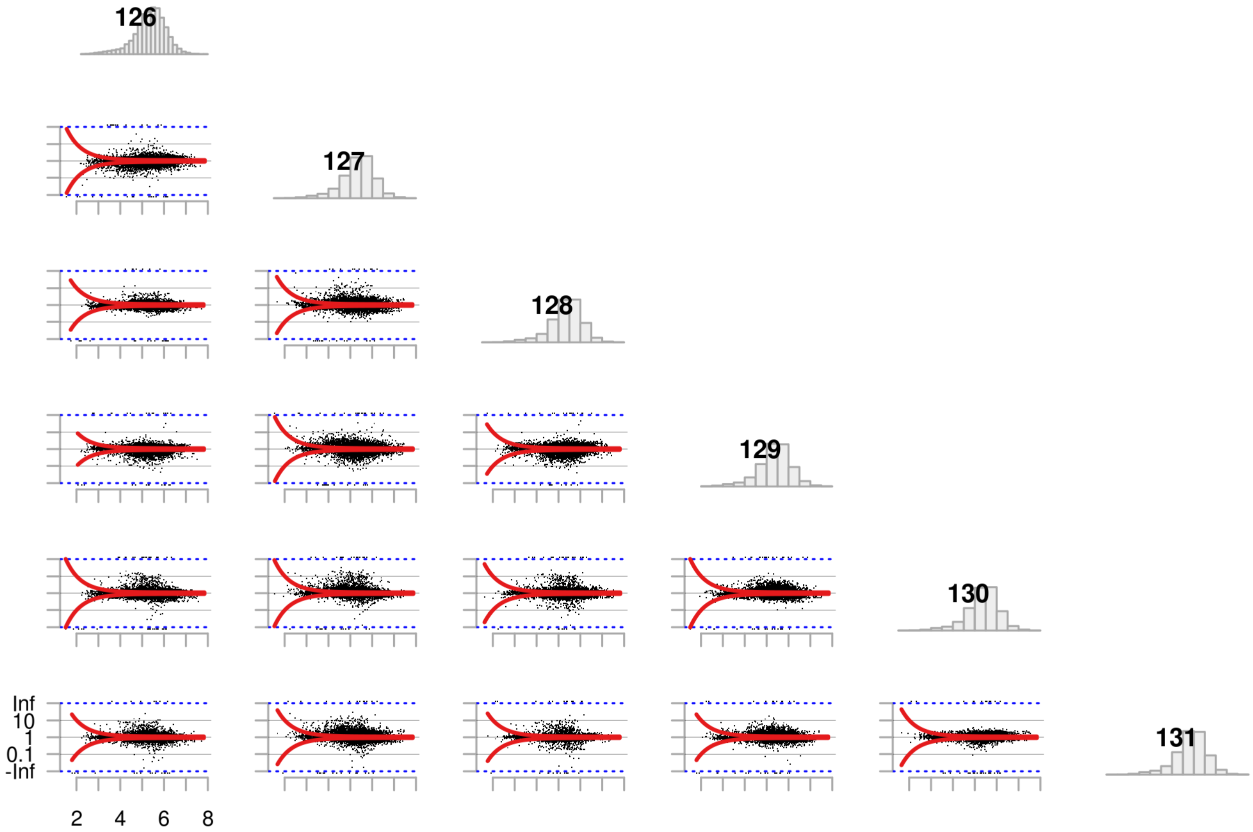
126
127
128
129
130
131
Inf
10
1
0.1
-Inf
2
4
6
8

Isobaric Tag Quantification Report Job_1418810565091 *

vig

December 17, 2014

Channel	Class
126	*Y*
127	*Y*
128	*Y*
129	*X*
130	*X*
131	*X*

Column Descriptions

protein	Protein name, accession code, and description.
group	Protein group size, only displayed if it is not '1/1'. First number: Number of group reporters; second number: group size. See Protein Group Details for details.
peptides	Number of peptides for protein.
spectra	Number of idenfitied spectra for protein.
quant	Number of spectra with quantitative information for *ch2* versus *ch1*.
ratio	Ratio of protein in channel *ch2* relative to *ch1*.
*	Significance of protein ratio in sample.

1 Significantly Regulated Proteins X / Y

Number of significant proteins:

#	protein	group	peptides	spectra	quant	ratio
1	Mcfd2 Q8K5B3: Multiple coagulation factor deficiency protein 2 homolog		2	3	3	**0.30**
2	Pdcd4 Q9JID1: Programmed cell death protein 4		2	2	2	**0.31**
3	Prdx1 Q63716: Peroxiredoxin-1		7	15	9	**0.33**
4	Jagn1 Q4KM64: Protein jagunal homolog 1		3	3	3	**0.42**
5	Tpt1 P63029: Translationally-controlled tumor protein		2	2	2	**0.50**
6	Hnrnpa2b1 A7VJC2: Heterogeneous nuclear ribonucleoproteins A2/B1		2	2	1	**0.52**
7	Arpc1b O88656: Actin-related protein 2/3 complex subunit 1B		2	2	2	**0.56**
8	Mlycd Q920F5: Malonyl-CoA decarboxylase, mitochondrial		2	2	2	**0.58**
9	Actr3 Q4V7C7: Actin-related protein 3		4	10	7	**0.61**
10	Gcg P06883: Glucagon [Cleaved into: Glicentin; Glicentin-related polypeptide		4	11	9	**0.64**
11	Ap1b1 P52303: AP-1 complex subunit beta-1		9	14	4	**0.64**
12	Actr2 Q5M7U6: Actin-related protein 2		2	2	2	**0.65**

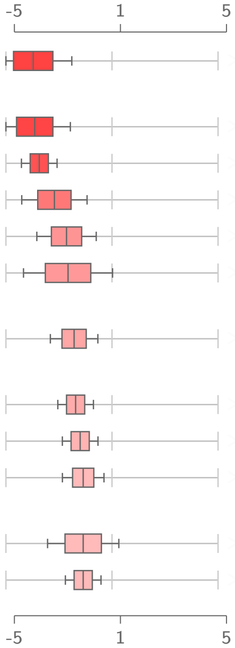

*This report was generated using the isobar R package version 1.9.3.2 [built using R 2.14.1; ; 2014-02-07 09:57:05 UTC; unix]. If you use it in published work, please cite 'Breitwieser FP *et al.*: General statistical modeling of data from protein relative expression isobaric tags, *Journal of Proteome Research* 2011' and 'Breitwieser FP and Colinge J: isobarPTM: A software tool for the quantitative analysis of post-translationally modified proteins,*Journal of Proteomics* 2013'

#	protein	group	peptides	spectra	quant	ratio
13	Pabpc1 Q9EPH8: Polyadenylate-binding protein 1		12	24	21	**0.67**
14	Eef2 P05197: Elongation factor 2		39	149	134	**0.68**
15	Hnrnpk P61980: Heterogeneous nuclear ribonucleoprotein K		8	9	9	**0.69**
16	Cs Q8VHF5: Citrate synthase, mitochondrial		5	9	7	**0.69**
17	Arfgap3 Q4KLN7: ADP-ribosylation factor GTPase-activating protein 3		6	8	6	**0.69**
18	Cdv3 Q5XIM5: Protein CDV3 homolog		2	3	3	**0.70**
19	Arl6ip5 Q9ES40: PRA1 family protein 3		3	6	6	**0.71**
20	Marcks P30009: Myristoylated alanine-rich C-kinase substrate		2	2	2	**0.71**
21	Eif4a3 Q3B8Q2: Eukaryotic initiation factor 4A-III		3	5	1	**0.72**
22	Tkt P50137: Transketolase		3	3	3	**0.72**
23	Vps29 B2RZ78: Vacuolar protein sorting-associated protein 29		5	6	4	**0.73**
24	Aldh1l1 P28037: Cytosolic 10-formyltetrahydrofolate dehydrogenase		11	14	10	**0.74**
25	Cltb P08082: Clathrin light chain B		4	4	4	**0.75**
26	Mcts1 Q4G009: Malignant T-cell-amplified sequence 1		2	3	3	**0.77**
27	Actr1a P85515: Alpha-centractin		3	3	3	**0.77**
28	Arcn1 Q66H80: Coatomer subunit delta		18	33	31	**0.79**
29	Copg1 Q4AEF8: Coatomer subunit gamma-1		19	48	33	**0.80**
30	Eef1d Q68FR9: Elongation factor 1-delta		5	17	13	**0.80**
31	Rps28 P62859: 40S ribosomal protein S28		2	7	7	**0.81**
32	Rpl36 P39032: 60S ribosomal protein L36		3	6	5	**0.83**
33	Basp1 Q05175: Brain acid soluble protein 1		2	2	2	**0.83**
34	Calm1 P62161: Calmodulin	1/2	5	7	3	**0.83**
35	Psma6 P60901: Proteasome subunit alpha type-6		2	2	2	**0.83**
36	Edf1 P69736: Endothelial differentiation-related factor 1		2	3	3	**0.83**
37	Pfkl P30835: ATP-dependent 6-phosphofructokinase, liver type		2	2	2	**0.85**
38	Copb1 P23514: Coatomer subunit beta		30	67	61	**0.86**
39	Hspa8 P63018: Heat shock cognate 71 kDa protein	1/4	19	40	26	**0.88**
40	Uqcrc2 P32551: Cytochrome b-c1 complex subunit 2, mitochondrial		9	23	20	**1.12**
41	Etf1 Q5U2Q7: Eukaryotic peptide chain release factor subunit 1		9	15	12	**1.12**
42	Hsd17b4 P97852: Peroxisomal multifunctional enzyme type 2		4	4	3	**1.14**
43	Rab27b Q99P74: Ras-related protein Rab-27B		3	5	3	**1.14**
44	Sfxn1 Q63965: Sideroflexin-1		7	13	10	**1.15**
45	Gde1 Q9JL55: Glycerophosphodiester phosphodiesterase 1		2	2	2	**1.17**
46	Rhot2 Q7TSA0: Mitochondrial Rho GTPase 2		2	2	2	**1.19**
47	Yars2 Q5I0L3: Tyrosine--tRNA ligase, mitochondrial		2	2	2	**1.19**
48	Slc25a11 P97700: Mitochondrial 2-oxoglutarate/malate carrier protein		7	14	12	**1.22**
49	Atp1b3 Q63377: Sodium/potassium-transporting ATPase subunit beta-3		2	4	3	**1.22**
50	Slc38a5 A2VCW5: Sodium-coupled neutral amino acid transporter 5		4	7	6	**1.24**

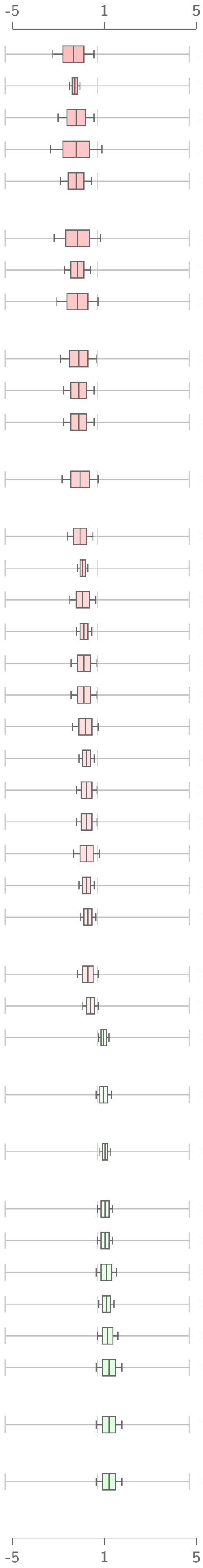

#	protein	group	peptides	spectra	quant	ratio
51	Rap1a P62836: Ras-related protein Rap-1A	6	9	1	**1.24**
52	Gnai3 P08753: Guanine nucleotide-binding protein G(k) subunit alpha	2	2	1	**1.25**
53	Lamp2 P17046: Lysosome-associated membrane glycoprotein 2	2	3	3	**1.27**
54	Rab8a P35280: Ras-related protein Rab-8A	3	7	1	**1.29**
55	Ralb P36860: Ras-related protein Ral-B	2	4	1	**1.30**
56	Sfxn3 Q9JHY2: Sideroflexin-3	2	6	3	**1.31**
57	Arf4 P61751: ADP-ribosylation factor 4	8	17	4	**1.32**
58	Epcam O55159: Epithelial cell adhesion molecule	2	2	2	**1.46**
59	Atp1b1 P07340: Sodium/potassium-transporting ATPase subunit beta-1	2	3	3	**1.51**
60	Cox5a P11240: Cytochrome c oxidase subunit 5A, mitochondrial	4	8	8	**1.58**
61	Clu P05371: Clusterin	4	7	6	**1.68**
62	Fn1 P04937: Fibronectin	2	3	3	**1.75**
63	Ifrd1 P20695: Interferon-related developmental regulator 1	3	4	3	**1.91**
64	Igg-2a P20760: Ig gamma-2A chain C region	2	2	2	**1.95**
65	C4 P08649: Complement C4 [Cleaved into: Complement C4 beta chain; Complement C4 alpha c . . .	4	4	3	**1.99**
66	Apoa2 P04638: Apolipoprotein A-II	3	10	10	**2.02**
67	Fga P06399: Fibrinogen alpha chain [Cleaved into: Fibrinopeptide A; Fibrinogen alpha chain]	3	6	5	**2.03**
68	Fgg P02680: Fibrinogen gamma chain	2	2	2	**2.22**
69	Apoh P26644: Beta-2-glycoprotein 1	4	9	8	**2.30**
70	Apoc1 P19939: Apolipoprotein C-I	2	3	3	**2.43**
71	Fgb P14480: Fibrinogen beta chain	4	5	3	**2.59**
72	Apoe P02650: Apolipoprotein E	4	6	6	**2.75**
73	Hpx P20059: Hemopexin	2	2	2	**3.05**
74	Apoa1 P04639: Apolipoprotein A-I	9	20	15	**3.82**
75	P01835: Ig kappa chain C region, B allele	3	5	3	**4.11**
76	Hrg Q99PS8: Histidine-rich glycoprotein	3	4	3	**4.62**
77	C3 P01026: Complement C3 [Cleaved into: Complement C3 beta chain; C3-beta-c	2	4	3	**7.40**

2 Protein Relative Quantitation X / Y

Number of quantified proteins:

2.1 Quantified Proteins

#	protein	group	peptides	spectra	quant	ratio
**1**	A1m Q63041: Alpha-1-macroglobulin	23	54	50	**2.56**
**2**	Abcb7 Q704E8: ATP-binding cassette sub-family B member 7, mitochondrial	3	3	3	**1.03**

#	protein	group	peptides	spectra	quant	ratio
3	Abcf1 Q6MG08: ATP-binding cassette sub-family F member 1		10	12	11	**0.98**
4	Abhd6 Q5XI64: Monoacylglycerol lipase ABHD6		2	2	2	**1.18**
5	Acaa2 P13437: 3-ketoacyl-CoA thiolase, mitochondrial		2	2	2	**0.84**
6	Acadl P15650: Long-chain specific acyl-CoA dehydrogenase, mitochondrial		2	2	2	**0.57**
7	Acadsb P70584: Short/branched chain specific acyl-CoA dehydrogenase, mitochondrial		3	4	3	**1.12**
8	Acadvl P45953: Very long-chain specific acyl-CoA dehydrogenase, mitochondrial		17	29	24	**1.08**
9	Acat1 P17764: Acetyl-CoA acetyltransferase, mitochondrial		15	46	41	**1.02**
10	Acbd3 Q7TNY6: Golgi resident protein GCP60		4	5	4	**0.89**
11	Aco2 Q9ER34: Aconitate hydratase, mitochondrial		6	8	7	**0.80**
12	Acsf2 Q499N5: Acyl-CoA synthetase family member 2, mitochondrial		2	2	2	**0.92**
13	Acsl1 P18163: Long-chain-fatty-acid--CoA ligase 1	1/2	9	11	7	**1.04**
14	Acta1 P68136, Acta2 P62738, Actc1 P68035, . . . : Actin, alpha skeletal muscle, Actin, aortic smooth muscle, Actin, alpha cardiac muscle 1, Actin, gamma-enteric smooth muscle	4/4	6	16	2	**1.00**
15	Actb P60711, Actg1 P63259: Actin, cytoplasmic 1, Actin, cytoplasmic 2	2/2	12	33	16	**0.88**
16	Actn1 Q9Z1P2: Alpha-actinin-1		5	7	1	**1.02**
17	Actn4 Q9QXQ0: Alpha-actinin-4		5	7	1	**1.00**
18	Actr1a P85515: Alpha-centractin		3	3	3	**0.77***
19	Actr2 Q5M7U6: Actin-related protein 2		2	2	2	**0.65***
20	Actr3 Q4V7C7: Actin-related protein 3		4	10	7	**0.61***
21	Ahcy P10760: Adenosylhomocysteinase		2	2	2	**0.97**
22	Aifm1 Q9JM53: Apoptosis-inducing factor 1, mitochondrial		7	8	7	**1.02**
23	Aimp2 Q32PX2: Aminoacyl tRNA synthase complex-interacting multifunctional protein 2		8	15	11	**0.94**
24	Ak2 P29410: Adenylate kinase 2, mitochondrial		2	2	2	**1.10**
25	Ak3 P29411: GTP:AMP phosphotransferase AK3, mitochondrial		2	2	2	**1.06**
26	Alb P02770: Serum albumin		18	32	29	**1.85**
27	Aldh1l1 P28037: Cytosolic 10-formyltetrahydrofolate dehydrogenase		11	14	10	**0.74***
28	Aldh2 P11884: Aldehyde dehydrogenase, mitochondrial		9	15	12	**1.03**
29	Aldh3a2 P30839: Fatty aldehyde dehydrogenase		11	17	14	**1.04**
30	Aldh6a1 Q02253: Methylmalonate-semialdehyde dehydrogenase [acylating], mitochondrial		18	38	32	**1.05**
31	Aldh9a1 Q9JLJ3: 4-trimethylaminobutyraldehyde dehydrogenase		2	2	2	**1.02**
32	Amy2 P00689: Pancreatic alpha-amylase		19	116	102	**0.98**

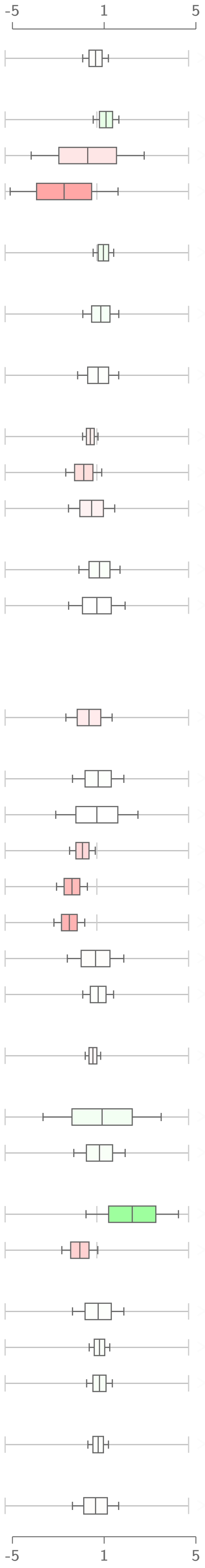

#	protein	group	peptides	spectra	quant	ratio
33	Anxa1 P07150: Annexin A1		6	8	5	**1.30**
34	Anxa2 Q07936: Annexin A2		11	13	11	**0.80**
35	Anxa4 P55260: Annexin A4		2	2	2	**0.88**
36	Anxa6 P48037: Annexin A6		22	38	36	**1.13**
37	Ap1b1 P52303: AP-1 complex subunit beta-1		9	14	4	**0.64***
38	Ap2a2 P18484: AP-2 complex subunit alpha-2		5	5	4	**1.06**
39	Ap2b1 P62944: AP-2 complex subunit beta		8	12	2	**0.81**
40	Apmap Q7TP48: Adipocyte plasma membrane-associated protein		2	4	3	**1.00**
41	Apoa1 P04639: Apolipoprotein A-I		9	20	15	**3.82***
42	Apoa2 P04638: Apolipoprotein A-II		3	10	10	**2.02***
43	Apoa4 P02651: Apolipoprotein A-IV		6	6	4	**1.95**
44	Apoc1 P19939: Apolipoprotein C-I		2	3	3	**2.43***
45	Apoe P02650: Apolipoprotein E		4	6	6	**2.75***
46	Apoh P26644: Beta-2-glycoprotein 1		4	9	8	**2.30***
47	Arcn1 Q66H80: Coatomer subunit delta		18	33	31	**0.79***
48	Arf1 P84079, Arf3 P61206: ADP-ribosylation factor 1, ADP-ribosylation factor 3	2/3	10	27	3	**1.21**
49	Arf4 P61751: ADP-ribosylation factor 4		8	17	4	**1.32***
50	Arf5 P84083: ADP-ribosylation factor 5		8	19	3	**0.98**
51	Arf6 P62332: ADP-ribosylation factor 6		6	11	7	**1.07**
52	Arfgap1 Q62848: ADP-ribosylation factor GTPase-activating protein 1		2	2	2	**1.03**
53	Arfgap2 Q3MID3: ADP-ribosylation factor GTPase-activating protein 2		2	2	2	**0.82**
54	Arfgap3 Q4KLN7: ADP-ribosylation factor GTPase-activating protein 3		6	8	6	**0.69***
55	Arfip2 Q6AY65: Arfaptin-2		3	3	3	**0.93**
56	Arl1 P61212: ADP-ribosylation factor-like protein 1		3	5	3	**1.10**
57	Arl6ip5 Q9ES40: PRA1 family protein 3		3	6	6	**0.71***
58	Arl8b Q66HA6: ADP-ribosylation factor-like protein 8B		5	13	11	**1.15**
59	Arpc1b O88656: Actin-related protein 2/3 complex subunit 1B		2	2	2	**0.56***
60	Arpc2 P85970: Actin-related protein 2/3 complex subunit 2		2	3	3	**0.71**
61	Atad3 Q3KRE0: ATPase family AAA domain-containing protein 3		5	5	3	**1.06**
62	Atl3 Q0ZHH6: Atlastin-3		3	4	3	**1.03**
63	Atp1a1 P06685: Sodium/potassium-transporting ATPase subunit alpha-1	1/5	15	31	18	**1.24**
64	Atp1b1 P07340: Sodium/potassium-transporting ATPase subunit beta-1		2	3	3	**1.51***
65	Atp1b3 Q63377: Sodium/potassium-transporting ATPase subunit beta-3		2	4	3	**1.22***

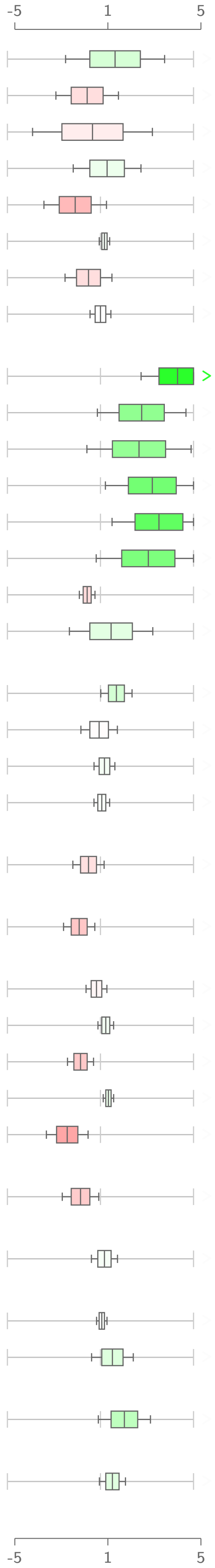

#	protein	group	peptides	spectra	quant	ratio
**66**	Atp2a2 P11507: Sarcoplasmic/endoplasmic reticulum calcium ATPase 2	1/3	21	34	19	**1.03**
**67**	Atp5a1 P15999: ATP synthase subunit alpha, mitochondrial		23	71	64	**1.09**
**68**	Atp5b P10719: ATP synthase subunit beta, mitochondrial		23	114	98	**1.11**
**69**	Atp5c1 P35435: ATP synthase subunit gamma, mitochondrial		9	18	15	**1.15**
**70**	Atp5d P35434: ATP synthase subunit delta, mitochondrial		2	5	3	**1.11**
**71**	Atp5f1 P19511: ATP synthase F(0) complex subunit B1, mitochondrial		9	18	16	**1.18**
**72**	Atp5h P31399: ATP synthase subunit d, mitochondrial		6	19	18	**1.12**
**73**	Atp5i P29419: ATP synthase subunit e, mitochondrial		2	4	3	**1.07**
**74**	Atp5j P21571: ATP synthase-coupling factor 6, mitochondrial		3	9	7	**1.22**
**75**	Atp5j2 D3ZAF6: ATP synthase subunit f, mitochondrial		2	8	7	**1.09**
**76**	Atp5o Q06647: ATP synthase subunit O, mitochondrial		10	19	18	**1.10**
**77**	Basp1 Q05175: Brain acid soluble protein 1		2	2	2	**0.83***
**78**	Bcat2 O35854: Branched-chain-amino-acid aminotransferase, mitochondrial		8	16	13	**0.98**
**79**	Bckdha P11960: 2-oxoisovalerate dehydrogenase subunit alpha, mitochondrial		10	17	14	**0.94**
**80**	Bckdhb P35738: 2-oxoisovalerate dehydrogenase subunit beta, mitochondrial		8	14	13	**1.00**
**81**	Bckdk Q00972: [3-methyl-2-oxobutanoate dehydrogenase [lipoamide]] kinase, mitochondrial		4	4	3	**1.34**
**82**	Bzw1 Q6P7P5: Basic leucine zipper and W2 domain-containing protein 1		5	6	3	**0.96**
**83**	Bzw2 Q9WTT7: Basic leucine zipper and W2 domain-containing protein 2		2	3	1	**1.10**
**84**	C1qbp O35796: Complement component 1 Q subcomponent-binding protein, mitochondrial		3	5	3	**0.84**
**85**	C3 P01026: Complement C3 [Cleaved into: Complement C3 beta chain; C3-beta-c		2	4	3	**7.40***
**86**	C4 P08649: Complement C4 [Cleaved into: Complement C4 beta chain; Complement C4 alpha c ...		4	4	3	**1.99***
**87**	Calm1 P62161: Calmodulin	1/2	5	7	3	**0.83***
**88**	Calr P18418: Calreticulin		17	55	48	**1.10**
**89**	Camk2b P08413: Calcium/calmodulin-dependent protein kinase type II subunit beta		2	2	1	**1.06**
**90**	Cand1 P97536: Cullin-associated NEDD8-dissociated protein 1		6	8	6	**0.97**
**91**	Canx P35565: Calnexin		24	59	49	**1.00**
**92**	Capza2 Q3T1K5: F-actin-capping protein subunit alpha-2		3	5	4	**0.79**
**93**	Cast P27321: Calpastatin		2	4	3	**0.90**

#	protein	group	peptides	spectra	quant	ratio
**94**	Cav1 P41350: Caveolin-1	2	2	2	**0.96**
**95**	Ccdc47 Q5U2X6: Coiled-coil domain-containing protein 47	10	16	15	**1.07**
**96**	Cckar P30551: Cholecystokinin receptor type A	2	2	2	**1.10**
**97**	Cct2 Q5XIM9: T-complex protein 1 subunit beta	6	11	10	**0.93**
**98**	Cct3 Q6P502: T-complex protein 1 subunit gamma	6	8	6	**1.04**
**99**	Cct4 Q7TPB1: T-complex protein 1 subunit delta	4	6	5	**0.97**
**100**	Cct5 Q68FQ0: T-complex protein 1 subunit epsilon	9	13	11	**0.99**
**101**	Cdc42 Q8CFN2: Cell division control protein 42 homolog	7	13	9	**1.06**
**102**	Cdipt P70500: CDP-diacylglycerol--inositol 3-phosphatidyltransferase	3	6	4	**0.88**
**103**	Cdk5rap3 Q9JLH7: CDK5 regulatory subunit-associated protein 3	12	18	15	**1.02**
**104**	Cdv3 Q5XIM5: Protein CDV3 homolog	2	3	3	**0.70***
**105**	Cel P07882: Bile salt-activated lipase	26	181	154	**1.19**
**106**	Cela1 P00773: Chymotrypsin-like elastase family member 1	2	11	9	**1.07**
**107**	Cela2a P00774: Chymotrypsin-like elastase family member 2A	8	88	76	**1.00**
**108**	Cfl1 P45592: Cofilin-1	4	9	7	**0.81**
**109**	Cisd1 B0K020: CDGSH iron-sulfur domain-containing protein 1	4	5	4	**1.00**
**110**	Clps P17084: Colipase	5	23	17	**1.04**
**111**	Clpx Q5U2U0: ATP-dependent Clp protease ATP-binding subunit clpX-like, mitochondrial	2	2	2	**0.96**
**112**	Clta P08081: Clathrin light chain A	4	4	3	**0.84**
**113**	Cltb P08082: Clathrin light chain B	4	4	4	**0.75***
**114**	Cltc P11442: Clathrin heavy chain 1	43	64	59	**0.86**
**115**	Clu P05371: Clusterin	4	7	6	**1.68***
**116**	Cml6 Q9JIY6: Probable N-acetyltransferase CML6	4	11	9	**0.97**
**117**	Cnp P13233: 2',3'-cyclic-nucleotide 3'-phosphodiesterase	6	8	5	**1.10**
**118**	Copb1 P23514: Coatomer subunit beta	30	67	61	**0.86***
**119**	Copb2 O35142: Coatomer subunit beta'	25	39	34	**0.91**
**120**	Copg1 Q4AEF8: Coatomer subunit gamma-1	19	48	33	**0.80***
**121**	Copg2 D4ABY2: Coatomer subunit gamma-2	5	14	3	**0.98**
**122**	Coq9 Q68FT1: Ubiquinone biosynthesis protein COQ9, mitochondrial	5	9	6	**0.82**
**123**	Cox4i1 P10888: Cytochrome c oxidase subunit 4 isoform 1, mitochondrial	7	15	13	**1.04**
**124**	Cox5a P11240: Cytochrome c oxidase subunit 5A, mitochondrial	4	8	8	**1.58***
**125**	Cox5b P12075: Cytochrome c oxidase subunit 5B, mitochondrial	6	15	13	**1.10**
**126**	Cox6c2 P11951: Cytochrome c oxidase subunit 6C-2	3	7	6	**1.10**

#	protein	group	peptides	spectra	quant	ratio
127	Cox7a2 P35171: Cytochrome c oxidase subunit 7A2, mitochondrial	2	5	3	**1.09**
128	Cox7c P80432: Cytochrome c oxidase subunit 7C, mitochondrial	2	3	3	**1.21**
129	Cpa1 P00731: Carboxypeptidase A1	19	58	44	**1.08**
130	Cpa2 P19222: Carboxypeptidase A2	10	38	28	**0.97**
131	Cpb1 P19223: Carboxypeptidase B	15	57	51	**1.02**
132	Cpd Q9JHW1: Carboxypeptidase D	2	2	1	**1.15**
133	Cpt1a P32198: Carnitine O-palmitoyltransferase 1, liver isoform	3	6	5	**0.96**
134	Cpt2 P18886: Carnitine O-palmitoyltransferase 2, mitochondrial	3	5	3	**1.05**
135	Creld2 Q4G063: Cysteine-rich with EGF-like domain protein 2	3	3	3	**0.88**
136	Cs Q8VHF5: Citrate synthase, mitochondrial	5	9	7	**0.69***
137	Cth P18757: Cystathionine gamma-lyase	10	19	16	**0.80**
138	Ctnnb1 Q9WU82: Catenin beta-1	4	7	4	**1.18**
139	Ctrb1 P07338: Chymotrypsinogen B	7	54	48	**1.03**
140	Ctrc P55091: Chymotrypsin-C	6	51	39	**1.27**
141	Ctsd P24268: Cathepsin D	6	10	7	**0.89**
142	Cuzd1 Q9QZT0: CUB and zona pellucida-like domain-containing protein 1	7	11	9	**1.18**
143	Cyb5a P00173: Cytochrome b5	3	4	3	**0.99**
144	Cyb5b P04166: Cytochrome b5 type B	2	5	3	**0.98**
145	Cyb5r3 P20070: NADH-cytochrome b5 reductase 3	4	5	3	**1.07**
146	Cycs P62898: Cytochrome c, somatic	3	6	5	**0.95**
147	Cyp51a1 Q64654: Lanosterol 14-alpha demethylase	2	2	2	**1.08**
148	Cyp7b1 Q63688: 25-hydroxycholesterol 7-alpha-hydroxylase	5	6	5	**1.04**
149	Dad1 P61805: Dolichyl-diphosphooligosaccharide--protein glycosyltransferase subunit DAD1	2	3	3	**1.01**
150	Dars P15178: Aspartate--tRNA ligase, cytoplasmic	18	40	34	**0.94**
151	Dctn1 P28023: Dynactin subunit 1	2	2	2	**0.95**
152	Dctn2 Q6AYH5: Dynactin subunit 2	9	12	11	**0.87**
153	Ddb1 Q9ESW0: DNA damage-binding protein 1	3	3	3	**0.99**
154	Ddost Q641Y0: Dolichyl-diphosphooligosaccharide--protein glycosyltransferase 48 kDa subunit	11	42	37	**1.03**
155	Ddx1 Q641Y8: ATP-dependent RNA helicase DDX1	20	34	31	**0.99**
156	Ddx39b Q63413: Spliceosome RNA helicase Ddx39b	2	3	3	**0.94**
157	Decr1 Q64591: 2,4-dienoyl-CoA reductase, mitochondrial	10	16	15	**0.93**
158	Dhrs4 Q8VID1: Dehydrogenase/reductase SDR family member 4	2	2	2	**1.00**
159	Dlat P08461: Dihydrolipoyllysine-residue acetyltransferase component of pyruvate dehydrog ...	8	13	10	**0.95**

#	protein	group	peptides	spectra	quant	ratio
160	Dld Q6P6R2: Dihydrolipoyl dehydrogenase, mitochondrial		10	27	24	**0.93**
161	Dlst Q01205: Dihydrolipoyllysine-residue succinyltransferase component of 2-oxoglutarate ...		3	6	5	**1.00**
162	Dnaja2 O35824: DnaJ homolog subfamily A member 2		2	2	2	**1.03**
163	Dnajb11 Q6TUG0: DnaJ homolog subfamily B member 11		8	15	12	**1.09**
164	Dnajc3 Q9R0T3: DnaJ homolog subfamily C member 3		12	15	13	**1.03**
165	Dnm2 P39052: Dynamin-2		3	3	3	**1.02**
166	Dpep1 P31430: Dipeptidase 1		7	9	7	**0.99**
167	Dstn Q7M0E3: Destrin		4	6	4	**1.03**
168	Dync1h1 P38650: Cytoplasmic dynein 1 heavy chain 1		48	60	52	**1.03**
169	Dync1i2 Q62871: Cytoplasmic dynein 1 intermediate chain 2		4	5	3	**0.86**
170	Dync1li1 Q9QXU8: Cytoplasmic dynein 1 light intermediate chain 1		2	3	3	**1.05**
171	Ech1 Q62651: Delta(3,5)-Delta(2,4)-dienoyl-CoA isomerase, mitochondrial		3	4	3	**0.83**
172	Echdc1 Q6AYG5: Ethylmalonyl-CoA decarboxylase		7	10	7	**0.87**
173	Echs1 P14604: Enoyl-CoA hydratase, mitochondrial		7	14	9	**0.90**
174	Eci1 P23965: Enoyl-CoA delta isomerase 1, mitochondrial		7	16	13	**0.83**
175	Edf1 P69736: Endothelial differentiation-related factor 1		2	3	3	**0.83***
176	Eef1a1 P62630: Elongation factor 1-alpha 1	1/2	20	71	34	**0.98**
177	Eef1d Q68FR9: Elongation factor 1-delta		5	17	13	**0.80***
178	Eef1g Q68FR6: Elongation factor 1-gamma		11	19	16	**0.77**
179	Eef2 P05197: Elongation factor 2		39	149	134	**0.68***
180	Ehd1 Q641Z6: EH domain-containing protein 1		2	2	1	**1.19**
181	Ehd2 Q4V8H8: EH domain-containing protein 2		3	3	2	**1.04**
182	Ehhadh P07896: Peroxisomal bifunctional enzyme		3	4	3	**1.04**
183	Eif1a Q6VV72: Eukaryotic translation initiation factor 1A		3	5	3	**1.04**
184	Eif2s1 P68101: Eukaryotic translation initiation factor 2 subunit 1		12	18	15	**1.05**
185	Eif2s3 P81795: Eukaryotic translation initiation factor 2 subunit 3		8	11	7	**1.01**
186	Eif3a Q1JU68: Eukaryotic translation initiation factor 3 subunit A		27	47	42	**1.00**
187	Eif3b Q4G061: Eukaryotic translation initiation factor 3 subunit B		17	35	27	**0.97**
188	Eif3c B5DFC8: Eukaryotic translation initiation factor 3 subunit C		18	27	23	**0.99**
189	Eif3d Q6AYK8: Eukaryotic translation initiation factor 3 subunit D		10	20	18	**0.99**
190	Eif3e Q641X8: Eukaryotic translation initiation factor 3 subunit E		11	20	17	**1.02**

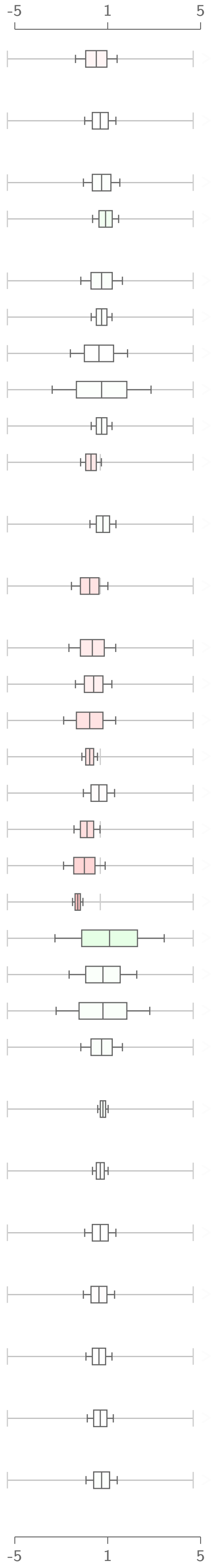

#	protein	group	peptides	spectra	quant	ratio
191	Eif3g Q5RK09: Eukaryotic translation initiation factor 3 subunit G	5	5	3	**0.97**
192	Eif3h Q6P9U8: Eukaryotic translation initiation factor 3 subunit H	9	17	16	**0.93**
193	Eif3i B0BNA7: Eukaryotic translation initiation factor 3 subunit I	9	11	9	**0.97**
194	Eif3j A0JPM9: Eukaryotic translation initiation factor 3 subunit J	9	23	17	**0.92**
195	Eif4a2 Q5RKI1: Eukaryotic initiation factor 4A-II	11	24	17	**0.91**
196	Eif4a3 Q3B8Q2: Eukaryotic initiation factor 4A-III	3	5	1	**0.72***
197	Eif5 Q07205: Eukaryotic translation initiation factor 5	10	18	14	**0.97**
198	Eif5a Q3T1J1: Eukaryotic translation initiation factor 5A-1	6	21	17	**0.98**
199	Eif5b B2GUV7: Eukaryotic translation initiation factor 5B	10	16	14	**0.84**
200	Emc2 B0BNG0: ER membrane protein complex subunit 2	4	4	3	**1.01**
201	Emc3 Q5U2V8: ER membrane protein complex subunit 3	2	2	2	**1.02**
202	Emc8 Q5FVL2: ER membrane protein complex subunit 8	3	3	3	**0.99**
203	Enpp3 P97675: Ectonucleotide pyrophosphatase/phosphodiesterase family member 3	4	7	5	**1.12**
204	Entpd2 O35795: Ectonucleoside triphosphate diphosphohydrolase 2	2	2	2	**0.94**
205	Epcam O55159: Epithelial cell adhesion molecule	2	2	2	**1.46***
206	Ephx1 P07687: Epoxide hydrolase 1	9	19	16	**1.11**
207	Erlin2 B5DEH2: Erlin-2	2	2	2	**0.77**
208	Ermp1 Q6UPR8: Endoplasmic reticulum metallopeptidase 1	3	3	3	**1.04**
209	Ero1l Q8R4A1: ERO1-like protein alpha	3	4	3	**1.14**
210	Erp29 P52555: Endoplasmic reticulum resident protein 29	5	11	9	**1.04**
211	Esyt1 Q9Z1X1: Extended synaptotagmin-1	5	9	5	**0.98**
212	Etf1 Q5U2Q7: Eukaryotic peptide chain release factor subunit 1	9	15	12	**1.12***
213	Etfa P13803: Electron transfer flavoprotein subunit alpha, mitochondrial	9	14	10	**0.88**
214	Etfb Q68FU3: Electron transfer flavoprotein subunit beta	10	16	13	**0.96**
215	Etfdh Q6UPE1: Electron transfer flavoprotein-ubiquinone oxidoreductase, mitochondrial	13	13	9	**1.08**
216	Ezr P31977: Ezrin	4	11	2	**0.94**
217	Fam129a Q9ESN0: Protein Niban	6	8	6	**1.14**
218	Fam213a Q6AXX6: Redox-regulatory protein FAM213A	5	14	11	**0.99**
219	Fam98a Q5FWT1: Protein FAM98A	5	8	6	**1.00**
220	Farsa Q505J8: Phenylalanine--tRNA ligase alpha subunit	5	7	5	**0.98**

#	protein	group	peptides	spectra	quant	ratio
221	Fasn P12785: Fatty acid synthase		2	2	2	**0.79**
222	Fga P06399: Fibrinogen alpha chain [Cleaved into: Fibrinopeptide A; Fibrinogen alpha chain]		3	6	5	**2.03***
223	Fgb P14480: Fibrinogen beta chain		4	5	3	**2.59***
224	Fgg P02680: Fibrinogen gamma chain		2	2	2	**2.22***
225	Fh P14408: Fumarate hydratase, mitochondrial		6	7	7	**0.90**
226	Fis1 P84817: Mitochondrial fission 1 protein		2	3	3	**1.04**
227	Flnc D3ZHA0: Filamin-C		2	4	3	**1.26**
228	Fn1 P04937: Fibronectin		2	3	3	**1.75***
229	Ftl1 P02793: Ferritin light chain 1		2	3	3	**0.84**
230	Gapdh P04797: Glyceraldehyde-3-phosphate dehydrogenase		8	19	17	**0.89**
231	Gars Q5I0G4: Glycine--tRNA ligase		2	6	5	**1.09**
232	Gatm P50442: Glycine amidinotransferase, mitochondrial		14	27	22	**0.95**
233	Gcg P06883: Glucagon [Cleaved into: Glicentin; Glicentin-related polypeptide		4	11	9	**0.64***
234	Gde1 Q9JL55: Glycerophosphodiester phosphodiesterase 1		2	2	2	**1.17***
235	Gdi2 P50399: Rab GDP dissociation inhibitor beta	1/2	19	36	26	**1.15**
236	Ggh Q62867: Gamma-glutamyl hydrolase		5	7	5	**0.87**
237	Ggt1 P07314: Gamma-glutamyltranspeptidase 1		8	16	13	**1.08**
238	Glg1 Q62638: Golgi apparatus protein 1		3	3	3	**1.13**
239	Gls2 P28492: Glutaminase liver isoform, mitochondrial		2	2	2	**0.97**
240	Glud1 P10860: Glutamate dehydrogenase 1, mitochondrial		21	53	47	**0.80**
241	Glul P09606: Glutamine synthetase		2	2	2	**0.81**
242	Gmppa Q5XIC1: Mannose-1-phosphate guanyltransferase alpha		3	7	6	**0.86**
243	Gnai2 P04897: Guanine nucleotide-binding protein G(i) subunit alpha-2		3	3	2	**1.11**
244	Gnai3 P08753: Guanine nucleotide-binding protein G(k) subunit alpha		2	2	1	**1.25***
245	Gnaq P82471: Guanine nucleotide-binding protein G(q) subunit alpha		2	3	3	**1.10**
246	Gnas P63095: Guanine nucleotide-binding protein G(s) subunit alpha isoforms short	1/3	4	4	1	**1.28**
247	Gnb1 P54311: Guanine nucleotide-binding protein G(I)/G(S)/G(T) subunit beta-1		5	9	2	**1.01**
248	Gnb2 P54313: Guanine nucleotide-binding protein G(I)/G(S)/G(T) subunit beta-2	1/2	8	13	3	**1.19**
249	Gnb2l1 P63245: Guanine nucleotide-binding protein subunit beta-2-like 1		16	68	63	**1.06**
250	Golph3 Q9ERE4: Golgi phosphoprotein 3		2	2	2	**0.98**
251	Gosr2 O35165: Golgi SNAP receptor complex member 2		3	6	4	**0.97**
252	Got2 P00507: Aspartate aminotransferase, mitochondrial		3	4	3	**1.00**

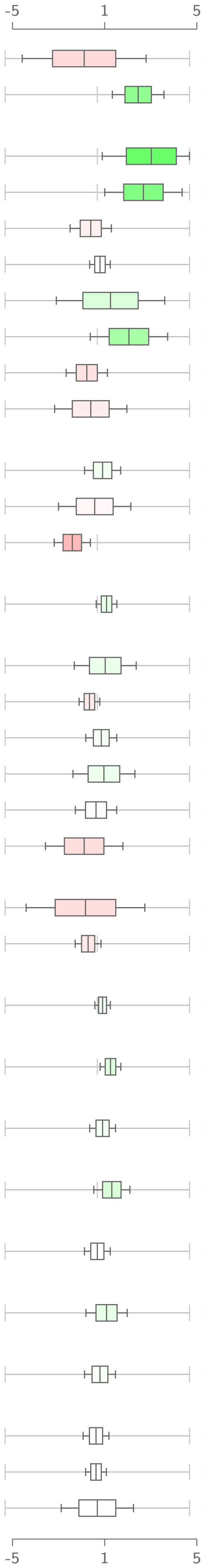

#	protein	group	peptides	spectra	quant	ratio
**253**	Gp2 P19218: Pancreatic secretory granule membrane major glycoprotein GP2		15	51	44	**1.09**
**254**	Gpd2 P35571: Glycerol-3-phosphate dehydrogenase, mitochondrial		2	3	3	**0.98**
**255**	Gpr108 Q6P6V6: Protein GPR108		2	2	2	**1.13**
**256**	Gstk1 P24473: Glutathione S-transferase kappa 1		3	3	3	**0.98**
**257**	Gstm2 P08010, Gstm3 P08009: Glutathione S-transferase Mu 2, Glutathione S-transferase Yb-3	2/2	2	2	2	**1.04**
**258**	Gstz1 P57113: Maleylacetoacetate isomerase		2	3	3	**1.01**
**259**	H1f0 P43278: Histone H1.0		2	3	3	**0.93**
**260**	Hadh Q9WVK7: Hydroxyacyl-coenzyme A dehydrogenase, mitochondrial		6	15	12	**0.79**
**261**	Hadha Q64428: Trifunctional enzyme subunit alpha, mitochondrial		23	59	55	**1.05**
**262**	Hadhb Q60587: Trifunctional enzyme subunit beta, mitochondrial		6	10	9	**1.02**
**263**	Hagh O35952: Hydroxyacylglutathione hydrolase, mitochondrial		2	3	3	**0.99**
**264**	Hba1 P01946: Hemoglobin subunit alpha-1/2		7	18	14	**0.98**
**265**	Hbb P02091: Hemoglobin subunit beta-1		7	31	13	**0.95**
**266**	Hdlbp Q9Z1A6: Vigilin		45	125	112	**1.04**
**267**	Hist1h1e P15865: Histone H1.4	1/2	5	7	4	**0.56**
**268**	Hist1h4b P62804: Histone H4 [Cleaved into: Osteogenic growth peptide		9	28	26	**0.74**
**269**	Hk1 P05708: Hexokinase-1		9	10	8	**0.98**
**270**	Hmgb1 P63159: High mobility group protein B1		3	3	3	**0.94**
**271**	Hmgcl P97519: Hydroxymethylglutaryl-CoA lyase, mitochondrial		2	2	2	**0.94**
**272**	Hmgcs2 P22791: Hydroxymethylglutaryl-CoA synthase, mitochondrial		2	3	3	**0.85**
**273**	Hnrnpa2b1 A7VJC2: Heterogeneous nuclear ribonucleoproteins A2/B1		2	2	1	**0.52***
**274**	Hnrnpa3 Q6URK4: Heterogeneous nuclear ribonucleoprotein A3		4	4	3	**0.65**
**275**	Hnrnpdl Q3SWU3: Heterogeneous nuclear ribonucleoprotein D-like		2	2	2	**0.96**
**276**	Hnrnpk P61980: Heterogeneous nuclear ribonucleoprotein K		8	9	9	**0.69***
**277**	Hnrnpm Q62826: Heterogeneous nuclear ribonucleoprotein M		7	9	7	**0.97**
**278**	Hpx P20059: Hemopexin		2	2	2	**3.05***
**279**	Hrg Q99PS8: Histidine-rich glycoprotein		3	4	3	**4.62***
**280**	Hsd17b10 O70351: 3-hydroxyacyl-CoA dehydrogenase type-2		10	28	23	**1.12**
**281**	Hsd17b11 Q6AYS8: Estradiol 17-beta-dehydrogenase 11		5	14	7	**1.18**

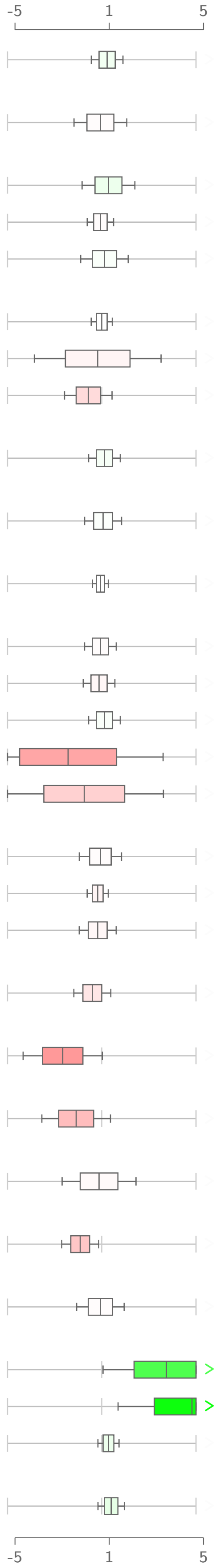

#	protein	group	peptides	spectra	quant	ratio
282	Hsd17b13 Q5M875: 17-beta-hydroxysteroid dehydrogenase 13		13	48	31	**1.04**
283	Hsd17b4 P97852: Peroxisomal multifunctional enzyme type 2		4	4	3	**1.14***
284	Hsp90ab1 P34058: Heat shock protein HSP 90-beta	1/2	8	9	3	**0.90**
285	Hsp90b1 Q66HD0: Endoplasmin		33	77	67	**1.07**
286	Hspa13 O35162: Heat shock 70 kDa protein 13		5	5	4	**1.06**
287	Hspa5 P06761: 78 kDa glucose-regulated protein		31	141	116	**1.04**
288	Hspa8 P63018: Heat shock cognate 71 kDa protein	1/4	19	40	26	**0.88***
289	Hspa9 P48721: Stress-70 protein, mitochondrial		10	23	21	**0.99**
290	Hspd1 P63039: 60 kDa heat shock protein, mitochondrial		26	87	67	**1.00**
291	Hspe1 P26772: 10 kDa heat shock protein, mitochondrial		3	5	3	**1.04**
292	Hyou1 Q63617: Hypoxia up-regulated protein 1		25	54	46	**1.01**
293	Idh2 P56574: Isocitrate dehydrogenase [NADP], mitochondrial		13	17	13	**1.16**
294	Ifrd1 P20695: Interferon-related developmental regulator 1		3	4	3	**1.91***
295	Igg-2a P20760: Ig gamma-2A chain C region		2	2	2	**1.95***
296	Immt Q3KR86: MICOS complex subunit Mic60		16	19	15	**1.10**
297	Ins1 P01322: Insulin-1 [Cleaved into: Insulin-1 B chain; Insulin-1 A chain]		4	5	3	**0.54**
298	Itgb1 P49134: Integrin beta-1		2	2	2	**1.21**
299	Ivd P12007: Isovaleryl-CoA dehydrogenase, mitochondrial		4	6	5	**0.93**
300	Jagn1 Q4KM64: Protein jagunal homolog 1		3	3	3	**0.42***
301	Jup Q6P0K8: Junction plakoglobin		3	5	3	**1.16**
302	Kif5b Q2PQA9: Kinesin-1 heavy chain		4	6	4	**0.90**
303	Krt1 Q6IMF3: Keratin, type II cytoskeletal 1	1/3	3	3	1	**0.96**
304	Krt10 Q6IFW6: Keratin, type I cytoskeletal 10		2	3	3	**0.68**
305	Krt18 Q5BJY9: Keratin, type I cytoskeletal 18		19	36	24	**0.97**
306	Krt19 Q63279: Keratin, type I cytoskeletal 19		2	4	1	**0.77**
307	Krt6a Q4FZU2: Keratin, type II cytoskeletal 6A	1/2	3	3	1	**1.04**
309	Krt8 Q10758: Keratin, type II cytoskeletal 8	1/2	18	31	22	**1.08**
310	Lamp1 P14562: Lysosome-associated membrane glycoprotein 1		3	8	6	**1.06**
311	Lamp2 P17046: Lysosome-associated membrane glycoprotein 2		2	3	3	**1.27***
312	Lancl1 Q9QX69: LanC-like protein 1		2	2	2	**1.07**
313	Lap3 Q68FS4: Cytosol aminopeptidase		5	8	6	**1.05**
314	Ldha P04642: L-lactate dehydrogenase A chain		5	5	3	**0.87**
315	Letm1 Q5XIN6: LETM1 and EF-hand domain-containing protein 1, mitochondrial		5	10	8	**0.99**
316	Lgals2 Q9Z144: Galectin-2		4	5	3	**1.33**
317	Lman1 Q62902: Protein ERGIC-53		14	23	18	**1.07**

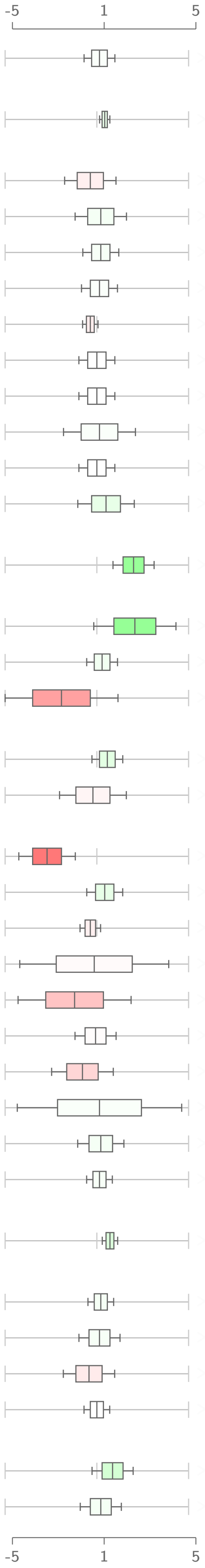

#	protein	group	peptides	spectra	quant	ratio
**318**	Lmf2 A1L1J9: Lipase maturation factor 2	2	2	2	**1.19**
**319**	Lmna P48679: Prelamin-A/C [Cleaved into: Lamin-A/C]	6	7	5	**0.91**
**320**	Lonp1 Q924S5: Lon protease homolog, mitochondrial	7	10	7	**1.16**
**321**	Lpcat3 Q5FVN0: Lysophospholipid acyltransferase 5	3	4	3	**1.16**
**322**	Lrpprc Q5SGE0: Leucine-rich PPR motif-containing protein, mitochondrial	4	4	3	**1.14**
**323**	Lrrc59 Q5RJR8: Leucine-rich repeat-containing protein 59	14	46	39	**0.97**
**324**	Magt1 O35777: Magnesium transporter protein 1	2	4	3	**1.06**
**325**	Man2a1 P28494: Alpha-mannosidase 2	7	7	6	**1.03**
**326**	Manf P0C5H9: Mesencephalic astrocyte-derived neurotrophic factor	11	28	25	**0.99**
**327**	Maoa P21396: Amine oxidase [flavin-containing] A	13	21	17	**1.20**
**328**	Mapre2 Q3B8Q0: Microtubule-associated protein RP/EB family member 2	3	4	3	**1.10**
**329**	Marc2 O88994: Mitochondrial amidoxime reducing component 2	6	6	4	**1.14**
**330**	Marcks P30009: Myristoylated alanine-rich C-kinase substrate	2	2	2	**0.71***
**331**	Matr3 P43244: Matrin-3	3	4	3	**1.02**
**332**	Mccc1 Q5I0C3: Methylcrotonoyl-CoA carboxylase subunit alpha, mitochondrial	18	33	26	**1.10**
**333**	Mccc2 Q5XIT9: Methylcrotonoyl-CoA carboxylase beta chain, mitochondrial	22	56	48	**1.08**
**334**	Mcfd2 Q8K5B3: Multiple coagulation factor deficiency protein 2 homolog	2	3	3	**0.30***
**335**	Mcpt1 P09650: Mast cell protease 1	2	2	2	**0.74**
**336**	Mcts1 Q4G009: Malignant T-cell-amplified sequence 1	2	3	3	**0.77***
**337**	Mdh2 P04636: Malate dehydrogenase, mitochondrial	14	41	34	**1.17**
**338**	Mgll Q8R431: Monoglyceride lipase	2	6	5	**1.15**
**339**	Mif P30904: Macrophage migration inhibitory factor	2	4	3	**1.03**
**340**	Mlec Q5FVQ4: Malectin	9	27	24	**1.05**
**341**	Mlycd Q920F5: Malonyl-CoA decarboxylase, mitochondrial	2	2	2	**0.58***
**342**	Mogs O88941: Mannosyl-oligosaccharide glucosidase	16	22	18	**1.05**
**343**	Mpc2 P38718: Mitochondrial pyruvate carrier 2	3	3	3	**1.07**
**344**	Msn O35763: Moesin	6	13	3	**0.90**
**345**	Mtco2 P00406: Cytochrome c oxidase subunit 2	3	8	5	**1.04**
**346**	Mthfd1 P27653: C-1-tetrahydrofolate synthase, cytoplasmic	10	13	9	**0.95**
**347**	Mtnd4 P05508: NADH-ubiquinone oxidoreductase chain 4	3	11	9	**1.19**
**348**	Mvp Q62667: Major vault protein	7	10	9	**1.08**
**349**	Mybbp1a O35821: Myb-binding protein 1A	5	9	6	**1.03**
**350**	Myh10 Q9JLT0: Myosin-10	8	12	3	**1.12**

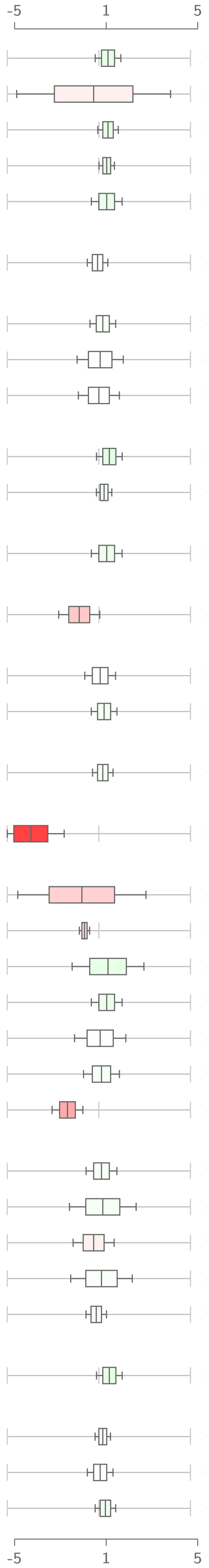

#	protein	group	peptides	spectra	quant	ratio
**351**	Myh9 Q62812: Myosin-9	1/2	31	44	25	**0.96**
**352**	Myl12b P18666, Rlc-a P13832: Myosin regulatory light chain 12B, Myosin regulatory light chain RLC-A	2/2	4	4	3	**0.97**
**353**	Myl6 Q64119: Myosin light polypeptide 6		7	8	7	**0.92**
**354**	Myo1c Q63355: Unconventional myosin-Ic		5	7	6	**1.10**
**355**	Napa P54921: Alpha-soluble NSF attachment protein		9	13	9	**1.07**
**356**	Nceh1 B2GV54: Neutral cholesterol ester hydrolase 1		5	6	5	**0.99**
**357**	Ncln Q5XIA1: Nicalin		13	20	15	**1.04**
**358**	Ndrg1 Q6JE36: Protein NDRG1		2	2	2	**1.27**
**359**	Ndufa10 Q561S0: NADH dehydrogenase [ubiquinone] 1 alpha subcomplex subunit 10, mitochondrial		4	6	4	**1.13**
**360**	Ndufa11 Q80W89: NADH dehydrogenase [ubiquinone] 1 alpha subcomplex subunit 11		3	3	3	**0.96**
**361**	Ndufa5 Q63362: NADH dehydrogenase [ubiquinone] 1 alpha subcomplex subunit 5		5	13	12	**1.13**
**362**	Ndufa9 Q5BK63: NADH dehydrogenase [ubiquinone] 1 alpha subcomplex subunit 9, mitochondrial		5	8	6	**1.05**
**363**	Ndufs1 Q66HF1: NADH-ubiquinone oxidoreductase 75 kDa subunit, mitochondrial		19	42	32	**1.18**
**364**	Ndufs2 Q641Y2: NADH dehydrogenase [ubiquinone] iron-sulfur protein 2, mitochondrial		8	9	5	**1.09**
**365**	Ndufs4 Q5XIF3: NADH dehydrogenase [ubiquinone] iron-sulfur protein 4, mitochondrial		4	6	4	**1.00**
**366**	Ndufs6 P52504: NADH dehydrogenase [ubiquinone] iron-sulfur protein 6, mitochondrial		2	3	3	**1.06**
**367**	Ndufv2 P19234: NADH dehydrogenase [ubiquinone] flavoprotein 2, mitochondrial		5	11	9	**1.13**
**368**	Nme2 P19804: Nucleoside diphosphate kinase B	1/2	7	23	8	**1.05**
**369**	Nmt1 Q8K1Q0: Glycylpeptide N-tetradecanoyltransferase 1		6	8	6	**1.09**
**370**	Nono Q5FVM4: Non-POU domain-containing octamer-binding protein		5	6	3	**1.58**
**371**	Npm1 P13084: Nucleophosmin		3	3	3	**0.92**
**372**	Nsf Q9QUL6: Vesicle-fusing ATPase		7	7	5	**1.05**
**373**	Nucb2 Q9JI85: Nucleobindin-2		10	13	11	**1.00**
**374**	Ociad1 Q5XIG4: OCIA domain-containing protein 1		2	2	2	**0.99**
**375**	Ogdh Q5XI78: 2-oxoglutarate dehydrogenase, mitochondrial		12	15	11	**0.98**
**376**	Opa1 Q2TA68: Dynamin-like 120 kDa protein, mitochondrial		7	7	6	**1.11**
**377**	Osbpl1a Q8K4M9: Oxysterol-binding protein-related protein 1		6	9	8	**1.09**
**378**	P01835: Ig kappa chain C region, B allele		3	5	3	**4.11***
**379**	P11517: Hemoglobin subunit beta-2		8	25	8	**1.07**
**380**	P32821, P32822: Trypsin V-A, Trypsin V-B	2/2	3	10	8	**1.27**

#	protein	group	peptides	spectra	quant	ratio
381	P4hb P04785: Protein disulfide-isomerase		35	136	110	**1.05**
382	P56571: ES1 protein homolog, mitochondrial		5	7	5	**1.07**
383	Pabpc1 Q9EPH8: Polyadenylate-binding protein 1		12	24	21	**0.67***
384	Pacsin2 Q9QY17: Protein kinase C and casein kinase substrate in neurons 2 protein		5	6	5	**1.19**
385	Paics P51583: Multifunctional protein ADE2 [Includes: Phosphoribosylaminoimidazole-succino . . .		2	2	2	**1.12**
386	Pc P52873: Pyruvate carboxylase, mitochondrial		11	18	16	**1.01**
387	Pcca P14882: Propionyl-CoA carboxylase alpha chain, mitochondrial		15	23	20	**1.03**
388	Pccb P07633: Propionyl-CoA carboxylase beta chain, mitochondrial		8	11	7	**1.08**
389	Pcyox1 Q99ML5: Prenylcysteine oxidase		3	4	3	**1.03**
390	Pdap1 Q62785: 28 kDa heat- and acid-stable phosphoprotein		10	14	9	**0.68**
391	Pdcd4 Q9JID1: Programmed cell death protein 4		2	2	2	**0.31***
392	Pdcd6ip Q9QZA2: Programmed cell death 6-interacting protein		2	2	2	**0.96**
393	Pdha1 P26284: Pyruvate dehydrogenase E1 component subunit alpha, somatic form, mitochondrial		7	11	9	**0.87**
394	Pdhb P49432: Pyruvate dehydrogenase E1 component subunit beta, mitochondrial		8	14	13	**0.95**
395	Pdia3 P11598: Protein disulfide-isomerase A3		25	62	53	**0.96**
396	Pdia4 P38659: Protein disulfide-isomerase A4		19	36	30	**1.02**
397	Pdia5 Q5I0H9: Protein disulfide-isomerase A5		6	7	6	**1.01**
398	Pdia6 Q63081: Protein disulfide-isomerase A6		17	51	42	**1.04**
399	Pecr Q9WVK3: Peroxisomal trans-2-enoyl-CoA reductase		7	11	8	**1.26**
400	Pelo Q5XIP1: Protein pelota homolog		2	2	2	**1.03**
401	Pfkl P30835: ATP-dependent 6-phosphofructokinase, liver type		2	2	2	**0.85***
402	Pgrmc1 P70580: Membrane-associated progesterone receptor component 1		4	7	6	**0.95**
403	Pgrmc2 Q5XIU9: Membrane-associated progesterone receptor component 2		4	4	3	**1.04**
404	Phb P67779: Prohibitin		14	33	28	**1.14**
405	Phb2 Q5XIH7: Prohibitin-2		11	35	31	**1.16**
406	Phgdh O08651: D-3-phosphoglycerate dehydrogenase		3	3	3	**1.08**
407	Pitpna P16446: Phosphatidylinositol transfer protein alpha isoform		2	3	3	**1.27**
408	Pla2g1b P04055: Phospholipase A2		4	6	5	**1.15**
409	Plg Q01177: Plasminogen		9	14	14	**1.59**
410	Pnlip P27657: Pancreatic triacylglycerol lipase		24	191	169	**1.11**
411	Pnliprp1 P54316: Inactive pancreatic lipase-related protein 1		21	73	65	**1.11**
412	Pnliprp2 P54318: Pancreatic lipase-related protein 2		19	62	55	**1.08**

#	protein	group	peptides	spectra	quant	ratio
413	Pon2 Q6AXM8: Serum paraoxonase/arylesterase 2		3	3	3	**1.11**
414	Por P00388: NADPH--cytochrome P450 reductase		3	3	3	**1.00**
415	Ppia P10111: Peptidyl-prolyl cis-trans isomerase A		10	20	15	**0.90**
416	Ppib P24368: Peptidyl-prolyl cis-trans isomerase B		11	21	18	**1.09**
417	Ppy P06303: Pancreatic prohormone		2	3	3	**0.84**
418	Prdx1 Q63716: Peroxiredoxin-1		7	15	9	**0.33***
419	Prdx4 Q9Z0V5: Peroxiredoxin-4		9	37	30	**0.93**
420	Prdx5 Q9R063: Peroxiredoxin-5, mitochondrial		2	3	3	**0.97**
421	Preb Q9WTV0: Prolactin regulatory element-binding protein		4	5	3	**1.05**
422	Prkar2a P12368: cAMP-dependent protein kinase type II-alpha regulatory subunit		2	2	2	**0.80**
423	Prrc1 Q3T1I4: Protein PRRC1		3	9	7	**0.79**
424	Prss1 P00762: Anionic trypsin-1		3	20	18	**1.02**
425	Prss2 P00763: Anionic trypsin-2		4	8	6	**0.96**
426	Psma1 P18420: Proteasome subunit alpha type-1		4	8	6	**0.86**
427	Psma2 P17220: Proteasome subunit alpha type-2		4	10	8	**0.89**
428	Psma4 P21670: Proteasome subunit alpha type-4		5	5	3	**0.81**
429	Psma5 P34064: Proteasome subunit alpha type-5		3	3	3	**0.85**
430	Psma6 P60901: Proteasome subunit alpha type-6		2	2	2	**0.83***
431	Psma7 P48004: Proteasome subunit alpha type-7		3	3	3	**0.89**
432	Psmb1 P18421: Proteasome subunit beta type-1		2	2	2	**0.85**
433	Psmb3 P40112: Proteasome subunit beta type-3		3	5	4	**0.97**
434	Psmb4 P34067: Proteasome subunit beta type-4		2	3	3	**0.91**
435	Psmb5 P28075: Proteasome subunit beta type-5		5	6	5	**1.08**
436	Psmb6 P28073: Proteasome subunit beta type-6		2	2	2	**0.92**
437	Psmb7 Q9JHW0: Proteasome subunit beta type-7		2	3	3	**0.86**
438	Psmc1 P62193: 26S protease regulatory subunit 4		3	3	3	**0.86**
439	Psmc2 Q63347: 26S protease regulatory subunit 7		4	4	3	**0.88**
440	Psmc3 Q63569: 26S protease regulatory subunit 6A		5	5	3	**0.92**
441	Psmc4 Q63570: 26S protease regulatory subunit 6B		4	7	7	**0.88**
442	Psmc5 P62198: 26S protease regulatory subunit 8		3	3	3	**0.92**
443	Psmd1 O88761: 26S proteasome non-ATPase regulatory subunit 1		3	3	3	**0.92**
444	Psmd11 F1LMZ8: 26S proteasome non-ATPase regulatory subunit 11		4	4	3	**0.98**
445	Psmd13 B0BN93: 26S proteasome non-ATPase regulatory subunit 13		4	5	3	**1.01**
446	Psmd2 Q4FZT9: 26S proteasome non-ATPase regulatory subunit 2		7	7	5	**0.98**
447	Ptbp1 Q00438: Polypyrimidine tract-binding protein 1	1/2	5	13	6	**0.97**
448	Ptpn6 P81718: Tyrosine-protein phosphatase non-receptor type 6		3	3	3	**0.90**

#	protein	group	peptides	spectra	quant	ratio
**449**	Ptrf P85125: Polymerase I and transcript release factor		3	5	4	**0.86**
**450**	Pycr2 Q6AY23: Pyrroline-5-carboxylate reductase 2		2	5	3	**1.18**
**451**	Q00715: Histone H2B type 1	1/2	6	18	8	**0.94**
**452**	Rab10 P35281: Ras-related protein Rab-10	1/2	4	9	2	**1.04**
**453**	Rab11b O35509: Ras-related protein Rab-11B	1/2	5	7	1	**1.13**
**454**	Rab14 P61107: Ras-related protein Rab-14		7	9	5	**0.97**
**455**	Rab18 Q5EB77: Ras-related protein Rab-18		7	10	9	**1.06**
**456**	Rab1A Q6NYB7: Ras-related protein Rab-1A	1/4	11	26	12	**1.06**
**457**	Rab1b P10536: Ras-related protein Rab-1B		7	17	3	**1.09**
**458**	Rab27a P23640: Ras-related protein Rab-27A		2	3	1	**0.98**
**459**	Rab27b Q99P74: Ras-related protein Rab-27B		3	5	3	**1.14***
**460**	Rab2a P05712: Ras-related protein Rab-2A		6	16	14	**1.04**
**461**	Rab3d Q63942: GTP-binding protein Rab-3D	1/4	7	13	6	**1.21**
**462**	Rab6a Q9WVB1: Ras-related protein Rab-6A		4	6	3	**1.11**
**463**	Rab7a P09527: Ras-related protein Rab-7a		8	12	11	**0.96**
**464**	Rab8a P35280: Ras-related protein Rab-8A		3	7	1	**1.29***
**465**	Rac1 Q6RUV5: Ras-related C3 botulinum toxin substrate 1		5	6	3	**1.04**
**466**	Rala P63322: Ras-related protein Ral-A		2	5	2	**1.13**
**467**	Ralb P36860: Ras-related protein Ral-B		2	4	1	**1.30***
**468**	Ran P62828: GTP-binding nuclear protein Ran		2	4	3	**1.06**
**469**	Rap1a P62836: Ras-related protein Rap-1A		6	9	1	**1.24***
**470**	Rap1b Q62636: Ras-related protein Rap-1b		6	10	2	**1.06**
**471**	Rars P40329: Arginine--tRNA ligase, cytoplasmic		20	34	27	**0.90**
**472**	Rbmx Q4V898: RNA-binding motif protein, X chromosome		2	2	1	**0.90**
**473**	Rbmxl1 D4AE41, Rbmxrtl P84586: RNA binding motif protein, X-linked-like-1, RNA-binding motif protein, X chromosome retrogene-like	2/2	2	2	1	**0.92**
**474**	Reep5 B2RZ37: Receptor expression-enhancing protein 5		8	21	20	**0.97**
**475**	Reg1 P10758: Lithostathine		2	7	5	**0.97**
**476**	Rheb Q62639: GTP-binding protein Rheb		2	2	2	**1.07**
**477**	Rhoa P61589: Transforming protein RhoA	1/2	6	14	9	**0.92**
**478**	Rhot2 Q7TSA0: Mitochondrial Rho GTPase 2		2	2	2	**1.19***
**479**	Rnase1 P00684: Ribonuclease pancreatic beta-type		5	16	14	**1.04**
**480**	Rpl10 Q6PDV7: 60S ribosomal protein L10		6	30	27	**1.05**
**481**	Rpl10a P62907: 60S ribosomal protein L10a		11	35	32	**0.98**
**482**	Rpl11 P62914: 60S ribosomal protein L11		5	23	19	**1.03**
**483**	Rpl12 P23358: 60S ribosomal protein L12		8	28	21	**1.02**
**484**	Rpl13 P41123: 60S ribosomal protein L13		8	25	19	**0.99**
**485**	Rpl13a P35427: 60S ribosomal protein L13a		7	28	25	**1.07**
**486**	Rpl14 Q63507: 60S ribosomal protein L14		5	19	18	**1.06**

#	protein	group	peptides	spectra	quant	ratio
**487**	Rpl15 P61314: 60S ribosomal protein L15		6	19	17	**1.12**
**488**	Rpl17 P24049: 60S ribosomal protein L17		6	23	19	**1.04**
**489**	Rpl18 P12001: 60S ribosomal protein L18		3	11	8	**1.06**
**490**	Rpl18a P62718: 60S ribosomal protein L18a		6	25	21	**1.04**
**491**	Rpl19 P84100: 60S ribosomal protein L19		4	9	6	**0.97**
**492**	Rpl22 P47198: 60S ribosomal protein L22		2	10	9	**1.07**
**493**	Rpl23 P62832: 60S ribosomal protein L23		6	33	26	**0.99**
**494**	Rpl23a P62752: 60S ribosomal protein L23a		9	37	33	**0.99**
**495**	Rpl24 P83732: 60S ribosomal protein L24		5	36	30	**0.96**
**496**	Rpl26 P12749: 60S ribosomal protein L26		5	10	9	**0.98**
**497**	Rpl27 P61354: 60S ribosomal protein L27		2	2	2	**0.98**
**498**	Rpl27a P18445: 60S ribosomal protein L27a		6	22	14	**1.13**
**499**	Rpl28 P17702: 60S ribosomal protein L28		2	10	8	**1.07**
**500**	Rpl29 P25886: 60S ribosomal protein L29		2	3	3	**0.99**
**501**	Rpl3 P21531: 60S ribosomal protein L3		13	38	31	**1.02**
**502**	Rpl30 P62890: 60S ribosomal protein L30		4	17	13	**0.98**
**503**	Rpl31 P62902: 60S ribosomal protein L31		7	24	22	**1.00**
**504**	Rpl32 P62912: 60S ribosomal protein L32		5	14	11	**1.04**
**505**	Rpl34 P11250: 60S ribosomal protein L34		2	3	3	**1.03**
**506**	Rpl35 P17078: 60S ribosomal protein L35		4	9	8	**0.99**
**507**	Rpl35a P04646: 60S ribosomal protein L35a		3	5	3	**1.02**
**508**	Rpl36 P39032: 60S ribosomal protein L36		3	6	5	**0.83***
**509**	Rpl36a P83883: 60S ribosomal protein L36a		5	7	5	**1.00**
**510**	Rpl37a-ps1 P61515: Putative 60S ribosomal protein L37a	1/2	4	6	2	**0.94**
**511**	Rpl38 P63174: 60S ribosomal protein L38		4	24	20	**1.08**
**512**	Rpl4 P50878: 60S ribosomal protein L4		15	45	39	**1.06**
**513**	Rpl5 P09895: 60S ribosomal protein L5		13	58	47	**1.10**
**514**	Rpl6 P21533: 60S ribosomal protein L6		9	25	21	**0.99**
**515**	Rpl7 P05426: 60S ribosomal protein L7		14	61	48	**1.05**
**516**	Rpl7a P62425: 60S ribosomal protein L7a		18	64	59	**1.08**
**517**	Rpl8 P62919: 60S ribosomal protein L8		5	16	13	**1.00**
**518**	Rpl9 P17077: 60S ribosomal protein L9		8	32	28	**1.08**
**519**	Rplp0 P19945: 60S acidic ribosomal protein P0		12	48	34	**0.99**
**520**	Rplp1 P19944: 60S acidic ribosomal protein P1		3	25	20	**1.04**
**521**	Rplp2 P02401: 60S acidic ribosomal protein P2		7	23	17	**0.98**
**522**	Rpn1 P07153: Dolichyl-diphosphooligosaccharide--protein glycosyltransferase subunit 1		33	94	78	**1.03**
**523**	Rpn2 P25235: Dolichyl-diphosphooligosaccharide--protein glycosyltransferase subunit 2		18	63	49	**1.03**
**524**	Rps10 P63326: 40S ribosomal protein S10		10	36	31	**0.87**
**525**	Rps11 P62282: 40S ribosomal protein S11		8	24	23	**1.04**
**526**	Rps12 P63324: 40S ribosomal protein S12		5	11	11	**0.79**

#	protein	group	peptides	spectra	quant	ratio
527	Rps13 P62278: 40S ribosomal protein S13		6	14	11	1.02
528	Rps14 P13471: 40S ribosomal protein S14		4	16	13	1.05
529	Rps15 P62845: 40S ribosomal protein S15		3	11	9	1.08
530	Rps15a P62246: 40S ribosomal protein S15a		5	13	13	0.83
531	Rps16 P62250: 40S ribosomal protein S16		7	21	19	1.02
532	Rps17 P04644: 40S ribosomal protein S17		10	46	42	1.11
533	Rps18 P62271: 40S ribosomal protein S18		6	21	20	1.01
534	Rps19 P17074: 40S ribosomal protein S19		10	31	28	0.98
535	Rps2 P27952: 40S ribosomal protein S2		13	59	52	1.06
536	Rps20 P60868: 40S ribosomal protein S20		6	18	15	1.04
537	Rps21 P05765: 40S ribosomal protein S21		3	5	4	1.05
538	Rps23 P62268: 40S ribosomal protein S23		6	21	17	1.03
539	Rps24 P62850: 40S ribosomal protein S24		3	26	21	1.02
540	Rps25 P62853: 40S ribosomal protein S25		4	16	13	1.00
541	Rps26 P62856: 40S ribosomal protein S26		2	14	8	0.87
542	Rps27a P62982: Ubiquitin-40S ribosomal protein S27a	1/4	7	21	3	0.78
543	Rps27l P24051: 40S ribosomal protein S27-like	1/2	4	22	1	1.09
544	Rps28 P62859: 40S ribosomal protein S28		2	7	7	0.81*
545	Rps3 P62909: 40S ribosomal protein S3		15	67	58	1.05
546	Rps3a P49242: 40S ribosomal protein S3a		17	60	51	1.04
547	Rps4x P62703: 40S ribosomal protein S4, X isoform		12	52	46	1.02
548	Rps5 P24050: 40S ribosomal protein S5 [Cleaved into: 40S ribosomal protein S5, N-terminal . . .		3	10	8	1.00
549	Rps6 P62755: 40S ribosomal protein S6		8	29	23	1.01
550	Rps7 P62083: 40S ribosomal protein S7		6	22	19	1.04
551	Rps8 P62243: 40S ribosomal protein S8		9	27	24	1.05
552	Rps9 P29314: 40S ribosomal protein S9		5	11	7	1.09
553	Rpsa P38983: 40S ribosomal protein SA		10	30	27	0.96
554	Rtcb Q6AYT3: tRNA-splicing ligase RtcB homolog		12	17	14	1.00
555	Rtn3 Q6RJR6: Reticulon-3		2	4	3	1.49
556	Sacm1l Q9ES21: Phosphatidylinositide phosphatase SAC1		8	12	10	1.00
557	Samm50 Q6AXV4: Sorting and assembly machinery component 50 homolog		4	6	5	1.16
558	Sar1b Q5HZY2: GTP-binding protein SAR1b		3	7	5	1.26
559	Sccpdh Q6AY30: Saccharopine dehydrogenase-like oxidoreductase		2	3	3	1.06
560	Scfd1 Q62991: Sec1 family domain-containing protein 1		4	5	4	0.97
561	Sdha Q920L2: Succinate dehydrogenase [ubiquinone] flavoprotein subunit, mitochondrial		13	32	30	1.09
562	Sdhb P21913: Succinate dehydrogenase [ubiquinone] iron-sulfur subunit, mitochondrial		8	11	7	1.09

#	protein	group	peptides	spectra	quant	ratio
**563**	Sec11a P42667: Signal peptidase complex catalytic subunit SEC11A	6	8	6	**1.12**
**564**	Sec11c Q9WTR7: Signal peptidase complex catalytic subunit SEC11C	7	17	14	**1.08**
**565**	Sec13 Q5XFW8: Protein SEC13 homolog	5	6	4	**0.84**
**566**	Sec22b Q4KM74: Vesicle-trafficking protein SEC22b	9	20	17	**1.00**
**567**	Sec31a Q9Z2Q1: Protein transport protein Sec31A	18	31	26	**0.91**
**568**	Sec61a1 P61621: Protein transport protein Sec61 subunit alpha isoform 1	9	31	24	**1.03**
**569**	Sel1l Q80Z70: Protein sel-1 homolog 1	13	24	23	**1.13**
**570**	Sept7 Q9WVC0: Septin-7	2	2	2	**0.79**
**571**	Serbp1 Q6AXS5: Plasminogen activator inhibitor 1 RNA-binding protein	9	18	17	**0.86**
**572**	Serpinb1a Q4G075: Leukocyte elastase inhibitor A	2	3	3	**0.92**
**573**	Serpinh1 P29457: Serpin H1	10	14	10	**0.97**
**574**	Sfxn1 Q63965: Sideroflexin-1	7	13	10	**1.15***
**575**	Sfxn3 Q9JHY2: Sideroflexin-3	2	6	3	**1.31***
**576**	Sgpl1 Q8CHN6: Sphingosine-1-phosphate lyase 1	2	3	3	**1.16**
**577**	Slc25a1 P32089: Tricarboxylate transport protein, mitochondrial	4	5	3	**1.11**
**578**	Slc25a11 P97700: Mitochondrial 2-oxoglutarate/malate carrier protein	7	14	12	**1.22***
**579**	Slc25a3 P16036: Phosphate carrier protein, mitochondrial	8	9	7	**1.16**
**580**	Slc25a4 Q05962: ADP/ATP translocase 1	8	46	2	**0.87**
**581**	Slc25a42 P0C546: Mitochondrial coenzyme A transporter SLC25A42	2	3	3	**1.12**
**582**	Slc25a5 Q09073: ADP/ATP translocase 2	11	72	23	**1.11**
**583**	Slc27a1 P97849: Long-chain fatty acid transport protein 1	2	3	3	**0.79**
**584**	Slc33a1 Q6AYY8: Acetyl-coenzyme A transporter 1	6	13	8	**1.05**
**585**	Slc35b1 Q6V7K3: Solute carrier family 35 member B1	2	6	3	**1.19**
**586**	Slc38a5 A2VCW5: Sodium-coupled neutral amino acid transporter 5	4	7	6	**1.24***
**587**	Slc3a2 Q794F9: 4F2 cell-surface antigen heavy chain	12	34	31	**1.20**
**588**	Slc7a5 Q63016: Large neutral amino acids transporter small subunit 1	4	6	4	**1.04**
**589**	Slc7a8 Q9WVR6: Large neutral amino acids transporter small subunit 2	3	4	3	**1.12**
**590**	Smpd2 Q9ET64: Sphingomyelin phosphodiesterase 2	2	2	2	**1.06**
**591**	Snd1 Q66X93: Staphylococcal nuclease domain-containing protein 1	44	131	119	**0.96**
**592**	Snx5 B1H267: Sorting nexin-5	2	2	2	**0.95**
**593**	Soat1 O70536: Sterol O-acyltransferase 1	2	3	3	**1.02**
**594**	Sod1 P07632: Superoxide dismutase [Cu-Zn]	2	2	2	**0.94**
**595**	Sod2 P07895: Superoxide dismutase [Mn], mitochondrial	3	11	9	**1.32**

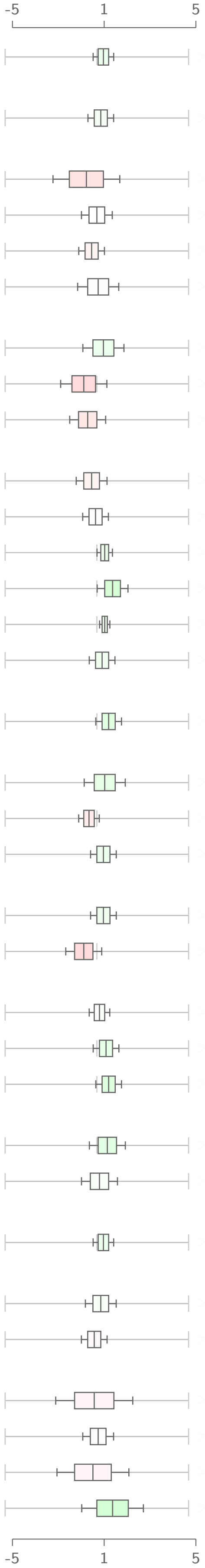

#	protein	group	peptides	spectra	quant	ratio
**596**	Spink3 P09656: Serine protease inhibitor Kazal-type 3	2	4	3	**0.94**
**597**	Sptan1 P16086: Spectrin alpha chain, non-erythrocytic 1	17	19	17	**1.04**
**598**	Srp54 Q6AYB5: Signal recognition particle 54 kDa protein	13	18	18	**0.98**
**599**	Srprb Q4FZX7: Signal recognition particle receptor subunit beta	10	28	24	**1.05**
**600**	Ssb P38656: Lupus La protein homolog	8	20	15	**1.02**
**601**	Ssbp1 P28042: Single-stranded DNA-binding protein, mitochondrial	4	9	8	**1.07**
**602**	Ssr1 Q7TPJ0: Translocon-associated protein subunit alpha	3	8	6	**0.96**
**603**	Ssr3 Q08013: Translocon-associated protein subunit gamma	2	8	7	**0.98**
**604**	Ssr4 Q07984: Translocon-associated protein subunit delta	5	25	20	**1.13**
**605**	Stoml2 Q4FZT0: Stomatin-like protein 2, mitochondrial	6	7	5	**1.08**
**606**	Stx18 Q68FW4: Syntaxin-18	3	3	3	**1.08**
**607**	Suclg1 P13086: Succinyl-CoA ligase [ADP/GDP-forming] subunit alpha, mitochondrial	8	11	9	**1.03**
**608**	Sycn O35775: Syncollin	5	21	19	**1.30**
**609**	Syncrip Q7TP47: Heterogeneous nuclear ribonucleoprotein Q	8	10	7	**1.06**
**610**	Tars Q5XHY5: Threonine--tRNA ligase, cytoplasmic	2	2	1	**1.11**
**611**	Tars2 Q68FW7: Threonine--tRNA ligase, mitochondrial	2	2	1	**1.05**
**612**	Tcp1 P28480: T-complex protein 1 subunit alpha	5	6	5	**1.07**
**613**	Tecr Q64232: Very-long-chain enoyl-CoA reductase	5	6	5	**1.12**
**614**	Tes Q2LAP6: Testin	4	8	7	**0.90**
**615**	Tf P12346: Serotransferrin	6	8	8	**1.64**
**616**	Timm8a Q9WVA1: Mitochondrial import inner membrane translocase subunit Tim8 A	2	3	3	**0.96**
**617**	Tkt P50137: Transketolase	3	3	3	**0.72***
**618**	Tm9sf2 Q66HG5: Transmembrane 9 superfamily member 2	5	7	6	**1.06**
**619**	Tm9sf4 Q4KLL4: Transmembrane 9 superfamily member 4	4	4	3	**1.08**
**620**	Tmbim6 P55062: Bax inhibitor 1	2	4	3	**0.90**
**621**	Tmed10 Q63584: Transmembrane emp24 domain-containing protein 10	9	20	16	**1.04**
**622**	Tmed2 Q63524: Transmembrane emp24 domain-containing protein 2	7	18	14	**1.10**
**623**	Tmed3 Q6AY25: Transmembrane emp24 domain-containing protein 3	3	7	5	**1.15**
**624**	Tmed7 D3ZTX0: Transmembrane emp24 domain-containing protein 7	2	5	4	**1.03**
**625**	Tmed9 Q5I0E7: Transmembrane emp24 domain-containing protein 9	6	8	7	**1.10**

#	protein	group	peptides	spectra	quant	ratio
626	Tmem109 Q6AYQ4: Transmembrane protein 109		3	5	3	**1.00**
627	Tmem214 A1L1L2: Transmembrane protein 214		10	16	12	**1.06**
628	Tpd52l2 Q6PCT3: Tumor protein D54		6	8	7	**0.92**
629	Tpi1 P48500: Triosephosphate isomerase		2	2	2	**1.03**
630	Tpm2 P58775, Tpm4 P09495: Tropomyosin beta chain, Tropomyosin alpha-4 chain	2/2	2	2	1	**1.04**
631	Tpm3 Q63610: Tropomyosin alpha-3 chain		2	2	1	**0.95**
632	Tpt1 P63029: Translationally-controlled tumor protein		2	2	2	**0.50***
633	Tram1 Q5XI41: Translocating chain-associated membrane protein 1		5	19	16	**1.09**
634	Trap1 Q5XHZ0: Heat shock protein 75 kDa, mitochondrial		4	4	3	**0.93**
635	Try3 P08426: Cationic trypsin-3		5	26	22	**1.24**
636	Tst P24329: Thiosulfate sulfurtransferase		5	8	7	**0.97**
637	Ttgn1 P19814: Trans-Golgi network integral membrane protein TGN38		2	2	2	**1.03**
640	Tuba4a Q5XIF6: Tubulin alpha-4A chain		16	49	7	**1.05**
643	Tubb4b Q6P9T8: Tubulin beta-4B chain		14	36	3	**1.04**
644	Tubb5 P69897: Tubulin beta-5 chain		15	46	11	**1.01**
645	Tufm P85834: Elongation factor Tu, mitochondrial		16	27	24	**1.05**
646	Uba5 Q5M7A4: Ubiquitin-like modifier-activating enzyme 5		2	3	3	**1.03**
647	Ube2n Q9EQX9: Ubiquitin-conjugating enzyme E2 N		2	2	2	**0.82**
648	Ubr4 Q2TL32: E3 ubiquitin-protein ligase UBR4		3	3	3	**1.01**
649	Ubxn4 Q5HZY0: UBX domain-containing protein 4		3	3	3	**1.05**
650	Ufl1 B2GV24: E3 UFM1-protein ligase 1		21	38	32	**1.04**
651	Ufm1 Q5BJP3: Ubiquitin-fold modifier 1		2	6	5	**1.00**
652	Ufsp2 Q5XIB4: Ufm1-specific protease 2		5	11	8	**0.93**
653	Uggt1 Q9JLA3: UDP-glucose:glycoprotein glucosyltransferase 1		17	29	23	**0.99**
654	Uqcrc1 Q68FY0: Cytochrome b-c1 complex subunit 1, mitochondrial		10	14	11	**1.10**
655	Uqcrc2 P32551: Cytochrome b-c1 complex subunit 2, mitochondrial		9	23	20	**1.12***
656	Uqcrfs1 P20788: Cytochrome b-c1 complex subunit Rieske, mitochondrial		5	13	10	**1.07**
657	Uqcrh Q5M9I5: Cytochrome b-c1 complex subunit 6, mitochondrial		4	8	7	**1.05**
658	Uso1 P41542: General vesicular transport factor p115		2	2	2	**0.97**
659	Vac14 Q80W92: Protein VAC14 homolog		2	5	3	**1.11**
660	Vamp8 Q9WUF4: Vesicle-associated membrane protein 8		2	3	3	**1.09**
661	Vapa Q9Z270: Vesicle-associated membrane protein-associated protein A		5	7	6	**1.06**
662	Vapb Q9Z269: Vesicle-associated membrane protein-associated protein B		2	3	3	**0.82**

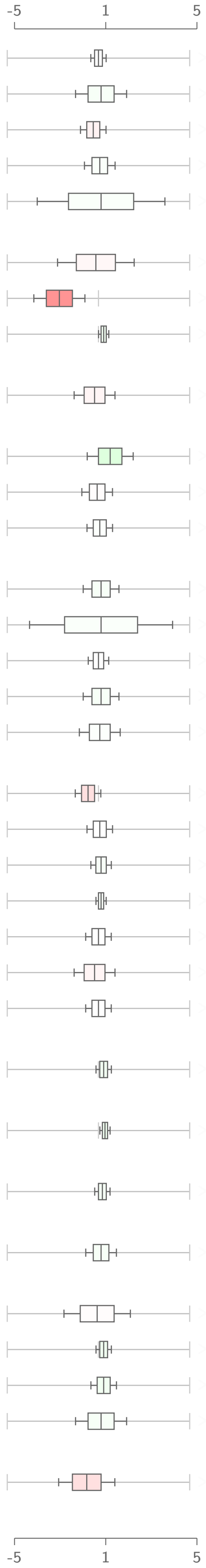

#	protein	group	peptides	spectra	quant	ratio
663	Vars Q04462: Valine--tRNA ligase		14	19	14	**0.84**
664	Vat1 Q3MIE4: Synaptic vesicle membrane protein VAT-1 homolog		10	19	16	**1.01**
665	Vcp P46462: Transitional endoplasmic reticulum ATPase		23	38	32	**0.89**
666	Vdac1 Q9Z2L0: Voltage-dependent anion-selective channel protein 1		10	33	26	**1.19**
667	Vdac2 P81155: Voltage-dependent anion-selective channel protein 2		9	26	17	**1.13**
668	Vdac3 Q9R1Z0: Voltage-dependent anion-selective channel protein 3		10	20	12	**1.19**
669	Vim P31000: Vimentin		3	6	3	**0.63**
670	Vkorc1 Q6TEK4: Vitamin K epoxide reductase complex subunit 1		2	10	8	**1.08**
671	Vkorc1l1 Q6TEK3: Vitamin K epoxide reductase complex subunit 1-like protein 1		3	4	3	**1.08**
672	Vps29 B2RZ78: Vacuolar protein sorting-associated protein 29		5	6	4	**0.73***
673	Vps45 O08700: Vacuolar protein sorting-associated protein 45		2	2	2	**0.84**
674	Wars Q6P7B0: Tryptophan--tRNA ligase, cytoplasmic		5	5	4	**0.94**
675	Wdr61 Q4V7A0: WD repeat-containing protein 61 [Cleaved into: WD repeat-containing protein ...		3	4	3	**0.83**
676	Yars Q4KM49: Tyrosine--tRNA ligase, cytoplasmic		15	19	14	**0.96**
677	Yars2 Q5I0L3: Tyrosine--tRNA ligase, mitochondrial		2	2	2	**1.19***
678	Ybx1 P62961: Nuclease-sensitive element-binding protein 1		4	9	5	**0.97**
679	Ybx3 Q62764: Y-box-binding protein 3		3	5	2	**1.00**
680	Ywhae P62260: 14-3-3 protein epsilon		5	11	4	**0.88**
681	Ywhag P61983: 14-3-3 protein gamma [Cleaved into: 14-3-3 protein gamma, N-terminally proce ...	1/3	5	9	2	**0.74**
682	Ywhaq P68255: 14-3-3 protein theta		4	8	1	**0.78**
683	Ywhaz P63102: 14-3-3 protein zeta/delta		5	10	3	**0.95**
684	Zc3h15 Q6U6G5: Zinc finger CCCH domain-containing protein 15		6	9	7	**0.98**
685	Zg16 Q8CJD3: Zymogen granule membrane protein 16		6	20	17	**1.13**
686	Zw10 Q4V8C2: Centromere/kinetochore protein zw10 homolog		2	6	5	**1.03**

2.2 Not Quantified Proteins

Number of identified but not quantified proteins:

#	protein	group	peptides	spectra
638	Tuba1a P68370: Tubulin alpha-1A chain	1/4	15	45
641	Tubb2a P85108, Tubb2b Q3KRE8: Tubulin beta-2A chain, Tubulin beta-2B chain	2/2	12	32
642	Tubb3 Q4QRB4: Tubulin beta-3 chain		8	20

#	protein	group	peptides	spectra
308	Krt75 Q6IG05: Keratin, type II cytoskeletal 75		3	4
639	Tuba1b Q6P9V9: Tubulin alpha-1B chain		15	51
